# Material gradients in gastropod radulae and their biomechanical significance: a combined approach on the paludomid *Lavigeria grandis*

**DOI:** 10.1007/s00114-022-01822-9

**Published:** 2022-11-02

**Authors:** Wencke Krings, Yoko Matsumura, Jan-Ole Brütt, Stanislav N. Gorb

**Affiliations:** 1 Department of Behavioral Biology, Institute of Cell and Systems Biology of Animals, Universität Hamburg, Martin-Luther-King-Platz 3, 20146 Hamburg, Germany; 2 Department of Mammalogy and Palaeoanthropology, Leibniz Institute for the Analysis of Biodiversity Change, Martin-Luther-King-Platz 3, 20146 Hamburg, Germany; 3 Department of Functional Morphology and Biomechanics, Zoologisches Institut, Christian-Albrechts-Universität Zu Kiel, Am Botanischen Garten 1-9, 24118 Kiel, Germany; 4 Department of General and Systematic Zoology, Zoological Institute and Museum, Universität Greifswald, Loitzer Str. 26, 17489 Greifswald, Germany

**Keywords:** Mollusca, Elemental composition, Biomineralization, Feeding, Lake Tanganyika, CLSM, Autofluorescence

## Abstract

The radula, a chitinous membrane spiked with teeth, is the molluscan autapomorphy for the gathering and processing of food. The teeth, as actual interfaces between the organism and the ingesta, act as load transmitting regions and have to withstand high stresses during foraging — without structural failure or high degrees of wear. Mechanisms contributing to this were studied previously in paludomid gastropods from Lake Tanganyika. For some species, gradients in hardness and Young’s modulus along the teeth were detected, enabling the bending and relying of teeth onto the next row, distributing the stresses more equally. The here presented study on one of them — *Lavigeria grandis* — aims at shedding light on the origin of these functional gradients. The mechanical properties were identified by nanoindentation technique and compared to the elemental composition, determined by elemental dispersive X-ray spectroscopy (EDX, EDS). This was done for the complete radular (mature and immature tooth rows), resulting in overall 236 EDX and 700 nanoindentation measurements. Even though teeth showed regional differences in elemental composition, we could not correlate the mechanical gradients with the elemental proportions. By applying confocal laser scanning microscopy (CLSM), we were finally able to relate the mechanical properties with the degree of tanning. CLSM is a common technique used on arthropod cuticle, but was never applied on radular teeth before. In general, we found that nanoindentation and CLSM techniques complement one another, as for example, CLSM is capable of revealing heterogeneities in material or micro-gradients, which leads to a better understanding of the functionalities of biological materials and structures.

**Supplementary Information:**

The online version contains supplementary material available at 10.1007/s00114-022-01822-9.

## Introduction

### The radula — an important molluscan autapomorphy

Mollusca represent the second most specious animal phylum, with its taxa occupying various ecological niches. The molluscs’ evolutionary successes probably relate to the diversity of their mouthparts (radula). This important molluscan autapomorphy can consist of a chitinous membrane with longitudinal and transversal rows of embedded teeth, which interact with the ingesta (i.e., food, minerals, feeding substrate = the substrate the food is attached to, everything that is taken in). Multiple interactions with ingesta can lead to wear and structural failure (Runham and Thornton 1967; Mackenstedt and Märkel 1987; Shaw et al. 2010; Krings and Gorb 2021b; Krings et al. 2021a), but teeth and membrane are constantly secreted by over- and underlain epithelia in the “radular sac” and become maturated in the “maturation zone”, before they enter the “working zone”, which is the actively used region of the radula.

### Failure preventing mechanisms and functional gradients

Even though some structures are constantly renewed (Runham 1963; Runham and Isarankura 1966; Mackenstedt and Märkel 1987, 2001), it was previously determined that multiple, partially interacting mechanisms reduce structural failure and wear. Besides the radular supporting structures underneath the teeth (i.e. membrane, muscles and odontophoral cartilages), which reduce the stresses (Kehl et al. 2019; Montroni et al. 2019; Krings et al. 2021a, b; Krings and Gorb 2021b), the structural organisation of the radular matrix itself contributes, too. The radula consists of alpha chitin fibres with associated proteins (Lowenstam and Weiner 1985; Guralnick and Smith 1999; Krings et al. 2020a), which show regional, load-depending differences in, e.g. fibre-orientation, -density, and -arrangement or/and in the degree of tanning (Runham 1963; Shaw et al. 2010; Montroni et al. 2019; Evans et al. 1990, 1994; Wealthall et al. 2005; Grunenfelder et al. 2014; Krings et al. 2022b). In addition, the presence of multiple tooth rows contributes to failure prevention, as mature teeth interlock in contact with the substrate, and, by this, distributes the stress more uniformly. For the taenioglossan radulae (i.e. with seven teeth per tooth row: one central tooth, flanked to each side by one lateral and two marginals) of paludomid gastropods, we found that this interlocking, which we called *collective effect,* is enabled by (1) tooth morphology and arrangement (for paludomids, see Krings et al. 2021b, c, d; Krings and Gorb 2021a; for interlocking in other taxa, see Solem 1972; Hickman 1980, 1984; Morris and Hickman 1981; Padilla 2003; Montroni et al. 2019; Ukmar-Godec et al. 2015; Herrera et al. 2015) and (2) the biomechanical behaviour of teeth and the embedding membrane. This latter depends on (A) the water-content of structures (Krings et al. 2021c, d) and (B) the gradients in hardness and Young’s modulus along each tooth (Krings et al. 2019, 2020b, 2021e, f; Gorb and Krings 2021). Here, the tooth basis is the softest and most flexible region, followed by the stylus and finally the cusp being the stiffest and hardest region. The degree of failure prevention by interlocking was also found to reflect trophic adaptations to the feeding substrate (rock, sand, mud, mixed surfaces). Species foraging on harder surfaces (rocks; e.g. *Lavigeria grandis* (Smith 1881) [Gastropoda: Paludomidae]) possess teeth with pronounced gradients in mechanical properties, forming a stiff array when loaded. In contrast, species feeding from compliant and soft surfaces (sand, mud) display softer and more homogenous teeth that do not interlock tightly. Additionally, we previously determined functional specialisations of specific tooth types for each ecological niche (Krings et al. 2019, 2020b, 2021e, f; Gorb and Krings 2021): solid substrate feeders (e.g. *Lavigeria grandis*) possess stiffer and harder central and lateral teeth, which are probably used for loosening algae from rocks, and softer marginal teeth, which probably collect the loosened particles in a complex radular motion afterwards (*multifunctional radula*). This is in contrast to the soft substrate feeders, which possess homogeneous teeth that are probably all equally engaged in the collection of loose particles (*monofunctional radula*).

Mechanical property gradients of radular teeth, contributing to function, were previously also documented in limpets and chitons. For these, the presence of high proportions of inorganic components (i.e. iron, silicon, and calcium) leads to harder and stiffer tooth cusps and their absence to softer and more flexible tooth styli and bases (e.g. Runham et al. 1969; Vincent 1980; van der Wal et al. 1999; Weaver et al. 2010; Grunenfelder et al. 2014; Barber et al. 2015; Krings et al. 2022c; for throughout reviews, see Brooker and Shaw 2012; Faivre and Ukmar-Godec 2015; Joester and Brooker 2016). In addition, the degree of cross-linking seems to contribute to the functional gradients in the mechanical properties in these taxa (Runham 1963; Ukmar-Godec et al. 2017).

### Aim of the study

For the paludomid gastropod *Lavigeria grandis*, we previously examined the nanostructure of the material by scanning electron microscopy (SEM) (Krings et al. 2022b) and the elemental composition by EDX (Krings et al. 2022a) of the mature radular teeth. The teeth are composed of layers with distinct fibre densities and orientations and show regional differences in the mineral content: the outer, very thin layer of the tooth leading edge (the edge that interacts with the ingesta) contains higher proportions of Ca (~ 7%) (Krings et al. 2022b). This probably reduces wear caused by abrasion, when loaded. The tooth core and the tooth trailing edge (the edge that does not interact with the ingesta) contain however very small proportions of Ca (~ 2–1%) and can thus deform more easily, reducing stress. However, no significant differences in the elemental composition, which would explain the differences in the mechanical properties detected, could be determined for the inner tooth structure (Gorb & Krings 2021; Krings et al. 2022a).

In the search for the origins of the mechanical property gradients, we now apply confocal laser scanning microscopy (CLSM) technique onto the teeth of *Lavigeria grandis*. This method was previously used to determine the degree of tanning in insect cuticula. We here document the complete radula by CLSM, which was never done before, and complement this data by EDX analyses and mechanical property testing on the distinct radular stages (radular sac, two regions in the maturation zone and working zone). We detected different autofluorescence signals, along each tooth and within each tooth row, and could show that they reflect the mechanical property gradients measured. In contrast, the mineral content does not seem to contribute significantly to the regional differences in hardness and Young’s modulus. We therefore conclude that the gradients have their origin in the degree of tanning, similar to insect cuticle, but distinct from limpets (Patellogastropoda) and chiton (Polyplacophora) radular teeth, where the mechanical properties strongly rely on the mineralization degree.

## Materials and methods

**Fig. 1:**
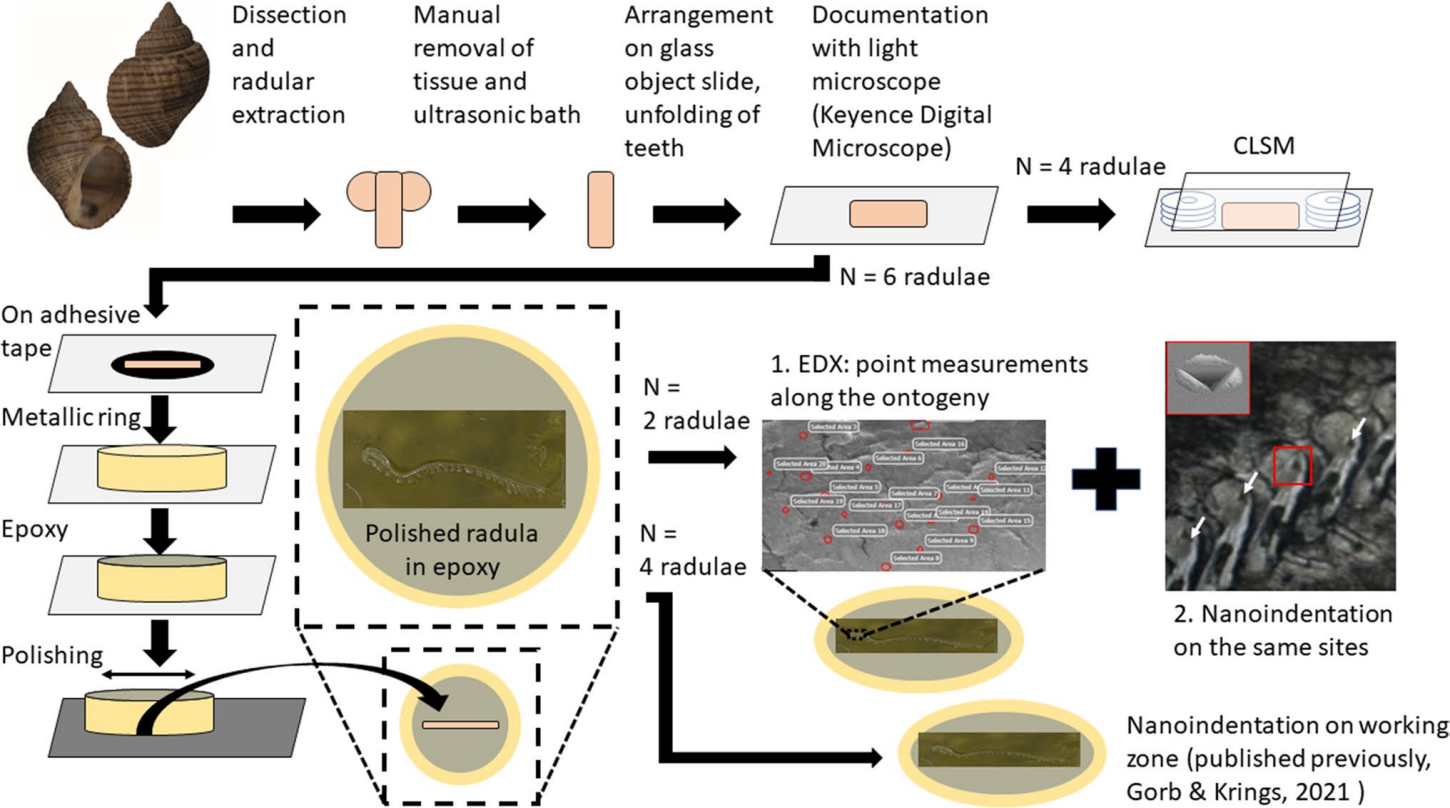
Experimental set-up and protocols for each radula studied

Please see Fig. 1 for a comprehensive summary of all methods used.

### Samples

**Fig. 2:**
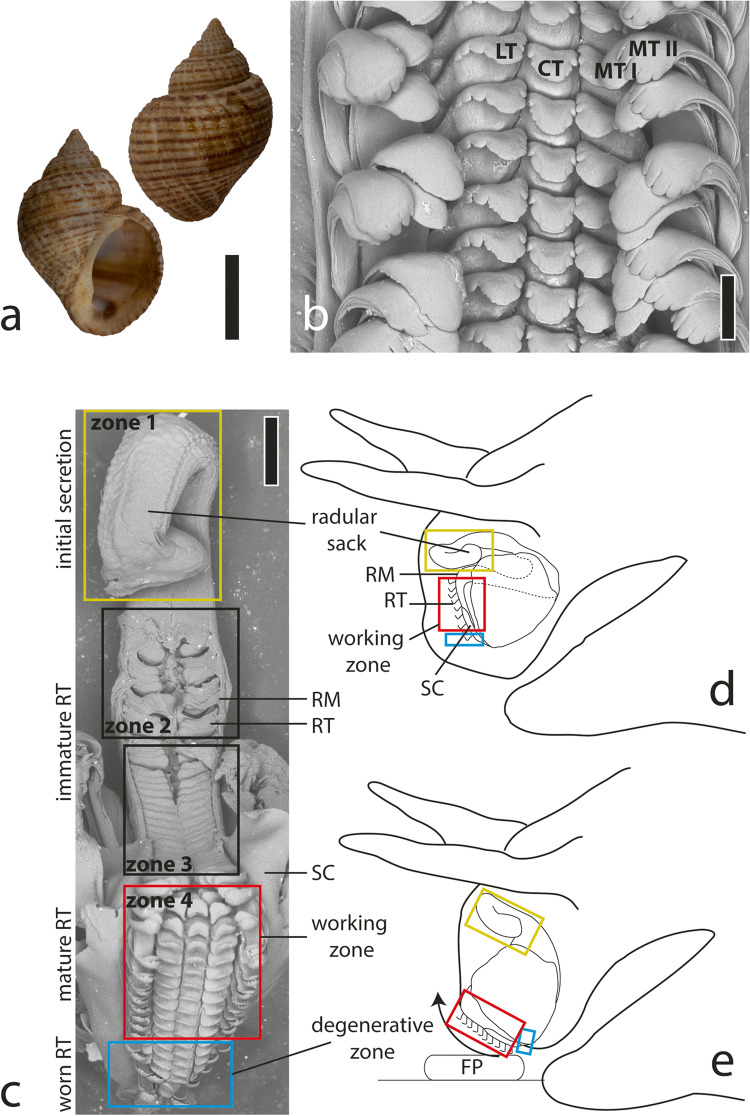
**a** Habitus image in ventral and dorsal view from one representative specimen (image from Gorb and Krings 2021). **b** Scanning electron microscopy (SEM) image of one representative radular working zone displaying the distinct radular tooth types (image from Krings et al. 2021f). **c** SEM image of one radula with highlighted ontogenetic zones 1–4, adapted from Krings and Gorb (2021a). **d** Schematic illustration of the buccal mass at rest and **e** during feeding action. CT, central tooth; FP, food particle; LT, lateral tooth; MT I, marginal tooth I (inner marginal tooth); MT II, marginal tooth II (outer marginal tooth); RM, radular membrane; RT, radular tooth; SC, subradular cartilage. Scale bars: **a** 10 mm; **b** 125 µm

The gastropods (Fig. 2a) were collected at Lake Tanganyika in Zambia (08°43′ 25″ S, 31°09′ 00″ E) on 30 Nov. 2017 by Heinz Büscher. Shells were cracked and gastropods killed with 70% EtOH and fixed herein, stored and inventoried at the malacological collection of the Zoological Museum in Hamburg (collection number: 150020/999), which is now part of the Leibniz-Institut zur Analyse des Biodiversitätswandels (LIB). After dissection, the radulae were carefully extracted from adult specimens, and the surrounding tissues (including the overlain epithelia) were removed by tweezers. Subsequently, radulae were cleaned by an ultrasonic bath, lasting approximately 5 s.

### Radular zones and documentation

All radulae (*n* = 10) were first deposited on glass object slides (Carl Roth, Karlsruhe, Germany) and carefully unfolded by tweezers. They were documented employing a Keyence Digital Microscope VHX-7000 (KEYENCE, Neu-Isenburg, Germany) equipped with automatic stacking software. Radular zones were defined by the presence of overlain epithelia or structure consistency. The zone without overlain epithelia was defined as the “working zone” (zone 4), i.e. fully mature teeth, and the zone with very fragile structures as “radular sac” (zone 1), i.e. the zone of initial secretion. The large area between these zones was divided into two areas of the same size (zones 2 and 3). Zones 1, 2 and 3 were covered by epithelia, which was manually removed. Then, four radulae were used for confocal laser scanning microscopy (CLSM) and six for embedment into the epoxy (nanoindentation and EDX).

### CLSM

For visualization of the autofluorescence, four radulae were used; each was arranged on one object glass slide. For this purpose, transparent self-adhesive reinforcement rings were glued to each slide, arranged around the radula. Eight reinforcement rings were stacked to avoid later contact between sample and cover slip following Michels and Büntzow (2010). Glycerine (greater than or equal to 99.5%, free of water, Carl Roth GmbH & Co. KG, Karlsruhe, Germany) was drippled onto the radula until it was completely covered. Finally, a glass cover slip was deposited on the sample, which was left for more than 1 h before visualisation.

Radulae were visualised employing a Zeiss LSM 700 confocal laser scanning microscope (Carl Zeiss Microscopy GmbH, Jena, Germany) following the protocol of Michels and Gorb (2012) that previously applied CLSM to arthropod cuticula. To visualise the autofluorescence, four stable solid-state lasers were used (wavelengths of 405 nm, 488 nm, 555 nm and 639 nm). To detect emitted autofluorescence, we applied bandpass or longpass emission filters, transmitting light of wavelengths 420–480 nm, greater than or equal to 490 nm, greater than or equal to 560 nm or greater than or equal to 640 nm, following the established protocol (Michels and Gorb 2012). Objective lens either with × 5 (Zeiss Plan-Apochromat, numerical aperture (NA) = 0.16), × 10 (Zeiss EC Plan-Neofluar, NA = 0.45) or × 20 (Zeiss Plan-Apochromat, air immersion, NA = 0.8) magnification were applied. Colours, blue, green, red (50% saturation) and red (50% saturation), were assigned to each image produced with the above-mentioned four sets of lasers and filters. Subsequently, the images were stacked with maximum-intensity projection using the software Zeiss Efficient Navigation (Zen) (Carl Zeiss MicroImaging GmbH).

### Nanoindentation and EDX

Data on hardness (*H*) and Young’s modulus (elastic modulus) (*E*) of the radular working zone was published previously by Gorb and Krings (2021), where six adult specimens of *Lavigeria grandis* were studied. For two of these specimens (150020/999–2, 150020/999–3), we previously also published on the elemental composition of the radular working zone by energy-dispersive X-ray spectroscopy (EDX) (Krings et al., 2022a, b). To determine if the platinum coating, needed to perform EDX analysis (please see below for protocol), had an influence on the mechanical properties, we performed Tukey–Kramer tests for pairwise comparisons on the tooth parts of the working zone (see Supplementary Table 1). We did not find differences between uncoated and coated sample for both *E* and *H* values, which showed that the coating did not influence the mechanical properties — at least in the material depths 460–520 nm, which was targeted in this and the previous studies on nanoindentation (please see below for protocol). Thus, we here add data on the chemical composition, the hardness and elasticity for the remaining ontogenetic stages (zones 1–3) using the same sample for both techniques. For this, radulae were extracted from specimens, freed from surrounding tissues, cleaned in an ultrasonic bath (lasting 5 s) and attached to glass object slides (Carl Roth, Karlsruhe, Germany) with double-sided adhesive tape along their longitudinal axis, following established protocols (Krings et al. 2022a, b, c; Brütt et al. 2022). After drying at room temperature, each radula was surrounded by a small metallic ring, which was filled with epoxy resin (Reckli Epoxi WST, Reckli GmbH, Herne, Germany), polymerizing for 3 days at room temperature (Young’s modulus of the polymerized epoxy is 1.3 ± 0.3 GPa). The metallic ring ensured an almost parallel sample. The specific epoxy was chosen, because it does not infiltrate the tooth material. After polymerization, object slide and adhesive tape were removed, and each sample was polished with sandpapers of distinct roughness until marginal teeth were on display. Then, the surface was smoothened on a polishing machine (Minitech 233/333, PRESI GmbH, Hagen, Germany) with aluminium oxide polishing powder suspension of 0.3 μm grain size (Presi GmbH, Hagen, Germany). Samples were cleaned in an ultrasonic bath for 5 min, dried and sputter-coated with platinum (5 nm layer).

The SEM Zeiss LEO 1525 (One Zeiss Drive, Thornwood, New York, USA) equipped with an Octane Silicon Drift Detector (SDD) (micro analyses system TEAM, EDAX Inc., New Jersey, USA) was employed for analysing the elemental composition of the embedded teeth (the largest area of each tooth possible; 100–200 μm^2^, depending on the tooth), always using an acceleration voltage of 20 keV and the same settings (e.g. lens opening, working distance). Before analysing a sample, the detector was always calibrated with copper. The presence of the following elements was detected and their proportions (atomic %) measured: H (hydrogen), C (carbon), N (nitrogen), O (oxygen), Pt (platinum), Al (aluminium), Ca (calcium), Na (sodium), Mg (magnesium), Si (silicon), P (phosphor), S (sulphur) and Cl (chlorine). We used the data of atomic ratio (atomic %) for this study. We did not analyse and discuss the following elements, because they are the elemental basis of either chitin (H, C, N, O), the coating (Pt) or of the polishing powder (Al, O). Ca, Na, Mg, Si, P, S and Cl were also summoned to “all elements” (Ae). First, marginal teeth were analysed by EDX; afterwards, nanoindentation was performed on the same samples.

For nanoindentation (for detailed protocol see Krings et al. 2019, 2021f; Gorb and Krings 2021), a nanoindenter SA2 (MTS Nano Instruments, Oak Ridge, Tennessee, USA) equipped with a Berkovich indenter tip and a dynamic contact module (DCM) head was employed. Hardness and Young’s modulus were determined from force-distance curves by applying the continuous stiffness mode (Oliver and Pharr 1992). All tests were performed under normal room conditions (relative humidity 28–30%, temperature 22–24 °C), and each indent and corresponding curve were manually controlled. Each marginal tooth was tested at three regions (basis, stylus, cusp). *E* and *H* of the tooth cusps were determined at the penetration depth of 480–520 nm and of the styli and the bases at 460–500 nm. For each site indented, we received 30 values, which were collated to receive one *H* and one *E* mean value per indent. After measuring the marginal teeth, the samples were polished until the lateral teeth were on display. Nanoindentation and EDX analyses were performed in the same manner as on the marginals. All steps of the protocol were repeated to receive data on the laterals, centrals, laterals and marginals from the other side. However, due to smallness of central and lateral teeth, these teeth were measured by nanoindentation at only two regions (stylus and cusp).

Water is known to be an important modulator of the mechanical properties in chitin; the employed nanoindentation technique however does not allow the testing of teeth under wet condition. We would propose that *L. grandis*’ teeth, similar to chiton teeth where a 15% reduction in *E* and *H* values was previously reported for wet state (Weaver et al. 2010), are softer and more flexible under native condition. Since gastropods were killed and fixed in 70% EtOH, the alcohol could have also altered the mechanical properties. As this fluid interacted however with every tooth part, we would propose that the material property gradients detected can still be accounted as valid.

Overall, 236 EDX (= 236 studied teeth) and 700 nanoindentation measurements were successfully performed.

### Statistical analyses

Statistical analyses were performed with JMP Pro, Version 14 (SAS Institute Inc., Cary, North Caroline, 1989–2007). Mean values and standard deviations were calculated, and Shapiro–Wilk-W-tests for testing of normality were conducted. Data on hardness and Young’s modulus were normally distributed. Here, ANOVA to detect differences and the Tukey–Kramer test for pairwise comparisons were performed. Data on elemental composition was non-normally distributed; here, a chi-squared test approximation and a Wilcoxon signed-rank test for pairwise comparison were carried out. All correlations were also computed with JMP Pro software.

## Results

### Morphology

The radula was taeniogloss, i.e. with seven teeth per row (Fig. 2b, see Krings et al. 2021e, f; Krings and Gorb 2021a for a detailed description of *Lavigeria grandis* radular morphology). Each central tooth (CT) and lateral tooth (LT) possessed a broad cusp and a short stylus; the laterals were however longer and had a broader basis than the centrals. The marginal teeth (MT) were rather long, slender and bear spoon-like cusps, which could embrace one another. In most cases, the marginal I cusps from two adjacent tooth rows, as well as the marginal II cusps from two adjacent rows, interacted with each other.

### Autofluorescence

**Fig. 3:**
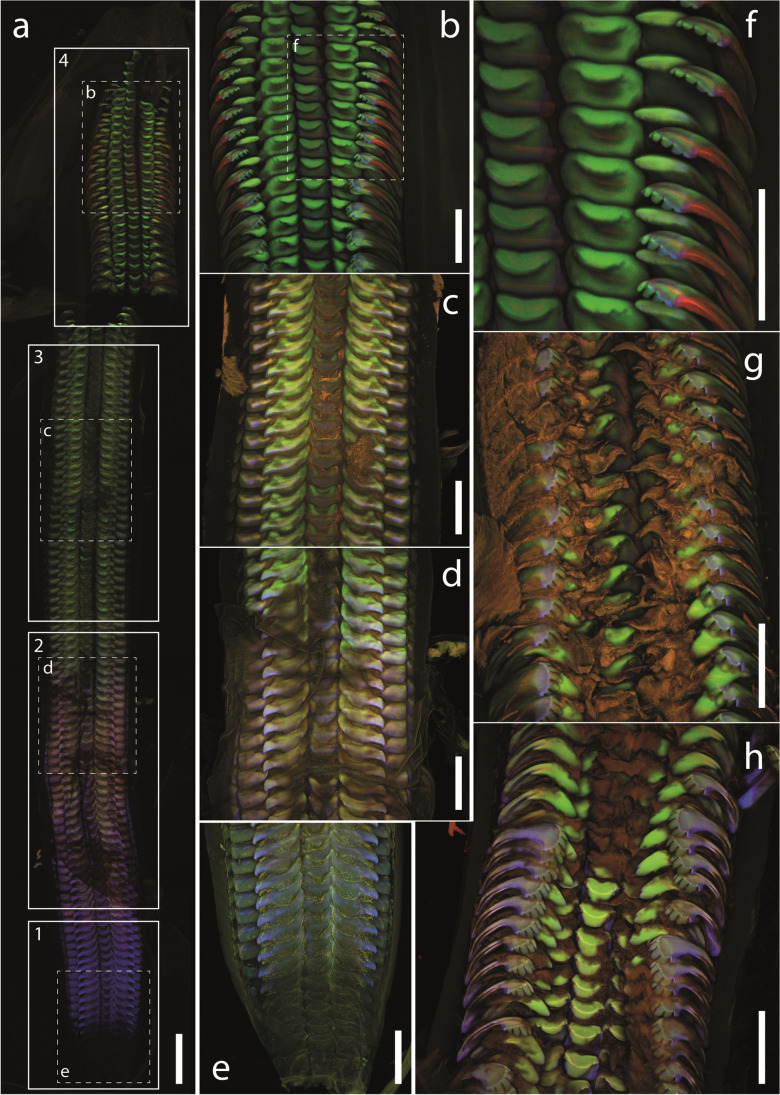
CLSM images of two radulae (specimen 1: **a**–**f**, specimen 2: **g**–**h**). **a** One image of whole radula with highlighted ontogenetic zones 1–4. The colours of the zones can be directly compared. Regions, which were also documented with a higher magnification, are highlighted. **b**–**f** Images of the working zone (**b**, **f**), zone 3 (**c**, **g**), zone 2 (**d**, **h**) and finally the radular sac (**e**). Comparison of colours is only possible within each individual image, as they were taken individually. Scale bars: **a** 0.5 mm; **b**–**h** 250 µm

When the whole radula is documented with one single scan, we could detect the general ontogenetic changes in autofluorescence signal (Fig. 3a). All structures (teeth and membrane) of the radular sac (zone 1) appeared blue. In zone 2, we detected a gradual change from blue to purple, across brownish, yellow, until teeth were finally green. In zone 3, most teeth were green, and in zone 4 (working zone), some regions appeared red.

**Fig. 4:**
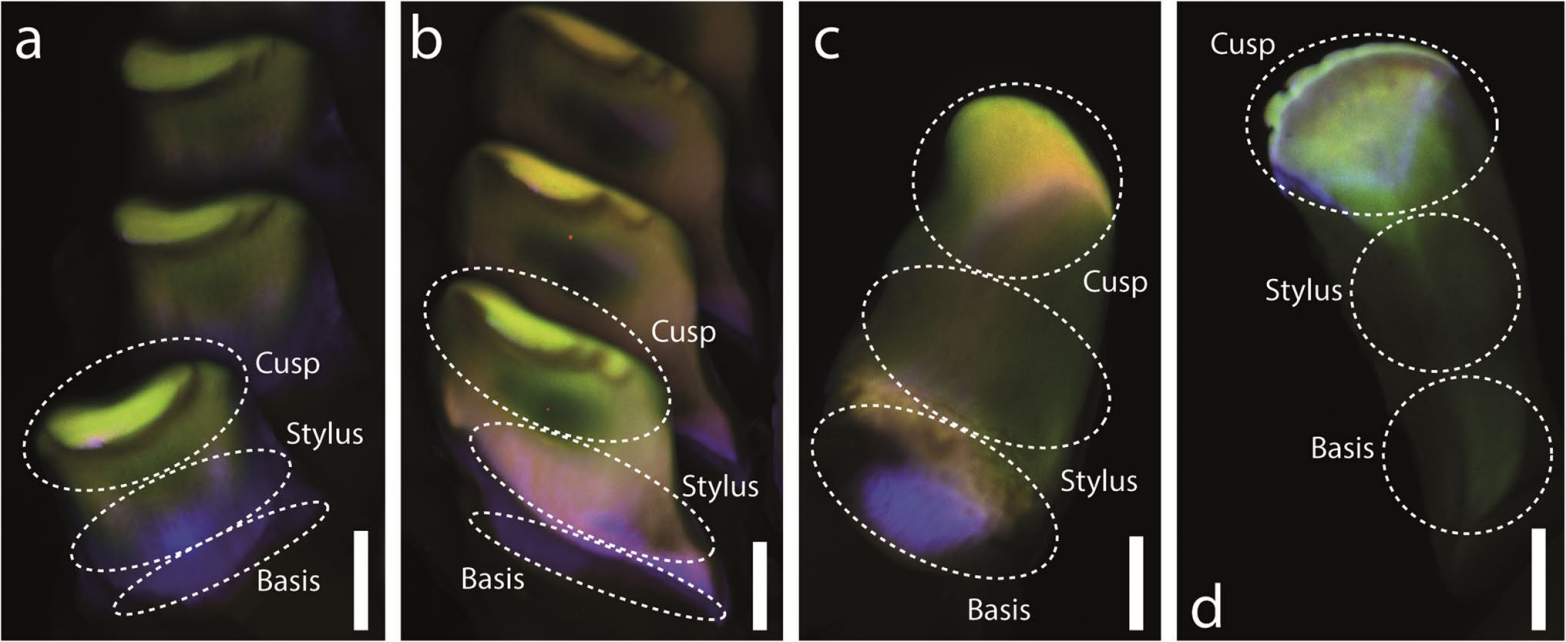
CLSM images of extracted mature teeth from the radular working zone (specimen 3). Within each image, the colours of the tooth regions can be directly compared. Distinct regions (cusp, stylus, basis), which were tested by nanoindentation, are highlighted and labelled. **a** Central teeth. **b** Lateral teeth. **c** One marginal tooth I (inner marginal tooth). **d** One marginal tooth II (outer marginal tooth). Scale bars: **a**–**d** 50 µm

When each zone was documented individually (Fig. 3b–e), we could determine differences between the tooth types and within teeth, but we could not compare between the zones as each picture stands for itself. In the radular sac (Fig. 3e), the membrane and teeth showed a similar signal (green); only the bases of the teeth were rather bluish. In zones 2 and 3 (Fig. 3c and d), blue was detected in the younger teeth (Fig. 3h), but then changed to a rather greenish and finally orange colour. The blue signal persisted only in the tooth bases and the cusps of the marginal teeth (Fig. 3g). In the working zone, red was detected on the outer edges of the marginal teeth I and II (Fig. 3d and f). When the individual teeth were extracted from the working zone, the colour of the tooth regions could be identified (Fig. 4). All tooth bases were blue, whereas the cusps were rather brown to green. In both marginal teeth, we also determined a blue region on the tooth cusps (Fig. 4c and d) and a red strand along the lateral edge of each marginal (Fig. 3b, f).

### Mechanical properties

Hardness and Young’s modulus were highly correlated (*r* = 0.8125; *p* < 0.0001). Hardness and Young’s modulus differed significantly between (a) all zones and (b) most regions (cusp, styli, bases) of the distinct tooth types (central, lateral, marginal I, marginal II) from the distinct zones (1–4) (see Supplementary Table 1).

**Fig. 5:**
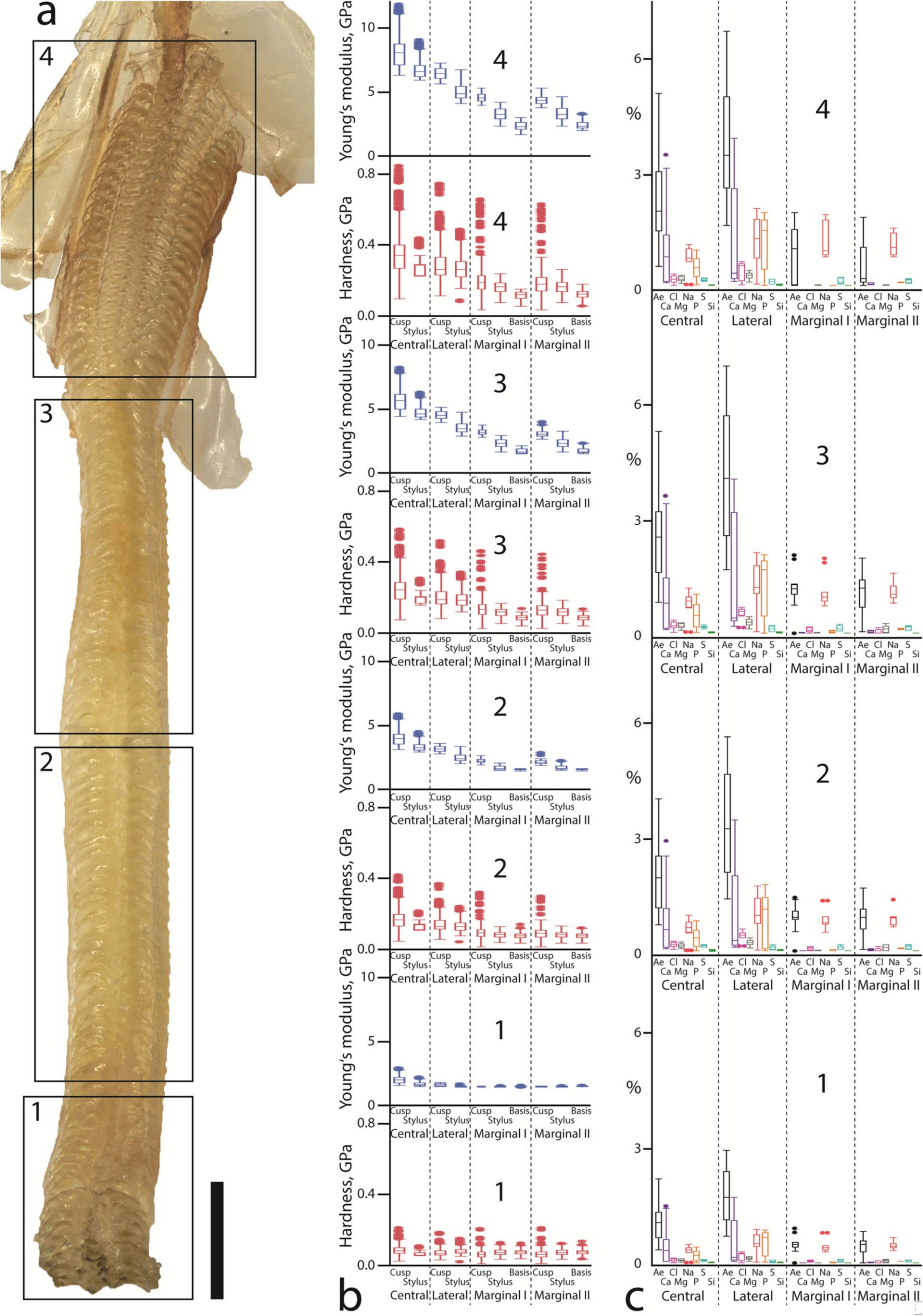
**a** Radula (specimen 4) with alary processes, situated lateral to the working zone, is documented by light microscopy. The radular zones 1–4 are highlighted and labelled. **b** The results from nanoindentation on the hardness (*H*) and then Young’s modulus (*E*), both given in GPa, of the distinct tooth regions from the different ontogenetic zones (1–4) are displayed (for values see Table 1). **c** The results from elemental analysis by EDX of the distinct tooth types from the different ontogenetic zones (1–4) are shown (for values see Table 1). The elemental proportions, given in atomic %, of all elements (Ae), calcium (Ca), chloride (Cl), magnesium (Mg), sodium (Na), phosphorus (P), sulphur (S) and silicon (Si) are presented. Scale bar: **a** 500 µm

**Fig. 6:**
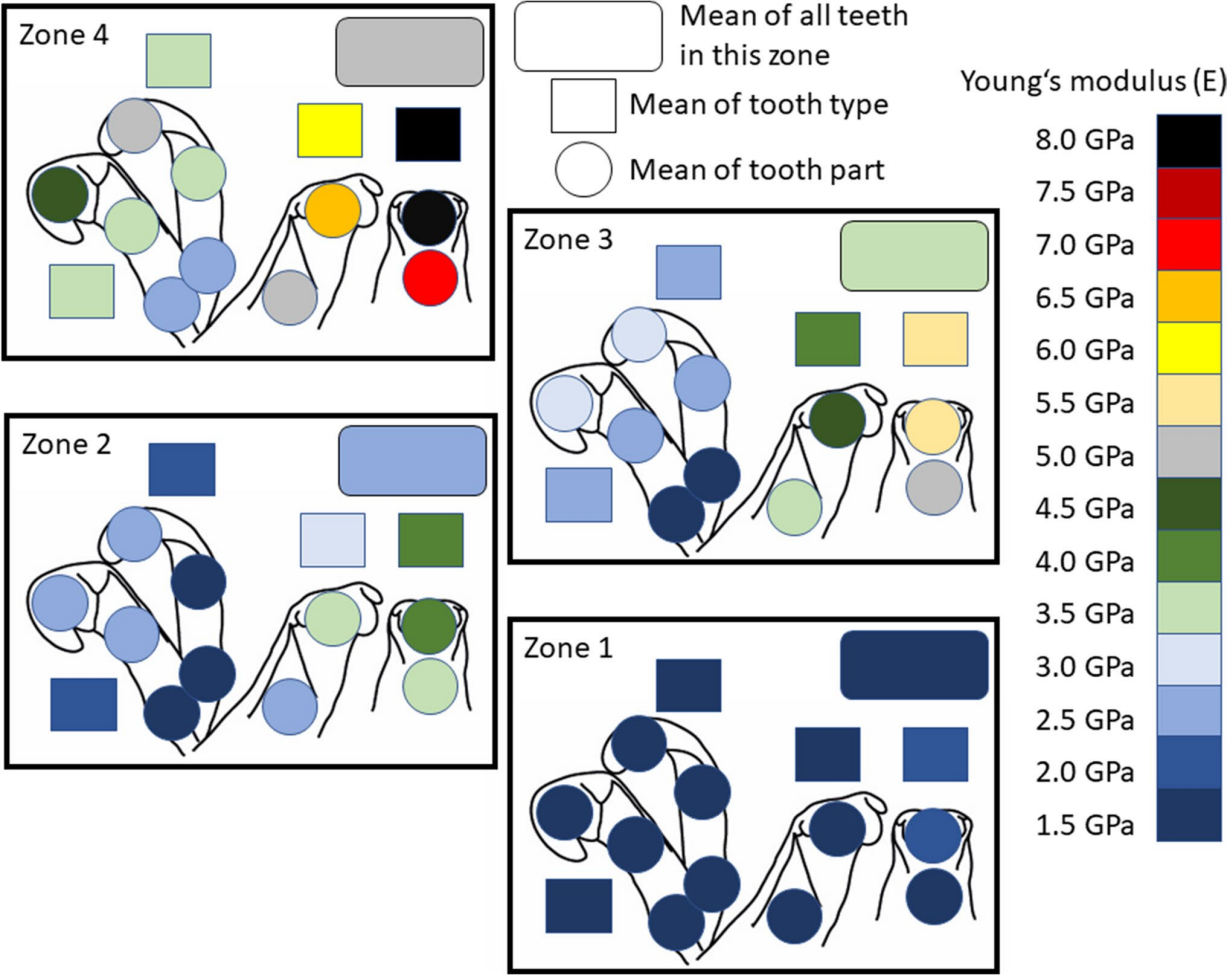
Results from nanoindentation; mean of Young’s modulus (*E*) determined for the tooth parts, tooth types in each zone (1–4) displaying the gradients within each tooth, in each row and through radular ontogeny

The radular sac (zone 1) contained the softest and most flexible teeth, followed by zones 2, 3 and finally the working zone (zone 4) with the hardest and stiffest ones (see Table 1; Figs. 5 and 6). In all zones, the centrals were the hardest and stiffest teeth, followed by the laterals, marginal I and finally marginal II as the softest and most flexible teeth. In each zone, central tooth cusps were the hardest and stiffest regions, followed by their styli and the lateral tooth cusps. In zone 1, this sequence was continued with the marginal tooth basis, lateral tooth stylus and marginal stylus and finally marginal tooth cusp as the softest and most flexible region. From zones 2 to 4, lateral cusps were always followed by the lateral styli. In zone 2, this sequence was continued by the marginal I cusps, marginal II cusps, marginal II styli, marginal I styli, marginal I bases and finally marginal II bases as the softest and most flexible regions. In zone 3, they were followed by the marginal I cusps, marginal II cusps, marginal II styli, marginal I styli, marginal II bases and finally marginal I bases as the softest and most flexible regions. In the working zone (zone 4), the sequence was continued as follows: marginal I cusps, marginal II cusps, marginal II styli, marginal I styli, marginal II bases and finally marginal I bases (see Table 1; Figs. 5 and 6).

**Table 1:**
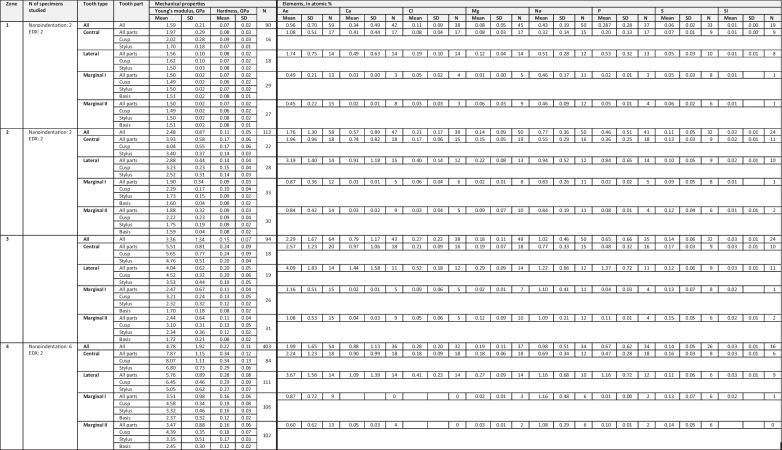
The measured mechanical properties hardness and Young’s modulus, both given in GPa, and the proportions of the individual elements, given in atomic %, for each zone and tooth type are listed. *Ae* all elements together, *N* quantity of teeth tested

### Elemental composition

The highest content of all elements (see Table 1; Fig. 5) was detected in zone 3, followed by the working zone (zone 4), zone 2 and finally the radular sac (zone 1) with the smallest mineral content. From zone 1 across zones 2 to zone 3, we determined a steady increase for each element analysed. From zone 3 to the working zone, we detected an increase of Ca and Mg content and a decrease in proportions for Ae, Cl, Na and P; the S and Si content remained constant.

For such elements as Ca, Cl, Mg and P, the lateral tooth contained the highest proportions, followed by centrals, marginals I and finally the marginals II — in each zone. Na was detected mostly in laterals, followed by marginals and finally centrals — in each zone. S was abundant mostly in centrals, followed by the marginals and finally laterals — in zones 1, 3 and 4. Si was detected in very small proportions and was found to be evenly distributed in all teeth and all zones.

Proportions of the individual elements correlated in most cases very slightly (Si with each individual other elements), slightly (e.g. Ca and S) or moderately (e.g. Cl and Na). For some elements, we detected very high correlations (Ca with Cl or P). In most cases, elemental content differed significantly between (a) zones and (b) the tooth types (central, lateral, marginal I, marginal II) from the distinct zones (1–4) (see Supplementary Table 2).

### Relationship between mechanical and chemical parameters

Young’s modulus and hardness related to Ae, Ca, Cl, Mg, P and S moderately, and to Na and Si with low and very low correlation coefficients (see Supplementary Table 3).

## Discussion

### CLSM as tool to determine functional gradients in chitinous structures

For radulae, autofluorescence signals were previously documented to discuss microscopy methodologies in online forums and on webpages (e.g. *microscopy-uk.org.uk*, *moticeurope.blogspot.com*) or to emphasize the advantages of integrating autofluorescence images in studies as specimens are of high contrast between radulae and the matrix surrounding (Haug et al. 2011). Additionally, radulae were previously stained and then documented using CLSM, to determine actin filaments, nuclei and chitin (Mikhlina et al. 2018; Vortsepneva and Tzetlin 2019; Vortsepneva 2020; Vortsepneva et al. 2021a, b).

The arthropod cuticle similar to the radula is a chitinous composite material of various components with different material properties, including stiff and hard or soft and flexible regions. Laser excitation by CLSM allowed previously the identification of cuticula regions with certain dominating material composition. Michels and Gorb (2012) related the material properties to the autofluorescence signals as the following: (a) sclerotized, stiff cuticle to a red signal and (b) weakly sclerotized chitin to a green signal. These latter parts were flexible and relatively tough. When resilin, an elastic and flexible protein (see e.g. Weis-Fogh 1960; Andersen and Weis-Fogh 1964; Burrows 2009; for review see Michels et al. 2016), was abundant, those structures appear brown, yellow or pink in overlay, as resilin produced a blue signal. (c) Blue signals in cuticula can come from regions containing high proportions of resilin; however, several other proteins showed a similar autofluorescence (e.g. Garcia-Castineiras et al. 1978; Fujimori 1978; Gast and Lee 1978; Giurginca et al. 2015). This protocol (Michels and Gorb 2012) was applied and enabled to find functional significances of material properties in many studies on arthropod endocuticle (Wang et al. 2019), wings (Rajabi et al. 2018; Ma et al. 2022), foot attachment devices and legs (Peisker et al. 2013; Rebora et al. 2018; Petersen et al. 2018; Friedrich and Kubiak 2018; Jandausch et al. 2018; Beutel et al. 2020; Büsse et al. 2021; Li et al. 2021; Büscher et al. 2021), thorax structure (Casey et al. 2022), antennae (Saltin et al. 2019), mouthparts (Michels et al. 2012; Michels and Gorb 2015; Büsse and Gorb 2018; Weihmann and Wipfler 2019; Matsumura et al. 2021a, b; Lehnert et al. 2021; Sun et al. 2021; Wei et al. 2022) or genitals (Matsumura et al. 2014, 2017, 2020a, b, 2021a, b; Kamimura et al. 2021). In addition, this protocol was cross-validated by employing AFM-Nanoindentation at least for hair of foot attachment devices in a lady beetle (Peisker et al. 2013).

For radulae studies, nanoindentation have been successfully applied to characterise material properties (Krings et al. 2019, 2021b, c, d, e, f, 2022c; Gorb and Krings 2021); however, there are some limitations with this technique as discussed below (“Nanoindentation and CLSM complement each other”). By combining CLSM, nanoindentation and EDX, we found CLSM as an additional tool to discuss functional gradients or material properties of radular structures for the first time.

### Origins of functional gradients in radular teeth

For *Lavigeria grandis*, we were here able to relate the mechanical properties, tested by nanoindentation, with the autofluorescence signals. For example, in mature teeth, tooth bases and styli were softer and more flexible and showed a blue signal, whereas the cusps were harder and stiffer and exhibited a green signal. Additionally, structures of the radular sac appeared blue, then turned purple, brown and yellowish, before they appeared green and finally red in the working zone — these transitions went along with an increase in hardness and Young’s modulus. Regarding the chemical composition, we found that harder and stiffer teeth (centrals and laterals) contained more calcium than the softer and more flexible teeth (marginals); however, the tooth materials differed in only very few percentages. We thus concluded that the mechanical property gradients mainly have their origin in the degree of tanning and the abundance of proteins, similar to the situation in insect cuticula. We propose that the blue signal in radula was probably an indication for the presence of high proportions of proteins rich on amino acids with aromatic rings, which increased the flexibility and softness of teeth. These proteins could potentially also include resilin, which was previously also detected outside the arthropod realm, i.e. in Plathelminthes (Wong et al. 2013; Wong and Gorb 2013), but it could also be a protein similar to resilin, which was previously detected in mussels (DeVore and Gruebel 1978). This should be tested by CLSM spectral analysis in the future. The green signal in *L. grandis*’ teeth was, as in cuticula, probably related to weakly sclerotised chitin-rich parts and the red signal to sclerotised (tanned) material.

### Nanoindentation and CLSM complement each other

The mechanical property gradients along the mature teeth and their functional significance were previously discussed for *Lavigeria grandis* (Gorb and Krings 2021; Krings et al. 2021c, e). Using nanoindentation, we determined that the softer and flexible tooth bases and styli allowed the reliance of teeth onto teeth from the adjacent rows, whereas the harder and stiffer cusps interacted with the ingesta. Additionally, we hypothesised that the harder and stiffer centrals and laterals were probably used for loosening ingesta from the hard surfaces, whereas the softer and more flexible marginals gathered the particles. These hypotheses on functionality of radular teeth have been afterwards confirmed by breaking stress experiments, where we loaded wet teeth, observed their biomechanical behaviour and finally measured the force needed to break them (Krings et al. 2021c, d). Even though nanoindentation was found to be a very useful tool to build hypotheses on tooth function, our protocol holds sources of error: the teeth are rather small in comparison to the indenter tip, which only allows testing of larger tooth regions. Additionally, samples were polished to receive a plain surface for nanoindentation. As a consequence, longitudinal sections of the teeth, i.e. the inner tooth structure, were on display and not the outer layer on the tooth surface (Krings et al. 2022b). Finally, the teeth were embedded in epoxy, which means that only structures that were harder and stiffer than the epoxy itself could be tested with reliable values. Softer and more flexible structures would, however, have the hardness and Young’s modulus of the epoxy. This latest fact could also be observed in the here presented study, as none of the mechanical property values is smaller than the values measured for the epoxy. We can imagine that the teeth of zone 1 are probably less soft, with presumable values of the Young’s modulus in the range of MPa.

By including CLSM approach into our studies, we were able to address smaller-scaled heterogeneities in material and mechanical properties of the tooth regions. This led to a more comprehensive view on the biomechanics of *L. grandis*’ teeth: each marginal tooth possessed a cusp with a thin region producing a blue autofluorescence signal. This region could potentially serve as a shock absorber when interacting with a hard obstacle, similar to, e.g. copepod gnathobases (Michels and Gorb 2012), snake skin (Klein and Gorb 2012) or human teeth (Wang and Weiner 1998; Fong et al. 2000; Zheng et al. 2003). The red strands on the lateral edges of the marginal teeth, probably harder and stiffer, could potentially support a folding action after feeding. During foraging motion, the radula of *L. grandis* is probably first unfolded, a mechanism previously observed in gastropod species (Eigenbrodt 1941; Wägele 1983; Scheel et al. 2020) and during manipulation of physical radular models (Krings et al. 2021b). Here, the radular membrane was pulled by the radular surrounding structures, e.g. the alary processes on each side of the radula. This action stroked the marginals from the centrals and laterals, until their cusps were located laterally to the lateral tooth cusps (“distal flex”, see Krings et al. 2021b). As a next step, the centrals and laterals were probably forced onto the ingesta surface to loosen food particles by the gastropod. Directly after this very short interaction, the radula is retrained from the surface. Here, the stiffer and harder strands probably support the motion in only one direction and additionally a catapulting motion of the marginals onto the centrals and laterals (“proximal flex”, see Krings et al. 2021b). During this proximal flex action, the food particles are probably collected and transported into the oral cavity.

In general, we found that the here applied CLSM approach complements nanoindentation techniques as it adds important additional information on the material properties (see papers by Peisker et al. 2013; Eshghi et al. 2018; Schmitt et al. 2018, on the correlation between autofluorescence of insect cuticles and material properties). We hope that this technique will be used more frequently when studying radular functionality in the future.

## Supplementary Information

Below is the link to the electronic supplementary material.

Supplementary file1 (DOCX 290 KB)
