# Supplementary material for: Material gradients in gastropod radulae and their biomechanical significance: a combined approach on the paludomid Lavigeria grandis
Source: Naturwissenschaften. 2022 Nov 2;109(6):52. doi: 10.1007/s00114-022-01822-9 (PMC9630255; doi:10.1007/s00114-022-01822-9)
Supplement: Supplementary file 1 — Supplementary file1 (DOCX 290 KB) [file 114_2022_1822_MOESM1_ESM.docx]

**Material gradients in gastropod radulae and their biomechanical significance: a combined approach on the paludomid gastropod *Lavigeria* *grandis***

Wencke Krings^1,2,3*^, Yoko Matsumura^3,4^, Jan-Ole Brütt^1,2^, Stanislav N. Gorb^3^

^1^Department of Behavioral Biology, Institute of Cell and Systems Biology of Animals, Universität Hamburg, Martin-Luther-King-Platz 3, 20146 Hamburg, Germany

^2^Department of Mammalogy and Palaeoanthropology, Leibniz Institute for the Analysis of Biodiversity Change, Martin-Luther-King-Platz 3, 20146 Hamburg, Germany

^3^Department of Functional Morphology and Biomechanics, Zoologisches Institut, Christian-Albrechts-Universität zu Kiel, Am Botanischen Garten 9, 24118 Kiel, Germany

^4^Department of General and Systematic Zoology, Zoological Institute and Museum, Universität Greifswald, Loitzer Str. 26, 17489 Greifswald, Germany

*corresponding author: wencke.krings@uni-hamburg.de

**Supplementary material**

**Supplementary Table 1.** Pairwise comparisons for Young’s modulus (*E)* and hardness (H) between (a) the platinum-coated and uncoated samples (highlighted in grey) and (b) the zones and tooth types according to the Tukey-Kramer test (orange p-values = highly significant, red = significant, black = not significant).

| **Parameter** | **Structure 1** | **Structure 2** | **Results from ANOVA** | | | **Result from Tukey-Kramer** |
| --- | --- | --- | --- | --- | --- | --- |
|  |  |  | **F ratio** | **df** | **p-value** | **p-value** |
| H | Zone 4 Central Stylus not coated | Zone 4 Central Stylus coated |  |  |  | 1.0000 |
| H | Zone 4 Central Cusp not coated | Zone 4 Central Cusp coated |  |  |  | 1.0000 |
| H | Zone 4 Lateral Stylus not coated | Zone 4 Lateral Stylus coated |  |  |  | 0.9911 |
| H | Zone 4 Lateral Cusp not coated | Zone 4 Lateral Cusp coated |  |  |  | 0.9724 |
| H | Zone 4 Marginal I Basis not coated | Zone 4 Marginal I Basis coated |  |  |  | 0.9981 |
| H | Zone 4 Marginal I Stylus not coated | Zone 4 Marginal I Stylus coated |  |  |  | 0.9893 |
| H | Zone 4 Marginal I Cusp not coated | Zone 4 Marginal I Cusp coated |  |  |  | 1.0000 |
| H | Zone 4 Marginal II Basis not coated | Zone 4 Marginal II Basis coated |  |  |  | 0.9731 |
| H | Zone 4 Marginal II Stylus not coated | Zone 4 Marginal II Stylus coated |  |  |  | 0.9821 |
| H | Zone 4 Marginal II Cusp not coated | Zone 4 Marginal II Cusp coated |  |  |  | 1.0000 |
| E | Zone 4 Central Stylus not coated | Zone 4 Central Stylus coated |  |  |  | 0.9861 |
| E | Zone 4 Central Cusp not coated | Zone 4 Central Cusp coated |  |  |  | 0.9772 |
| E | Zone 4 Lateral Stylus not coated | Zone 4 Lateral Stylus coated |  |  |  | 0.9981 |
| E | Zone 4 Lateral Cusp not coated | Zone 4 Lateral Cusp coated |  |  |  | 0.9842 |
| E | Zone 4 Marginal I Basis not coated | Zone 4 Marginal I Basis coated |  |  |  | 0.9999 |
| E | Zone 4 Marginal I Stylus not coated | Zone 4 Marginal I Stylus coated |  |  |  | 0.9995 |
| E | Zone 4 Marginal I Cusp not coated | Zone 4 Marginal I Cusp coated |  |  |  | 1.0000 |
| E | Zone 4 Marginal II Basis not coated | Zone 4 Marginal II Basis coated |  |  |  | 1.0000 |
| E | Zone 4 Marginal II Stylus not coated | Zone 4 Marginal II Stylus coated |  |  |  | 1.0000 |
| E | Zone 4 Marginal II Cusp not coated | Zone 4 Marginal II Cusp coated |  |  |  | 1.0000 |
| E | Zone 4 Central Stylus not coated | Zone 4 Central Stylus coated |  |  |  | 0.9971 |
| E | Zone 4 Central Cusp not coated | Zone 4 Central Cusp coated |  |  |  | 0.9943 |
| E | Zone 4 Lateral Stylus not coated | Zone 4 Lateral Stylus coated |  |  |  | 0.9951 |
| E | Zone 4 Lateral Cusp not coated | Zone 4 Lateral Cusp coated |  |  |  | 0.9645 |
| H | Zone 4 | Zone 1 | 10109.82 | 3 | <.0001* | <.0001* |
| H | Zone 4 | Zone 2 |  |  |  | <.0001* |
| H | Zone 3 | Zone 1 |  |  |  | <.0001* |
| H | Zone 4 | Zone 3 |  |  |  | <.0001* |
| H | Zone 3 | Zone 2 |  |  |  | <.0001* |
| H | Zone 2 | Zone 1 |  |  |  | <.0001* |
| E | Zone 4 | Zone 1 | 14855.52 | 3 | <.0001* | <.0001* |
| E | Zone 4 | Zone 2 |  |  |  | <.0001* |
| E | Zone 3 | Zone 1 |  |  |  | <.0001* |
| E | Zone 4 | Zone 3 |  |  |  | <.0001* |
| E | Zone 2 | Zone 1 |  |  |  | <.0001* |
| E | Zone 3 | Zone 2 |  |  |  | <.0001* |
| H | Central Cusp Zone 4 | Marginal I Cusp Zone 1 | 3289.141 | 39 | <.0001* | <.0001* |
| H | Central Cusp Zone 4 | Marginal II Cusp Zone 1 |  |  |  | <.0001* |
| H | Central Cusp Zone 4 | Central Stylus Zone 1 |  |  |  | <.0001* |
| H | Central Cusp Zone 4 | Lateral Cusp Zone 1 |  |  |  | <.0001* |
| H | Central Cusp Zone 4 | Marginal I Stylus Zone 1 |  |  |  | <.0001* |
| H | Central Cusp Zone 4 | Marginal II Basis Zone 1 |  |  |  | <.0001* |
| H | Central Cusp Zone 4 | Marginal II Stylus Zone 1 |  |  |  | <.0001* |
| H | Central Cusp Zone 4 | Marginal I Basis Zone 1 |  |  |  | <.0001* |
| H | Central Cusp Zone 4 | Marginal II Basis Zone 2 |  |  |  | <.0001* |
| H | Central Cusp Zone 4 | Marginal I Basis Zone 2 |  |  |  | <.0001* |
| H | Central Cusp Zone 4 | Lateral Stylus Zone 1 |  |  |  | <.0001* |
| H | Central Cusp Zone 4 | Marginal I Basis Zone 3 |  |  |  | <.0001* |
| H | Central Cusp Zone 4 | Marginal II Basis Zone 3 |  |  |  | <.0001* |
| H | Central Cusp Zone 4 | Central Cusp Zone 1 |  |  |  | <.0001* |
| H | Central Cusp Zone 4 | Marginal II Stylus Zone 2 |  |  |  | <.0001* |
| H | Central Cusp Zone 4 | Marginal I Stylus Zone 2 |  |  |  | <.0001* |
| H | Central Cusp Zone 4 | Marginal II Cusp Zone 2 |  |  |  | <.0001* |
| H | Central Cusp Zone 4 | Marginal I Cusp Zone 2 |  |  |  | <.0001* |
| H | Central Cusp Zone 4 | Marginal I Stylus Zone 3 |  |  |  | <.0001* |
| H | Central Cusp Zone 4 | Marginal II Stylus Zone 3 |  |  |  | <.0001* |
| H | Central Cusp Zone 4 | Marginal I Basis Zone 4 |  |  |  | <.0001* |
| H | Lateral Cusp Zone 4 | Marginal I Cusp Zone 1 |  |  |  | <.0001* |
| H | Lateral Cusp Zone 4 | Marginal II Cusp Zone 1 |  |  |  | <.0001* |
| H | Central Stylus Zone 4 | Marginal I Cusp Zone 1 |  |  |  | <.0001* |
| H | Central Stylus Zone 4 | Marginal II Cusp Zone 1 |  |  |  | <.0001* |
| H | Central Cusp Zone 4 | Marginal II Basis Zone 4 |  |  |  | <.0001* |
| H | Lateral Cusp Zone 4 | Central Stylus Zone 1 |  |  |  | <.0001* |
| H | Central Stylus Zone 4 | Central Stylus Zone 1 |  |  |  | <.0001* |
| H | Lateral Cusp Zone 4 | Lateral Cusp Zone 1 |  |  |  | <.0001* |
| H | Central Cusp Zone 4 | Marginal II Cusp Zone 3 |  |  |  | <.0001* |
| H | Central Stylus Zone 4 | Lateral Cusp Zone 1 |  |  |  | <.0001* |
| H | Lateral Cusp Zone 4 | Marginal I Stylus Zone 1 |  |  |  | <.0001* |
| H | Lateral Cusp Zone 4 | Marginal II Basis Zone 1 |  |  |  | <.0001* |
| H | Lateral Cusp Zone 4 | Marginal II Stylus Zone 1 |  |  |  | <.0001* |
| H | Lateral Cusp Zone 4 | Marginal I Basis Zone 1 |  |  |  | <.0001* |
| H | Central Stylus Zone 4 | Marginal I Stylus Zone 1 |  |  |  | <.0001* |
| H | Central Stylus Zone 4 | Marginal II Basis Zone 1 |  |  |  | <.0001* |
| H | Central Stylus Zone 4 | Marginal II Stylus Zone 1 |  |  |  | <.0001* |
| H | Central Stylus Zone 4 | Marginal I Basis Zone 1 |  |  |  | <.0001* |
| H | Central Cusp Zone 4 | Marginal I Cusp Zone 3 |  |  |  | <.0001* |
| H | Lateral Cusp Zone 4 | Marginal II Basis Zone 2 |  |  |  | <.0001* |
| H | Lateral Cusp Zone 4 | Marginal I Basis Zone 2 |  |  |  | <.0001* |
| H | Central Cusp Zone 4 | Lateral Stylus Zone 2 |  |  |  | <.0001* |
| H | Lateral Stylus Zone 4 | Marginal I Cusp Zone 1 |  |  |  | <.0001* |
| H | Lateral Stylus Zone 4 | Marginal II Cusp Zone 1 |  |  |  | <.0001* |
| H | Lateral Cusp Zone 4 | Lateral Stylus Zone 1 |  |  |  | <.0001* |
| H | Central Stylus Zone 4 | Marginal II Basis Zone 2 |  |  |  | <.0001* |
| H | Central Stylus Zone 4 | Marginal I Basis Zone 2 |  |  |  | <.0001* |
| H | Central Stylus Zone 4 | Lateral Stylus Zone 1 |  |  |  | <.0001* |
| H | Lateral Cusp Zone 4 | Marginal I Basis Zone 3 |  |  |  | <.0001* |
| H | Lateral Cusp Zone 4 | Marginal II Basis Zone 3 |  |  |  | <.0001* |
| H | Lateral Cusp Zone 4 | Central Cusp Zone 1 |  |  |  | <.0001* |
| H | Central Stylus Zone 4 | Marginal I Basis Zone 3 |  |  |  | <.0001* |
| H | Lateral Cusp Zone 4 | Marginal II Stylus Zone 2 |  |  |  | <.0001* |
| H | Lateral Cusp Zone 4 | Marginal I Stylus Zone 2 |  |  |  | <.0001* |
| H | Central Stylus Zone 4 | Marginal II Basis Zone 3 |  |  |  | <.0001* |
| H | Central Stylus Zone 4 | Central Cusp Zone 1 |  |  |  | <.0001* |
| H | Central Stylus Zone 4 | Marginal II Stylus Zone 2 |  |  |  | <.0001* |
| H | Central Stylus Zone 4 | Marginal I Stylus Zone 2 |  |  |  | <.0001* |
| H | Lateral Stylus Zone 4 | Central Stylus Zone 1 |  |  |  | <.0001* |
| H | Central Cusp Zone 4 | Central Stylus Zone 2 |  |  |  | <.0001* |
| H | Central Cusp Zone 4 | Lateral Cusp Zone 2 |  |  |  | <.0001* |
| H | Lateral Stylus Zone 4 | Lateral Cusp Zone 1 |  |  |  | <.0001* |
| H | Lateral Stylus Zone 4 | Marginal I Stylus Zone 1 |  |  |  | <.0001* |
| H | Lateral Cusp Zone 4 | Marginal II Cusp Zone 2 |  |  |  | <.0001* |
| H | Lateral Stylus Zone 4 | Marginal II Basis Zone 1 |  |  |  | <.0001* |
| H | Lateral Stylus Zone 4 | Marginal II Stylus Zone 1 |  |  |  | <.0001* |
| H | Lateral Stylus Zone 4 | Marginal I Basis Zone 1 |  |  |  | <.0001* |
| H | Central Stylus Zone 4 | Marginal II Cusp Zone 2 |  |  |  | <.0001* |
| H | Lateral Cusp Zone 4 | Marginal I Cusp Zone 2 |  |  |  | <.0001* |
| H | Central Stylus Zone 4 | Marginal I Cusp Zone 2 |  |  |  | <.0001* |
| H | Lateral Stylus Zone 4 | Marginal II Basis Zone 2 |  |  |  | <.0001* |
| H | Lateral Stylus Zone 4 | Marginal I Basis Zone 2 |  |  |  | <.0001* |
| H | Lateral Stylus Zone 4 | Lateral Stylus Zone 1 |  |  |  | <.0001* |
| H | Lateral Stylus Zone 4 | Marginal I Basis Zone 3 |  |  |  | <.0001* |
| H | Lateral Stylus Zone 4 | Marginal II Basis Zone 3 |  |  |  | <.0001* |
| H | Lateral Stylus Zone 4 | Central Cusp Zone 1 |  |  |  | <.0001* |
| H | Lateral Stylus Zone 4 | Marginal II Stylus Zone 2 |  |  |  | <.0001* |
| H | Lateral Stylus Zone 4 | Marginal I Stylus Zone 2 |  |  |  | <.0001* |
| H | Central Cusp Zone 3 | Marginal I Cusp Zone 1 |  |  |  | <.0001* |
| H | Central Cusp Zone 3 | Marginal II Cusp Zone 1 |  |  |  | <.0001* |
| H | Central Cusp Zone 4 | Marginal I Stylus Zone 4 |  |  |  | <.0001* |
| H | Central Cusp Zone 4 | Marginal II Stylus Zone 4 |  |  |  | <.0001* |
| H | Lateral Stylus Zone 4 | Marginal II Cusp Zone 2 |  |  |  | <.0001* |
| H | Lateral Stylus Zone 4 | Marginal I Cusp Zone 2 |  |  |  | <.0001* |
| H | Lateral Cusp Zone 4 | Marginal I Stylus Zone 3 |  |  |  | <.0001* |
| H | Central Cusp Zone 4 | Central Cusp Zone 2 |  |  |  | <.0001* |
| H | Central Cusp Zone 3 | Central Stylus Zone 1 |  |  |  | <.0001* |
| H | Lateral Cusp Zone 4 | Marginal II Stylus Zone 3 |  |  |  | <.0001* |
| H | Lateral Cusp Zone 4 | Marginal I Basis Zone 4 |  |  |  | <.0001* |
| H | Central Stylus Zone 4 | Marginal I Stylus Zone 3 |  |  |  | <.0001* |
| H | Central Cusp Zone 3 | Lateral Cusp Zone 1 |  |  |  | <.0001* |
| H | Central Stylus Zone 4 | Marginal II Stylus Zone 3 |  |  |  | <.0001* |
| H | Central Stylus Zone 4 | Marginal I Basis Zone 4 |  |  |  | <.0001* |
| H | Central Cusp Zone 3 | Marginal I Stylus Zone 1 |  |  |  | <.0001* |
| H | Central Cusp Zone 3 | Marginal II Basis Zone 1 |  |  |  | <.0001* |
| H | Central Cusp Zone 3 | Marginal II Stylus Zone 1 |  |  |  | <.0001* |
| H | Central Cusp Zone 3 | Marginal I Basis Zone 1 |  |  |  | <.0001* |
| H | Lateral Cusp Zone 4 | Marginal II Basis Zone 4 |  |  |  | <.0001* |
| H | Central Stylus Zone 4 | Marginal II Basis Zone 4 |  |  |  | <.0001* |
| H | Central Cusp Zone 3 | Marginal II Basis Zone 2 |  |  |  | <.0001* |
| H | Central Cusp Zone 3 | Marginal I Basis Zone 2 |  |  |  | <.0001* |
| H | Central Cusp Zone 3 | Lateral Stylus Zone 1 |  |  |  | <.0001* |
| H | Central Cusp Zone 4 | Marginal II Cusp Zone 4 |  |  |  | <.0001* |
| H | Lateral Cusp Zone 4 | Marginal II Cusp Zone 3 |  |  |  | <.0001* |
| H | Central Cusp Zone 3 | Marginal I Basis Zone 3 |  |  |  | <.0001* |
| H | Central Stylus Zone 4 | Marginal II Cusp Zone 3 |  |  |  | <.0001* |
| H | Central Cusp Zone 3 | Marginal II Basis Zone 3 |  |  |  | <.0001* |
| H | Central Cusp Zone 3 | Central Cusp Zone 1 |  |  |  | <.0001* |
| H | Central Cusp Zone 3 | Marginal II Stylus Zone 2 |  |  |  | <.0001* |
| H | Central Cusp Zone 3 | Marginal I Stylus Zone 2 |  |  |  | <.0001* |
| H | Lateral Cusp Zone 4 | Marginal I Cusp Zone 3 |  |  |  | <.0001* |
| H | Lateral Stylus Zone 4 | Marginal I Stylus Zone 3 |  |  |  | <.0001* |
| H | Central Cusp Zone 4 | Lateral Stylus Zone 3 |  |  |  | <.0001* |
| H | Central Stylus Zone 4 | Marginal I Cusp Zone 3 |  |  |  | <.0001* |
| H | Lateral Stylus Zone 4 | Marginal II Stylus Zone 3 |  |  |  | <.0001* |
| H | Lateral Cusp Zone 4 | Lateral Stylus Zone 2 |  |  |  | <.0001* |
| H | Lateral Stylus Zone 4 | Marginal I Basis Zone 4 |  |  |  | <.0001* |
| H | Central Cusp Zone 4 | Marginal I Cusp Zone 4 |  |  |  | <.0001* |
| H | Central Stylus Zone 4 | Lateral Stylus Zone 2 |  |  |  | <.0001* |
| H | Central Cusp Zone 3 | Marginal II Cusp Zone 2 |  |  |  | <.0001* |
| H | Lateral Stylus Zone 4 | Marginal II Basis Zone 4 |  |  |  | <.0001* |
| H | Central Cusp Zone 3 | Marginal I Cusp Zone 2 |  |  |  | <.0001* |
| H | Lateral Cusp Zone 4 | Central Stylus Zone 2 |  |  |  | <.0001* |
| H | Lateral Cusp Zone 4 | Lateral Cusp Zone 2 |  |  |  | <.0001* |
| H | Central Stylus Zone 4 | Central Stylus Zone 2 |  |  |  | <.0001* |
| H | Lateral Stylus Zone 4 | Marginal II Cusp Zone 3 |  |  |  | <.0001* |
| H | Central Stylus Zone 4 | Lateral Cusp Zone 2 |  |  |  | <.0001* |
| H | Central Cusp Zone 4 | Lateral Cusp Zone 3 |  |  |  | <.0001* |
| H | Central Cusp Zone 4 | Central Stylus Zone 3 |  |  |  | <.0001* |
| H | Central Stylus Zone 3 | Marginal I Cusp Zone 1 |  |  |  | <.0001* |
| H | Central Stylus Zone 3 | Marginal II Cusp Zone 1 |  |  |  | <.0001* |
| H | Lateral Cusp Zone 3 | Marginal I Cusp Zone 1 |  |  |  | <.0001* |
| H | Lateral Cusp Zone 3 | Marginal II Cusp Zone 1 |  |  |  | <.0001* |
| H | Lateral Stylus Zone 4 | Marginal I Cusp Zone 3 |  |  |  | <.0001* |
| H | Lateral Stylus Zone 4 | Lateral Stylus Zone 2 |  |  |  | <.0001* |
| H | Central Stylus Zone 3 | Central Stylus Zone 1 |  |  |  | <.0001* |
| H | Lateral Cusp Zone 3 | Central Stylus Zone 1 |  |  |  | <.0001* |
| H | Central Stylus Zone 3 | Lateral Cusp Zone 1 |  |  |  | <.0001* |
| H | Lateral Cusp Zone 3 | Lateral Cusp Zone 1 |  |  |  | <.0001* |
| H | Marginal I Cusp Zone 4 | Marginal I Cusp Zone 1 |  |  |  | <.0001* |
| H | Marginal I Cusp Zone 4 | Marginal II Cusp Zone 1 |  |  |  | <.0001* |
| H | Central Cusp Zone 3 | Marginal I Stylus Zone 3 |  |  |  | <.0001* |
| H | Central Stylus Zone 3 | Marginal I Stylus Zone 1 |  |  |  | <.0001* |
| H | Central Stylus Zone 3 | Marginal II Basis Zone 1 |  |  |  | <.0001* |
| H | Central Cusp Zone 3 | Marginal II Stylus Zone 3 |  |  |  | <.0001* |
| H | Central Stylus Zone 3 | Marginal II Stylus Zone 1 |  |  |  | <.0001* |
| H | Central Stylus Zone 3 | Marginal I Basis Zone 1 |  |  |  | <.0001* |
| H | Lateral Stylus Zone 3 | Marginal I Cusp Zone 1 |  |  |  | <.0001* |
| H | Lateral Stylus Zone 3 | Marginal II Cusp Zone 1 |  |  |  | <.0001* |
| H | Lateral Cusp Zone 3 | Marginal I Stylus Zone 1 |  |  |  | <.0001* |
| H | Central Cusp Zone 3 | Marginal I Basis Zone 4 |  |  |  | <.0001* |
| H | Lateral Stylus Zone 4 | Central Stylus Zone 2 |  |  |  | <.0001* |
| H | Lateral Stylus Zone 4 | Lateral Cusp Zone 2 |  |  |  | <.0001* |
| H | Lateral Cusp Zone 3 | Marginal II Basis Zone 1 |  |  |  | <.0001* |
| H | Lateral Cusp Zone 3 | Marginal II Stylus Zone 1 |  |  |  | <.0001* |
| H | Lateral Cusp Zone 3 | Marginal I Basis Zone 1 |  |  |  | <.0001* |
| H | Lateral Cusp Zone 4 | Marginal I Stylus Zone 4 |  |  |  | <.0001* |
| H | Lateral Cusp Zone 4 | Marginal II Stylus Zone 4 |  |  |  | <.0001* |
| H | Central Stylus Zone 4 | Marginal I Stylus Zone 4 |  |  |  | <.0001* |
| H | Central Stylus Zone 3 | Marginal II Basis Zone 2 |  |  |  | <.0001* |
| H | Central Stylus Zone 3 | Marginal I Basis Zone 2 |  |  |  | <.0001* |
| H | Central Cusp Zone 3 | Marginal II Basis Zone 4 |  |  |  | <.0001* |
| H | Central Stylus Zone 4 | Marginal II Stylus Zone 4 |  |  |  | <.0001* |
| H | Lateral Cusp Zone 3 | Marginal II Basis Zone 2 |  |  |  | <.0001* |
| H | Lateral Cusp Zone 3 | Marginal I Basis Zone 2 |  |  |  | <.0001* |
| H | Central Stylus Zone 3 | Lateral Stylus Zone 1 |  |  |  | <.0001* |
| H | Lateral Cusp Zone 3 | Lateral Stylus Zone 1 |  |  |  | <.0001* |
| H | Marginal II Cusp Zone 4 | Marginal I Cusp Zone 1 |  |  |  | <.0001* |
| H | Marginal II Cusp Zone 4 | Marginal II Cusp Zone 1 |  |  |  | <.0001* |
| H | Marginal I Cusp Zone 4 | Central Stylus Zone 1 |  |  |  | <.0001* |
| H | Central Stylus Zone 3 | Marginal I Basis Zone 3 |  |  |  | <.0001* |
| H | Lateral Stylus Zone 3 | Central Stylus Zone 1 |  |  |  | <.0001* |
| H | Lateral Cusp Zone 4 | Central Cusp Zone 2 |  |  |  | <.0001* |
| H | Marginal I Cusp Zone 4 | Lateral Cusp Zone 1 |  |  |  | <.0001* |
| H | Central Stylus Zone 3 | Marginal II Basis Zone 3 |  |  |  | <.0001* |
| H | Lateral Cusp Zone 3 | Marginal I Basis Zone 3 |  |  |  | <.0001* |
| H | Central Stylus Zone 3 | Central Cusp Zone 1 |  |  |  | <.0001* |
| H | Central Stylus Zone 3 | Marginal II Stylus Zone 2 |  |  |  | <.0001* |
| H | Lateral Cusp Zone 3 | Marginal II Basis Zone 3 |  |  |  | <.0001* |
| H | Central Stylus Zone 3 | Marginal I Stylus Zone 2 |  |  |  | <.0001* |
| H | Lateral Stylus Zone 3 | Lateral Cusp Zone 1 |  |  |  | <.0001* |
| H | Central Stylus Zone 4 | Central Cusp Zone 2 |  |  |  | <.0001* |
| H | Marginal I Cusp Zone 4 | Marginal I Stylus Zone 1 |  |  |  | <.0001* |
| H | Lateral Cusp Zone 3 | Central Cusp Zone 1 |  |  |  | <.0001* |
| H | Lateral Cusp Zone 3 | Marginal II Stylus Zone 2 |  |  |  | <.0001* |
| H | Lateral Cusp Zone 3 | Marginal I Stylus Zone 2 |  |  |  | <.0001* |
| H | Central Cusp Zone 3 | Marginal II Cusp Zone 3 |  |  |  | <.0001* |
| H | Marginal I Cusp Zone 4 | Marginal II Basis Zone 1 |  |  |  | <.0001* |
| H | Marginal I Cusp Zone 4 | Marginal II Stylus Zone 1 |  |  |  | <.0001* |
| H | Marginal I Cusp Zone 4 | Marginal I Basis Zone 1 |  |  |  | <.0001* |
| H | Lateral Stylus Zone 3 | Marginal I Stylus Zone 1 |  |  |  | <.0001* |
| H | Lateral Stylus Zone 3 | Marginal II Basis Zone 1 |  |  |  | <.0001* |
| H | Lateral Stylus Zone 3 | Marginal II Stylus Zone 1 |  |  |  | <.0001* |
| H | Lateral Stylus Zone 3 | Marginal I Basis Zone 1 |  |  |  | <.0001* |
| H | Marginal II Cusp Zone 4 | Central Stylus Zone 1 |  |  |  | <.0001* |
| H | Marginal I Cusp Zone 4 | Marginal II Basis Zone 2 |  |  |  | <.0001* |
| H | Marginal I Cusp Zone 4 | Marginal I Basis Zone 2 |  |  |  | <.0001* |
| H | Central Cusp Zone 3 | Marginal I Cusp Zone 3 |  |  |  | <.0001* |
| H | Marginal I Cusp Zone 4 | Lateral Stylus Zone 1 |  |  |  | <.0001* |
| H | Marginal II Cusp Zone 4 | Lateral Cusp Zone 1 |  |  |  | <.0001* |
| H | Lateral Stylus Zone 3 | Marginal II Basis Zone 2 |  |  |  | <.0001* |
| H | Central Stylus Zone 3 | Marginal II Cusp Zone 2 |  |  |  | <.0001* |
| H | Lateral Stylus Zone 3 | Marginal I Basis Zone 2 |  |  |  | <.0001* |
| H | Central Cusp Zone 3 | Lateral Stylus Zone 2 |  |  |  | <.0001* |
| H | Central Cusp Zone 2 | Marginal I Cusp Zone 1 |  |  |  | <.0001* |
| H | Central Cusp Zone 2 | Marginal II Cusp Zone 1 |  |  |  | <.0001* |
| H | Lateral Cusp Zone 3 | Marginal II Cusp Zone 2 |  |  |  | <.0001* |
| H | Lateral Stylus Zone 3 | Lateral Stylus Zone 1 |  |  |  | <.0001* |
| H | Marginal II Cusp Zone 4 | Marginal I Stylus Zone 1 |  |  |  | <.0001* |
| H | Marginal II Cusp Zone 4 | Marginal II Basis Zone 1 |  |  |  | <.0001* |
| H | Marginal II Cusp Zone 4 | Marginal II Stylus Zone 1 |  |  |  | <.0001* |
| H | Marginal II Cusp Zone 4 | Marginal I Basis Zone 1 |  |  |  | <.0001* |
| H | Marginal I Cusp Zone 4 | Marginal I Basis Zone 3 |  |  |  | <.0001* |
| H | Lateral Stylus Zone 4 | Marginal I Stylus Zone 4 |  |  |  | <.0001* |
| H | Lateral Cusp Zone 4 | Marginal II Cusp Zone 4 |  |  |  | <.0001* |
| H | Central Stylus Zone 3 | Marginal I Cusp Zone 2 |  |  |  | <.0001* |
| H | Marginal I Cusp Zone 4 | Marginal II Basis Zone 3 |  |  |  | <.0001* |
| H | Lateral Stylus Zone 4 | Marginal II Stylus Zone 4 |  |  |  | <.0001* |
| H | Lateral Cusp Zone 3 | Marginal I Cusp Zone 2 |  |  |  | <.0001* |
| H | Marginal I Cusp Zone 4 | Central Cusp Zone 1 |  |  |  | <.0001* |
| H | Lateral Stylus Zone 3 | Marginal I Basis Zone 3 |  |  |  | <.0001* |
| H | Marginal I Cusp Zone 4 | Marginal II Stylus Zone 2 |  |  |  | <.0001* |
| H | Central Stylus Zone 4 | Marginal II Cusp Zone 4 |  |  |  | <.0001* |
| H | Marginal I Cusp Zone 4 | Marginal I Stylus Zone 2 |  |  |  | <.0001* |
| H | Lateral Stylus Zone 3 | Marginal II Basis Zone 3 |  |  |  | <.0001* |
| H | Lateral Stylus Zone 3 | Central Cusp Zone 1 |  |  |  | <.0001* |
| H | Lateral Stylus Zone 3 | Marginal II Stylus Zone 2 |  |  |  | <.0001* |
| H | Lateral Stylus Zone 3 | Marginal I Stylus Zone 2 |  |  |  | <.0001* |
| H | Marginal II Stylus Zone 4 | Marginal I Cusp Zone 1 |  |  |  | <.0001* |
| H | Marginal II Stylus Zone 4 | Marginal II Cusp Zone 1 |  |  |  | <.0001* |
| H | Marginal II Cusp Zone 4 | Marginal II Basis Zone 2 |  |  |  | <.0001* |
| H | Marginal II Cusp Zone 4 | Marginal I Basis Zone 2 |  |  |  | <.0001* |
| H | Marginal I Stylus Zone 4 | Marginal I Cusp Zone 1 |  |  |  | <.0001* |
| H | Marginal I Stylus Zone 4 | Marginal II Cusp Zone 1 |  |  |  | <.0001* |
| H | Marginal II Cusp Zone 4 | Lateral Stylus Zone 1 |  |  |  | <.0001* |
| H | Central Cusp Zone 4 | Central Cusp Zone 3 |  |  |  | <.0001* |
| H | Lateral Stylus Zone 4 | Central Cusp Zone 2 |  |  |  | <.0001* |
| H | Central Cusp Zone 2 | Central Stylus Zone 1 |  |  |  | <.0001* |
| H | Lateral Cusp Zone 4 | Lateral Stylus Zone 3 |  |  |  | <.0001* |
| H | Central Cusp Zone 3 | Central Stylus Zone 2 |  |  |  | <.0001* |
| H | Central Cusp Zone 3 | Lateral Cusp Zone 2 |  |  |  | <.0001* |
| H | Marginal II Cusp Zone 4 | Marginal I Basis Zone 3 |  |  |  | <.0001* |
| H | Lateral Cusp Zone 4 | Marginal I Cusp Zone 4 |  |  |  | <.0001* |
| H | Central Cusp Zone 2 | Lateral Cusp Zone 1 |  |  |  | <.0001* |
| H | Central Stylus Zone 4 | Lateral Stylus Zone 3 |  |  |  | <.0001* |
| H | Marginal I Cusp Zone 4 | Marginal II Cusp Zone 2 |  |  |  | <.0001* |
| H | Marginal II Cusp Zone 4 | Marginal II Basis Zone 3 |  |  |  | <.0001* |
| H | Marginal II Cusp Zone 4 | Central Cusp Zone 1 |  |  |  | <.0001* |
| H | Marginal II Cusp Zone 4 | Marginal II Stylus Zone 2 |  |  |  | <.0001* |
| H | Central Stylus Zone 4 | Marginal I Cusp Zone 4 |  |  |  | <.0001* |
| H | Marginal II Cusp Zone 4 | Marginal I Stylus Zone 2 |  |  |  | <.0001* |
| H | Lateral Stylus Zone 3 | Marginal II Cusp Zone 2 |  |  |  | <.0001* |
| H | Central Cusp Zone 2 | Marginal I Stylus Zone 1 |  |  |  | <.0001* |
| H | Central Cusp Zone 2 | Marginal II Basis Zone 1 |  |  |  | <.0001* |
| H | Central Cusp Zone 2 | Marginal II Stylus Zone 1 |  |  |  | <.0001* |
| H | Central Cusp Zone 2 | Marginal I Basis Zone 1 |  |  |  | <.0001* |
| H | Marginal I Cusp Zone 4 | Marginal I Cusp Zone 2 |  |  |  | <.0001* |
| H | Marginal II Stylus Zone 4 | Central Stylus Zone 1 |  |  |  | <.0001* |
| H | Lateral Stylus Zone 3 | Marginal I Cusp Zone 2 |  |  |  | <.0001* |
| H | Marginal I Stylus Zone 4 | Central Stylus Zone 1 |  |  |  | <.0001* |
| H | Marginal II Stylus Zone 4 | Lateral Cusp Zone 1 |  |  |  | <.0001* |
| H | Marginal I Stylus Zone 4 | Lateral Cusp Zone 1 |  |  |  | <.0001* |
| H | Central Cusp Zone 2 | Marginal II Basis Zone 2 |  |  |  | <.0001* |
| H | Central Cusp Zone 2 | Marginal I Basis Zone 2 |  |  |  | <.0001* |
| H | Marginal II Stylus Zone 4 | Marginal I Stylus Zone 1 |  |  |  | <.0001* |
| H | Marginal II Stylus Zone 4 | Marginal II Basis Zone 1 |  |  |  | <.0001* |
| H | Marginal II Stylus Zone 4 | Marginal II Stylus Zone 1 |  |  |  | <.0001* |
| H | Marginal II Stylus Zone 4 | Marginal I Basis Zone 1 |  |  |  | <.0001* |
| H | Central Cusp Zone 2 | Lateral Stylus Zone 1 |  |  |  | <.0001* |
| H | Marginal I Stylus Zone 4 | Marginal I Stylus Zone 1 |  |  |  | <.0001* |
| H | Marginal II Cusp Zone 4 | Marginal II Cusp Zone 2 |  |  |  | <.0001* |
| H | Marginal I Stylus Zone 4 | Marginal II Basis Zone 1 |  |  |  | <.0001* |
| H | Marginal I Stylus Zone 4 | Marginal II Stylus Zone 1 |  |  |  | <.0001* |
| H | Marginal I Stylus Zone 4 | Marginal I Basis Zone 1 |  |  |  | <.0001* |
| H | Lateral Stylus Zone 4 | Marginal II Cusp Zone 4 |  |  |  | <.0001* |
| H | Lateral Cusp Zone 4 | Lateral Cusp Zone 3 |  |  |  | <.0001* |
| H | Central Cusp Zone 2 | Marginal I Basis Zone 3 |  |  |  | <.0001* |
| H | Central Stylus Zone 3 | Marginal I Stylus Zone 3 |  |  |  | <.0001* |
| H | Lateral Cusp Zone 4 | Central Stylus Zone 3 |  |  |  | <.0001* |
| H | Marginal II Cusp Zone 4 | Marginal I Cusp Zone 2 |  |  |  | <.0001* |
| H | Central Cusp Zone 2 | Marginal II Basis Zone 3 |  |  |  | <.0001* |
| H | Central Stylus Zone 3 | Marginal II Stylus Zone 3 |  |  |  | <.0001* |
| H | Central Stylus Zone 4 | Lateral Cusp Zone 3 |  |  |  | <.0001* |
| H | Lateral Cusp Zone 3 | Marginal I Stylus Zone 3 |  |  |  | <.0001* |
| H | Marginal II Stylus Zone 4 | Marginal II Basis Zone 2 |  |  |  | <.0001* |
| H | Marginal II Stylus Zone 4 | Marginal I Basis Zone 2 |  |  |  | <.0001* |
| H | Central Stylus Zone 3 | Marginal I Basis Zone 4 |  |  |  | <.0001* |
| H | Central Cusp Zone 2 | Central Cusp Zone 1 |  |  |  | <.0001* |
| H | Central Cusp Zone 2 | Marginal II Stylus Zone 2 |  |  |  | <.0001* |
| H | Central Cusp Zone 2 | Marginal I Stylus Zone 2 |  |  |  | <.0001* |
| H | Lateral Cusp Zone 3 | Marginal II Stylus Zone 3 |  |  |  | <.0001* |
| H | Central Stylus Zone 4 | Central Stylus Zone 3 |  |  |  | <.0001* |
| H | Lateral Cusp Zone 3 | Marginal I Basis Zone 4 |  |  |  | <.0001* |
| H | Marginal I Stylus Zone 4 | Marginal II Basis Zone 2 |  |  |  | <.0001* |
| H | Marginal II Stylus Zone 4 | Lateral Stylus Zone 1 |  |  |  | <.0001* |
| H | Marginal I Stylus Zone 4 | Marginal I Basis Zone 2 |  |  |  | <.0001* |
| H | Marginal I Stylus Zone 4 | Lateral Stylus Zone 1 |  |  |  | <.0001* |
| H | Lateral Cusp Zone 2 | Marginal I Cusp Zone 1 |  |  |  | <.0001* |
| H | Lateral Cusp Zone 2 | Marginal II Cusp Zone 1 |  |  |  | <.0001* |
| H | Central Stylus Zone 2 | Marginal I Cusp Zone 1 |  |  |  | <.0001* |
| H | Central Stylus Zone 2 | Marginal II Cusp Zone 1 |  |  |  | <.0001* |
| H | Lateral Stylus Zone 4 | Lateral Stylus Zone 3 |  |  |  | <.0001* |
| H | Marginal II Stylus Zone 4 | Marginal I Basis Zone 3 |  |  |  | <.0001* |
| H | Central Stylus Zone 3 | Marginal II Basis Zone 4 |  |  |  | <.0001* |
| H | Marginal II Stylus Zone 4 | Marginal II Basis Zone 3 |  |  |  | <.0001* |
| H | Lateral Cusp Zone 3 | Marginal II Basis Zone 4 |  |  |  | <.0001* |
| H | Marginal I Stylus Zone 4 | Marginal I Basis Zone 3 |  |  |  | <.0001* |
| H | Lateral Stylus Zone 4 | Marginal I Cusp Zone 4 |  |  |  | <.0001* |
| H | Marginal II Stylus Zone 4 | Central Cusp Zone 1 |  |  |  | <.0001* |
| H | Marginal II Stylus Zone 4 | Marginal II Stylus Zone 2 |  |  |  | <.0001* |
| H | Marginal II Stylus Zone 4 | Marginal I Stylus Zone 2 |  |  |  | <.0001* |
| H | Central Cusp Zone 3 | Marginal I Stylus Zone 4 |  |  |  | <.0001* |
| H | Marginal I Stylus Zone 4 | Marginal II Basis Zone 3 |  |  |  | <.0001* |
| H | Marginal I Stylus Zone 4 | Central Cusp Zone 1 |  |  |  | <.0001* |
| H | Marginal I Stylus Zone 4 | Marginal II Stylus Zone 2 |  |  |  | <.0001* |
| H | Marginal I Stylus Zone 4 | Marginal I Stylus Zone 2 |  |  |  | <.0001* |
| H | Central Cusp Zone 3 | Marginal II Stylus Zone 4 |  |  |  | <.0001* |
| H | Central Cusp Zone 2 | Marginal II Cusp Zone 2 |  |  |  | <.0001* |
| H | Marginal I Cusp Zone 4 | Marginal I Stylus Zone 3 |  |  |  | <.0001* |
| H | Central Cusp Zone 2 | Marginal I Cusp Zone 2 |  |  |  | <.0001* |
| H | Central Stylus Zone 3 | Marginal II Cusp Zone 3 |  |  |  | <.0001* |
| H | Marginal I Cusp Zone 4 | Marginal II Stylus Zone 3 |  |  |  | <.0001* |
| H | Marginal I Cusp Zone 4 | Marginal I Basis Zone 4 |  |  |  | <.0001* |
| H | Lateral Cusp Zone 2 | Central Stylus Zone 1 |  |  |  | <.0001* |
| H | Lateral Stylus Zone 3 | Marginal I Stylus Zone 3 |  |  |  | <.0001* |
| H | Lateral Cusp Zone 3 | Marginal II Cusp Zone 3 |  |  |  | <.0001* |
| H | Central Stylus Zone 2 | Central Stylus Zone 1 |  |  |  | <.0001* |
| H | Central Cusp Zone 4 | Lateral Stylus Zone 4 |  |  |  | <.0001* |
| H | Central Cusp Zone 3 | Central Cusp Zone 2 |  |  |  | <.0001* |
| H | Lateral Stylus Zone 3 | Marginal II Stylus Zone 3 |  |  |  | <.0001* |
| H | Lateral Stylus Zone 2 | Marginal I Cusp Zone 1 |  |  |  | <.0001* |
| H | Lateral Stylus Zone 2 | Marginal II Cusp Zone 1 |  |  |  | <.0001* |
| H | Marginal II Stylus Zone 4 | Marginal II Cusp Zone 2 |  |  |  | <.0001* |
| H | Lateral Stylus Zone 3 | Marginal I Basis Zone 4 |  |  |  | <.0001* |
| H | Lateral Cusp Zone 2 | Lateral Cusp Zone 1 |  |  |  | <.0001* |
| H | Central Stylus Zone 2 | Lateral Cusp Zone 1 |  |  |  | <.0001* |
| H | Marginal I Stylus Zone 4 | Marginal II Cusp Zone 2 |  |  |  | <.0001* |
| H | Marginal I Cusp Zone 3 | Marginal I Cusp Zone 1 |  |  |  | <.0001* |
| H | Marginal I Cusp Zone 3 | Marginal II Cusp Zone 1 |  |  |  | <.0001* |
| H | Lateral Cusp Zone 2 | Marginal I Stylus Zone 1 |  |  |  | <.0001* |
| H | Central Stylus Zone 3 | Marginal I Cusp Zone 3 |  |  |  | <.0001* |
| H | Central Stylus Zone 2 | Marginal I Stylus Zone 1 |  |  |  | <.0001* |
| H | Marginal I Cusp Zone 4 | Marginal II Basis Zone 4 |  |  |  | <.0001* |
| H | Lateral Stylus Zone 4 | Lateral Cusp Zone 3 |  |  |  | <.0001* |
| H | Marginal II Stylus Zone 4 | Marginal I Cusp Zone 2 |  |  |  | <.0001* |
| H | Lateral Cusp Zone 2 | Marginal II Basis Zone 1 |  |  |  | <.0001* |
| H | Lateral Cusp Zone 2 | Marginal II Stylus Zone 1 |  |  |  | <.0001* |
| H | Central Stylus Zone 2 | Marginal II Basis Zone 1 |  |  |  | <.0001* |
| H | Lateral Cusp Zone 2 | Marginal I Basis Zone 1 |  |  |  | <.0001* |
| H | Central Stylus Zone 2 | Marginal II Stylus Zone 1 |  |  |  | <.0001* |
| H | Central Stylus Zone 2 | Marginal I Basis Zone 1 |  |  |  | <.0001* |
| H | Lateral Cusp Zone 3 | Marginal I Cusp Zone 3 |  |  |  | <.0001* |
| H | Lateral Stylus Zone 4 | Central Stylus Zone 3 |  |  |  | <.0001* |
| H | Marginal I Stylus Zone 4 | Marginal I Cusp Zone 2 |  |  |  | <.0001* |
| H | Lateral Stylus Zone 3 | Marginal II Basis Zone 4 |  |  |  | <.0001* |
| H | Central Stylus Zone 3 | Lateral Stylus Zone 2 |  |  |  | <.0001* |
| H | Marginal II Cusp Zone 4 | Marginal I Stylus Zone 3 |  |  |  | <.0001* |
| H | Lateral Cusp Zone 3 | Lateral Stylus Zone 2 |  |  |  | <.0001* |
| H | Marginal II Cusp Zone 3 | Marginal I Cusp Zone 1 |  |  |  | <.0001* |
| H | Marginal II Cusp Zone 4 | Marginal II Stylus Zone 3 |  |  |  | <.0001* |
| H | Marginal II Cusp Zone 3 | Marginal II Cusp Zone 1 |  |  |  | <.0001* |
| H | Marginal II Cusp Zone 4 | Marginal I Basis Zone 4 |  |  |  | <.0001* |
| H | Lateral Cusp Zone 2 | Marginal II Basis Zone 2 |  |  |  | <.0001* |
| H | Lateral Cusp Zone 2 | Marginal I Basis Zone 2 |  |  |  | <.0001* |
| H | Central Stylus Zone 2 | Marginal II Basis Zone 2 |  |  |  | <.0001* |
| H | Central Stylus Zone 2 | Marginal I Basis Zone 2 |  |  |  | <.0001* |
| H | Lateral Stylus Zone 2 | Central Stylus Zone 1 |  |  |  | <.0001* |
| H | Lateral Cusp Zone 2 | Lateral Stylus Zone 1 |  |  |  | <.0001* |
| H | Central Stylus Zone 2 | Lateral Stylus Zone 1 |  |  |  | <.0001* |
| H | Marginal I Cusp Zone 4 | Marginal II Cusp Zone 3 |  |  |  | <.0001* |
| H | Lateral Stylus Zone 2 | Lateral Cusp Zone 1 |  |  |  | <.0001* |
| H | Marginal I Cusp Zone 3 | Central Stylus Zone 1 |  |  |  | <.0001* |
| H | Central Cusp Zone 3 | Marginal II Cusp Zone 4 |  |  |  | <.0001* |
| H | Lateral Stylus Zone 3 | Marginal II Cusp Zone 3 |  |  |  | <.0001* |
| H | Marginal II Cusp Zone 4 | Marginal II Basis Zone 4 |  |  |  | <.0001* |
| H | Lateral Cusp Zone 2 | Marginal I Basis Zone 3 |  |  |  | <.0001* |
| H | Central Stylus Zone 2 | Marginal I Basis Zone 3 |  |  |  | <.0001* |
| H | Lateral Stylus Zone 2 | Marginal I Stylus Zone 1 |  |  |  | <.0001* |
| H | Marginal I Cusp Zone 3 | Lateral Cusp Zone 1 |  |  |  | <.0001* |
| H | Lateral Cusp Zone 2 | Marginal II Basis Zone 3 |  |  |  | <.0001* |
| H | Central Stylus Zone 2 | Marginal II Basis Zone 3 |  |  |  | <.0001* |
| H | Lateral Stylus Zone 2 | Marginal II Basis Zone 1 |  |  |  | <.0001* |
| H | Lateral Stylus Zone 2 | Marginal II Stylus Zone 1 |  |  |  | <.0001* |
| H | Lateral Stylus Zone 2 | Marginal I Basis Zone 1 |  |  |  | <.0001* |
| H | Lateral Cusp Zone 2 | Central Cusp Zone 1 |  |  |  | <.0001* |
| H | Marginal II Basis Zone 4 | Marginal I Cusp Zone 1 |  |  |  | <.0001* |
| H | Marginal II Basis Zone 4 | Marginal II Cusp Zone 1 |  |  |  | <.0001* |
| H | Central Stylus Zone 2 | Central Cusp Zone 1 |  |  |  | <.0001* |
| H | Lateral Cusp Zone 2 | Marginal II Stylus Zone 2 |  |  |  | <.0001* |
| H | Lateral Cusp Zone 2 | Marginal I Stylus Zone 2 |  |  |  | <.0001* |
| H | Central Stylus Zone 2 | Marginal II Stylus Zone 2 |  |  |  | <.0001* |
| H | Central Stylus Zone 2 | Marginal I Stylus Zone 2 |  |  |  | <.0001* |
| H | Central Stylus Zone 3 | Central Stylus Zone 2 |  |  |  | <.0001* |
| H | Marginal I Cusp Zone 3 | Marginal I Stylus Zone 1 |  |  |  | <.0001* |
| H | Marginal I Cusp Zone 4 | Marginal I Cusp Zone 3 |  |  |  | <.0001* |
| H | Central Stylus Zone 3 | Lateral Cusp Zone 2 |  |  |  | <.0001* |
| H | Marginal I Cusp Zone 3 | Marginal II Basis Zone 1 |  |  |  | <.0001* |
| H | Marginal I Cusp Zone 3 | Marginal II Stylus Zone 1 |  |  |  | <.0001* |
| H | Marginal I Cusp Zone 3 | Marginal I Basis Zone 1 |  |  |  | <.0001* |
| H | Lateral Cusp Zone 3 | Central Stylus Zone 2 |  |  |  | <.0001* |
| H | Marginal II Cusp Zone 3 | Central Stylus Zone 1 |  |  |  | <.0001* |
| H | Lateral Cusp Zone 3 | Lateral Cusp Zone 2 |  |  |  | <.0001* |
| H | Central Cusp Zone 4 | Central Stylus Zone 4 |  |  |  | <.0001* |
| H | Lateral Stylus Zone 3 | Marginal I Cusp Zone 3 |  |  |  | <.0001* |
| H | Marginal I Cusp Zone 4 | Lateral Stylus Zone 2 |  |  |  | <.0001* |
| H | Marginal II Cusp Zone 3 | Lateral Cusp Zone 1 |  |  |  | <.0001* |
| H | Central Cusp Zone 2 | Marginal I Stylus Zone 3 |  |  |  | <.0001* |
| H | Lateral Stylus Zone 2 | Marginal II Basis Zone 2 |  |  |  | <.0001* |
| H | Lateral Stylus Zone 2 | Marginal I Basis Zone 2 |  |  |  | <.0001* |
| H | Central Cusp Zone 4 | Lateral Cusp Zone 4 |  |  |  | <.0001* |
| H | Marginal I Basis Zone 4 | Marginal I Cusp Zone 1 |  |  |  | <.0001* |
| H | Marginal I Basis Zone 4 | Marginal II Cusp Zone 1 |  |  |  | <.0001* |
| H | Central Cusp Zone 3 | Lateral Stylus Zone 3 |  |  |  | <.0001* |
| H | Central Cusp Zone 2 | Marginal II Stylus Zone 3 |  |  |  | <.0001* |
| H | Lateral Stylus Zone 3 | Lateral Stylus Zone 2 |  |  |  | <.0001* |
| H | Marginal II Stylus Zone 3 | Marginal I Cusp Zone 1 |  |  |  | <.0001* |
| H | Marginal II Cusp Zone 4 | Marginal II Cusp Zone 3 |  |  |  | <.0001* |
| H | Marginal II Stylus Zone 3 | Marginal II Cusp Zone 1 |  |  |  | <.0001* |
| H | Central Cusp Zone 2 | Marginal I Basis Zone 4 |  |  |  | <.0001* |
| H | Lateral Stylus Zone 2 | Lateral Stylus Zone 1 |  |  |  | <.0001* |
| H | Marginal I Stylus Zone 3 | Marginal I Cusp Zone 1 |  |  |  | <.0001* |
| H | Marginal I Stylus Zone 3 | Marginal II Cusp Zone 1 |  |  |  | <.0001* |
| H | Marginal II Cusp Zone 3 | Marginal I Stylus Zone 1 |  |  |  | <.0001* |
| H | Marginal I Cusp Zone 3 | Marginal II Basis Zone 2 |  |  |  | <.0001* |
| H | Marginal I Cusp Zone 3 | Marginal I Basis Zone 2 |  |  |  | <.0001* |
| H | Central Cusp Zone 3 | Marginal I Cusp Zone 4 |  |  |  | <.0001* |
| H | Marginal II Cusp Zone 3 | Marginal II Basis Zone 1 |  |  |  | <.0001* |
| H | Marginal II Cusp Zone 3 | Marginal II Stylus Zone 1 |  |  |  | <.0001* |
| H | Marginal II Cusp Zone 3 | Marginal I Basis Zone 1 |  |  |  | <.0001* |
| H | Marginal I Cusp Zone 3 | Lateral Stylus Zone 1 |  |  |  | <.0001* |
| H | Lateral Cusp Zone 2 | Marginal II Cusp Zone 2 |  |  |  | <.0001* |
| H | Central Stylus Zone 2 | Marginal II Cusp Zone 2 |  |  |  | <.0001* |
| H | Lateral Stylus Zone 2 | Marginal I Basis Zone 3 |  |  |  | <.0001* |
| H | Marginal II Basis Zone 4 | Central Stylus Zone 1 |  |  |  | <.0001* |
| H | Lateral Stylus Zone 2 | Marginal II Basis Zone 3 |  |  |  | <.0001* |
| H | Marginal II Stylus Zone 4 | Marginal I Stylus Zone 3 |  |  |  | <.0001* |
| H | Marginal II Cusp Zone 4 | Marginal I Cusp Zone 3 |  |  |  | <.0001* |
| H | Central Cusp Zone 2 | Marginal II Basis Zone 4 |  |  |  | <.0001* |
| H | Lateral Stylus Zone 2 | Central Cusp Zone 1 |  |  |  | <.0001* |
| H | Lateral Stylus Zone 2 | Marginal II Stylus Zone 2 |  |  |  | <.0001* |
| H | Lateral Stylus Zone 2 | Marginal I Stylus Zone 2 |  |  |  | <.0001* |
| H | Marginal II Stylus Zone 4 | Marginal II Stylus Zone 3 |  |  |  | <.0001* |
| H | Marginal I Cusp Zone 3 | Marginal I Basis Zone 3 |  |  |  | <.0001* |
| H | Marginal II Basis Zone 4 | Lateral Cusp Zone 1 |  |  |  | <.0001* |
| H | Lateral Cusp Zone 2 | Marginal I Cusp Zone 2 |  |  |  | <.0001* |
| H | Marginal II Stylus Zone 4 | Marginal I Basis Zone 4 |  |  |  | <.0001* |
| H | Marginal I Stylus Zone 4 | Marginal I Stylus Zone 3 |  |  |  | <.0001* |
| H | Marginal II Cusp Zone 3 | Marginal II Basis Zone 2 |  |  |  | <.0001* |
| H | Central Stylus Zone 2 | Marginal I Cusp Zone 2 |  |  |  | <.0001* |
| H | Marginal II Cusp Zone 3 | Marginal I Basis Zone 2 |  |  |  | <.0001* |
| H | Marginal I Cusp Zone 3 | Marginal II Basis Zone 3 |  |  |  | <.0001* |
| H | Marginal I Stylus Zone 4 | Marginal II Stylus Zone 3 |  |  |  | <.0001* |
| H | Marginal II Cusp Zone 4 | Lateral Stylus Zone 2 |  |  |  | <.0001* |
| H | Marginal I Cusp Zone 3 | Central Cusp Zone 1 |  |  |  | <.0001* |
| H | Marginal I Stylus Zone 4 | Marginal I Basis Zone 4 |  |  |  | <.0001* |
| H | Marginal I Cusp Zone 3 | Marginal II Stylus Zone 2 |  |  |  | <.0001* |
| H | Marginal II Cusp Zone 3 | Lateral Stylus Zone 1 |  |  |  | <.0001* |
| H | Marginal I Cusp Zone 3 | Marginal I Stylus Zone 2 |  |  |  | <.0001* |
| H | Marginal II Basis Zone 4 | Marginal I Stylus Zone 1 |  |  |  | <.0001* |
| H | Marginal I Cusp Zone 4 | Central Stylus Zone 2 |  |  |  | <.0001* |
| H | Marginal I Cusp Zone 4 | Lateral Cusp Zone 2 |  |  |  | <.0001* |
| H | Marginal II Basis Zone 4 | Marginal II Basis Zone 1 |  |  |  | <.0001* |
| H | Marginal II Basis Zone 4 | Marginal II Stylus Zone 1 |  |  |  | <.0001* |
| H | Marginal II Basis Zone 4 | Marginal I Basis Zone 1 |  |  |  | <.0001* |
| H | Marginal I Basis Zone 4 | Central Stylus Zone 1 |  |  |  | <.0001* |
| H | Marginal II Stylus Zone 3 | Central Stylus Zone 1 |  |  |  | <.0001* |
| H | Lateral Cusp Zone 4 | Central Cusp Zone 3 |  |  |  | <.0001* |
| H | Lateral Stylus Zone 3 | Central Stylus Zone 2 |  |  |  | <.0001* |
| H | Marginal I Stylus Zone 3 | Central Stylus Zone 1 |  |  |  | <.0001* |
| H | Lateral Stylus Zone 3 | Lateral Cusp Zone 2 |  |  |  | <.0001* |
| H | Marginal I Basis Zone 4 | Lateral Cusp Zone 1 |  |  |  | <.0001* |
| H | Marginal II Stylus Zone 4 | Marginal II Basis Zone 4 |  |  |  | <.0001* |
| H | Marginal II Cusp Zone 3 | Marginal I Basis Zone 3 |  |  |  | <.0001* |
| H | Marginal II Stylus Zone 3 | Lateral Cusp Zone 1 |  |  |  | <.0001* |
| H | Central Stylus Zone 4 | Central Cusp Zone 3 |  |  |  | <.0001* |
| H | Marginal II Cusp Zone 3 | Marginal II Basis Zone 3 |  |  |  | <.0001* |
| H | Marginal I Stylus Zone 3 | Lateral Cusp Zone 1 |  |  |  | <.0001* |
| H | Central Cusp Zone 2 | Marginal II Cusp Zone 3 |  |  |  | <.0001* |
| H | Marginal I Stylus Zone 4 | Marginal II Basis Zone 4 |  |  |  | <.0001* |
| H | Marginal II Cusp Zone 3 | Central Cusp Zone 1 |  |  |  | <.0001* |
| H | Marginal II Cusp Zone 3 | Marginal II Stylus Zone 2 |  |  |  | <.0001* |
| H | Marginal I Basis Zone 4 | Marginal I Stylus Zone 1 |  |  |  | <.0001* |
| H | Marginal II Cusp Zone 3 | Marginal I Stylus Zone 2 |  |  |  | <.0001* |
| H | Central Cusp Zone 3 | Lateral Cusp Zone 3 |  |  |  | <.0001* |
| H | Lateral Stylus Zone 2 | Marginal II Cusp Zone 2 |  |  |  | <.0001* |
| H | Marginal II Stylus Zone 3 | Marginal I Stylus Zone 1 |  |  |  | <.0001* |
| H | Marginal II Basis Zone 4 | Marginal II Basis Zone 2 |  |  |  | <.0001* |
| H | Marginal I Basis Zone 4 | Marginal II Basis Zone 1 |  |  |  | <.0001* |
| H | Marginal II Basis Zone 4 | Marginal I Basis Zone 2 |  |  |  | <.0001* |
| H | Marginal I Basis Zone 4 | Marginal II Stylus Zone 1 |  |  |  | <.0001* |
| H | Marginal I Basis Zone 4 | Marginal I Basis Zone 1 |  |  |  | <.0001* |
| H | Marginal II Stylus Zone 3 | Marginal II Basis Zone 1 |  |  |  | <.0001* |
| H | Central Cusp Zone 3 | Central Stylus Zone 3 |  |  |  | <.0001* |
| H | Marginal II Stylus Zone 3 | Marginal II Stylus Zone 1 |  |  |  | <.0001* |
| H | Marginal II Stylus Zone 3 | Marginal I Basis Zone 1 |  |  |  | <.0001* |
| H | Marginal I Stylus Zone 3 | Marginal I Stylus Zone 1 |  |  |  | <.0001* |
| H | Marginal I Stylus Zone 3 | Marginal II Basis Zone 1 |  |  |  | <.0001* |
| H | Marginal II Basis Zone 4 | Lateral Stylus Zone 1 |  |  |  | <.0001* |
| H | Marginal I Stylus Zone 3 | Marginal II Stylus Zone 1 |  |  |  | <.0001* |
| H | Marginal I Stylus Zone 3 | Marginal I Basis Zone 1 |  |  |  | <.0001* |
| H | Marginal I Cusp Zone 3 | Marginal II Cusp Zone 2 |  |  |  | <.0001* |
| H | Lateral Stylus Zone 2 | Marginal I Cusp Zone 2 |  |  |  | <.0001* |
| H | Central Stylus Zone 3 | Marginal I Stylus Zone 4 |  |  |  | <.0001* |
| H | Central Cusp Zone 2 | Marginal I Cusp Zone 3 |  |  |  | <.0001* |
| H | Marginal II Cusp Zone 4 | Central Stylus Zone 2 |  |  |  | <.0001* |
| H | Marginal II Cusp Zone 4 | Lateral Cusp Zone 2 |  |  |  | <.0001* |
| H | Lateral Cusp Zone 3 | Marginal I Stylus Zone 4 |  |  |  | <.0001* |
| H | Marginal II Stylus Zone 4 | Marginal II Cusp Zone 3 |  |  |  | <.0001* |
| H | Marginal II Basis Zone 4 | Marginal I Basis Zone 3 |  |  |  | <.0001* |
| H | Marginal I Basis Zone 4 | Marginal II Basis Zone 2 |  |  |  | <.0001* |
| H | Marginal I Basis Zone 4 | Marginal I Basis Zone 2 |  |  |  | <.0001* |
| H | Central Stylus Zone 3 | Marginal II Stylus Zone 4 |  |  |  | <.0001* |
| H | Marginal II Stylus Zone 3 | Marginal II Basis Zone 2 |  |  |  | <.0001* |
| H | Marginal II Stylus Zone 3 | Marginal I Basis Zone 2 |  |  |  | <.0001* |
| H | Marginal I Cusp Zone 3 | Marginal I Cusp Zone 2 |  |  |  | <.0001* |
| H | Marginal II Basis Zone 4 | Marginal II Basis Zone 3 |  |  |  | <.0001* |
| H | Lateral Cusp Zone 3 | Marginal II Stylus Zone 4 |  |  |  | <.0001* |
| H | Marginal I Stylus Zone 4 | Marginal II Cusp Zone 3 |  |  |  | <.0001* |
| H | Central Cusp Zone 2 | Lateral Stylus Zone 2 |  |  |  | <.0001* |
| H | Marginal I Stylus Zone 3 | Marginal II Basis Zone 2 |  |  |  | <.0001* |
| H | Marginal II Basis Zone 4 | Central Cusp Zone 1 |  |  |  | <.0001* |
| H | Marginal I Stylus Zone 3 | Marginal I Basis Zone 2 |  |  |  | <.0001* |
| H | Marginal I Basis Zone 4 | Lateral Stylus Zone 1 |  |  |  | <.0001* |
| H | Marginal II Basis Zone 4 | Marginal II Stylus Zone 2 |  |  |  | <.0001* |
| H | Marginal II Stylus Zone 3 | Lateral Stylus Zone 1 |  |  |  | <.0001* |
| H | Marginal II Basis Zone 4 | Marginal I Stylus Zone 2 |  |  |  | <.0001* |
| H | Marginal II Cusp Zone 3 | Marginal II Cusp Zone 2 |  |  |  | <.0001* |
| H | Marginal I Stylus Zone 3 | Lateral Stylus Zone 1 |  |  |  | <.0001* |
| H | Marginal I Cusp Zone 2 | Marginal I Cusp Zone 1 |  |  |  | <.0001* |
| H | Marginal I Cusp Zone 2 | Marginal II Cusp Zone 1 |  |  |  | <.0001* |
| H | Marginal I Basis Zone 4 | Marginal I Basis Zone 3 |  |  |  | <.0001* |
| H | Marginal II Stylus Zone 4 | Marginal I Cusp Zone 3 |  |  |  | <.0001* |
| H | Marginal II Stylus Zone 3 | Marginal I Basis Zone 3 |  |  |  | <.0001* |
| H | Marginal I Basis Zone 4 | Marginal II Basis Zone 3 |  |  |  | <.0001* |
| H | Marginal II Cusp Zone 3 | Marginal I Cusp Zone 2 |  |  |  | <.0001* |
| H | Marginal II Stylus Zone 3 | Marginal II Basis Zone 3 |  |  |  | <.0001* |
| H | Marginal I Stylus Zone 3 | Marginal I Basis Zone 3 |  |  |  | <.0001* |
| H | Central Stylus Zone 3 | Central Cusp Zone 2 |  |  |  | <.0001* |
| H | Marginal I Basis Zone 4 | Central Cusp Zone 1 |  |  |  | <.0001* |
| H | Marginal I Stylus Zone 4 | Marginal I Cusp Zone 3 |  |  |  | <.0001* |
| H | Marginal I Basis Zone 4 | Marginal II Stylus Zone 2 |  |  |  | <.0001* |
| H | Marginal II Stylus Zone 3 | Central Cusp Zone 1 |  |  |  | <.0001* |
| H | Marginal I Basis Zone 4 | Marginal I Stylus Zone 2 |  |  |  | <.0001* |
| H | Marginal I Stylus Zone 3 | Marginal II Basis Zone 3 |  |  |  | <.0001* |
| H | Marginal II Stylus Zone 3 | Marginal II Stylus Zone 2 |  |  |  | <.0001* |
| H | Marginal II Cusp Zone 2 | Marginal I Cusp Zone 1 |  |  |  | <.0001* |
| H | Marginal II Cusp Zone 2 | Marginal II Cusp Zone 1 |  |  |  | <.0001* |
| H | Marginal II Stylus Zone 3 | Marginal I Stylus Zone 2 |  |  |  | <.0001* |
| H | Lateral Cusp Zone 3 | Central Cusp Zone 2 |  |  |  | <.0001* |
| H | Marginal II Stylus Zone 4 | Lateral Stylus Zone 2 |  |  |  | <.0001* |
| H | Marginal I Stylus Zone 3 | Central Cusp Zone 1 |  |  |  | <.0001* |
| H | Marginal I Stylus Zone 3 | Marginal II Stylus Zone 2 |  |  |  | <.0001* |
| H | Marginal I Stylus Zone 3 | Marginal I Stylus Zone 2 |  |  |  | <.0001* |
| H | Marginal I Stylus Zone 4 | Lateral Stylus Zone 2 |  |  |  | <.0001* |
| H | Lateral Cusp Zone 2 | Marginal I Stylus Zone 3 |  |  |  | <.0001* |
| H | Central Stylus Zone 2 | Marginal I Stylus Zone 3 |  |  |  | <.0001* |
| H | Marginal II Basis Zone 4 | Marginal II Cusp Zone 2 |  |  |  | <.0001* |
| H | Lateral Cusp Zone 2 | Marginal II Stylus Zone 3 |  |  |  | <.0001* |
| H | Central Stylus Zone 2 | Marginal II Stylus Zone 3 |  |  |  | <.0001* |
| H | Lateral Cusp Zone 2 | Marginal I Basis Zone 4 |  |  |  | <.0001* |
| H | Central Stylus Zone 2 | Marginal I Basis Zone 4 |  |  |  | <.0001* |
| H | Lateral Stylus Zone 4 | Central Cusp Zone 3 |  |  |  | <.0001* |
| H | Marginal I Cusp Zone 4 | Marginal I Stylus Zone 4 |  |  |  | <.0001* |
| H | Central Cusp Zone 2 | Central Stylus Zone 2 |  |  |  | <.0001* |
| H | Central Cusp Zone 2 | Lateral Cusp Zone 2 |  |  |  | <.0001* |
| H | Marginal I Cusp Zone 4 | Marginal II Stylus Zone 4 |  |  |  | <.0001* |
| H | Marginal II Basis Zone 4 | Marginal I Cusp Zone 2 |  |  |  | <.0001* |
| H | Lateral Stylus Zone 3 | Marginal I Stylus Zone 4 |  |  |  | <.0001* |
| H | Marginal I Cusp Zone 2 | Central Stylus Zone 1 |  |  |  | <.0001* |
| H | Marginal I Basis Zone 4 | Marginal II Cusp Zone 2 |  |  |  | <.0001* |
| H | Lateral Stylus Zone 3 | Marginal II Stylus Zone 4 |  |  |  | <.0001* |
| H | Marginal I Stylus Zone 2 | Marginal I Cusp Zone 1 |  |  |  | <.0001* |
| H | Marginal I Stylus Zone 2 | Marginal II Cusp Zone 1 |  |  |  | <.0001* |
| H | Marginal II Stylus Zone 3 | Marginal II Cusp Zone 2 |  |  |  | <.0001* |
| H | Marginal II Stylus Zone 2 | Marginal I Cusp Zone 1 |  |  |  | <.0001* |
| H | Marginal II Stylus Zone 2 | Marginal II Cusp Zone 1 |  |  |  | <.0001* |
| H | Marginal I Cusp Zone 2 | Lateral Cusp Zone 1 |  |  |  | <.0001* |
| H | Lateral Cusp Zone 2 | Marginal II Basis Zone 4 |  |  |  | <.0001* |
| H | Central Cusp Zone 1 | Marginal I Cusp Zone 1 |  |  |  | <.0001* |
| H | Central Cusp Zone 1 | Marginal II Cusp Zone 1 |  |  |  | <.0001* |
| H | Central Stylus Zone 2 | Marginal II Basis Zone 4 |  |  |  | <.0001* |
| H | Marginal I Stylus Zone 3 | Marginal II Cusp Zone 2 |  |  |  | <.0001* |
| H | Marginal II Basis Zone 3 | Marginal I Cusp Zone 1 |  |  |  | <.0001* |
| H | Marginal II Basis Zone 3 | Marginal II Cusp Zone 1 |  |  |  | <.0001* |
| H | Marginal II Cusp Zone 2 | Central Stylus Zone 1 |  |  |  | <.0001* |
| H | Marginal I Basis Zone 3 | Marginal I Cusp Zone 1 |  |  |  | <.0001* |
| H | Marginal I Basis Zone 3 | Marginal II Cusp Zone 1 |  |  |  | <.0001* |
| H | Marginal I Cusp Zone 2 | Marginal I Stylus Zone 1 |  |  |  | <.0001* |
| H | Marginal II Stylus Zone 4 | Central Stylus Zone 2 |  |  |  | <.0001* |
| H | Marginal I Basis Zone 4 | Marginal I Cusp Zone 2 |  |  |  | <.0001* |
| H | Marginal II Stylus Zone 4 | Lateral Cusp Zone 2 |  |  |  | <.0001* |
| H | Marginal I Cusp Zone 2 | Marginal II Basis Zone 1 |  |  |  | <.0001* |
| H | Marginal I Cusp Zone 2 | Marginal II Stylus Zone 1 |  |  |  | <.0001* |
| H | Marginal I Cusp Zone 2 | Marginal I Basis Zone 1 |  |  |  | <.0001* |
| H | Marginal II Stylus Zone 3 | Marginal I Cusp Zone 2 |  |  |  | <.0001* |
| H | Marginal II Cusp Zone 2 | Lateral Cusp Zone 1 |  |  |  | <.0001* |
| H | Central Stylus Zone 3 | Marginal II Cusp Zone 4 |  |  |  | <.0001* |
| H | Marginal I Cusp Zone 4 | Central Cusp Zone 2 |  |  |  | <.0001* |
| H | Marginal I Stylus Zone 4 | Central Stylus Zone 2 |  |  |  | <.0001* |
| H | Marginal I Stylus Zone 3 | Marginal I Cusp Zone 2 |  |  |  | <.0001* |
| H | Marginal I Stylus Zone 4 | Lateral Cusp Zone 2 |  |  |  | <.0001* |
| H | Lateral Stylus Zone 2 | Marginal I Stylus Zone 3 |  |  |  | <.0001* |
| H | Lateral Cusp Zone 3 | Marginal II Cusp Zone 4 |  |  |  | <.0001* |
| H | Lateral Stylus Zone 1 | Marginal I Cusp Zone 1 |  |  |  | <.0001* |
| H | Lateral Stylus Zone 1 | Marginal II Cusp Zone 1 |  |  |  | <.0001* |
| H | Lateral Stylus Zone 2 | Marginal II Stylus Zone 3 |  |  |  | <.0001* |
| H | Lateral Stylus Zone 3 | Central Cusp Zone 2 |  |  |  | <.0001* |
| H | Marginal II Cusp Zone 2 | Marginal I Stylus Zone 1 |  |  |  | <.0001* |
| H | Marginal II Cusp Zone 4 | Marginal I Stylus Zone 4 |  |  |  | <.0001* |
| H | Lateral Cusp Zone 4 | Lateral Stylus Zone 4 |  |  |  | <.0001* |
| H | Lateral Stylus Zone 2 | Marginal I Basis Zone 4 |  |  |  | <.0001* |
| H | Marginal II Cusp Zone 2 | Marginal II Basis Zone 1 |  |  |  | <.0001* |
| H | Marginal I Basis Zone 2 | Marginal I Cusp Zone 1 |  |  |  | <.0001* |
| H | Marginal II Cusp Zone 2 | Marginal II Stylus Zone 1 |  |  |  | <.0001* |
| H | Marginal I Basis Zone 2 | Marginal II Cusp Zone 1 |  |  |  | <.0001* |
| H | Marginal II Cusp Zone 2 | Marginal I Basis Zone 1 |  |  |  | <.0001* |
| H | Marginal II Basis Zone 2 | Marginal I Cusp Zone 1 |  |  |  | <.0001* |
| H | Marginal II Basis Zone 2 | Marginal II Cusp Zone 1 |  |  |  | <.0001* |
| H | Marginal I Cusp Zone 3 | Marginal I Stylus Zone 3 |  |  |  | <.0001* |
| H | Marginal II Cusp Zone 4 | Marginal II Stylus Zone 4 |  |  |  | <.0001* |
| H | Lateral Cusp Zone 2 | Marginal II Cusp Zone 3 |  |  |  | <.0001* |
| H | Central Stylus Zone 4 | Lateral Stylus Zone 4 |  |  |  | <.0001* |
| H | Marginal I Cusp Zone 2 | Marginal II Basis Zone 2 |  |  |  | <.0001* |
| H | Central Stylus Zone 2 | Marginal II Cusp Zone 3 |  |  |  | 0.0187* |
| H | Marginal I Cusp Zone 2 | Marginal I Basis Zone 2 |  |  |  | <.0001* |
| H | Marginal I Cusp Zone 3 | Marginal II Stylus Zone 3 |  |  |  | <.0001* |
| H | Marginal I Cusp Zone 3 | Marginal I Basis Zone 4 |  |  |  | <.0001* |
| H | Marginal I Cusp Zone 2 | Lateral Stylus Zone 1 |  |  |  | <.0001* |
| H | Marginal I Stylus Zone 2 | Central Stylus Zone 1 |  |  |  | 0.0603 |
| H | Marginal II Stylus Zone 2 | Central Stylus Zone 1 |  |  |  | 0.0957 |
| H | Central Cusp Zone 1 | Central Stylus Zone 1 |  |  |  | 0.0725 |
| H | Marginal II Basis Zone 3 | Central Stylus Zone 1 |  |  |  | 0.2337 |
| H | Lateral Stylus Zone 2 | Marginal II Basis Zone 4 |  |  |  | <.0001* |
| H | Marginal II Cusp Zone 2 | Marginal II Basis Zone 2 |  |  |  | 0.0013* |
| H | Central Stylus Zone 3 | Lateral Stylus Zone 3 |  |  |  | 0.1828 |
| H | Marginal I Basis Zone 1 | Marginal I Cusp Zone 1 |  |  |  | <.0001* |
| H | Marginal II Stylus Zone 1 | Marginal I Cusp Zone 1 |  |  |  | 0.0001* |
| H | Marginal I Basis Zone 1 | Marginal II Cusp Zone 1 |  |  |  | 0.0003* |
| H | Marginal II Cusp Zone 2 | Marginal I Basis Zone 2 |  |  |  | 0.0003* |
| H | Marginal II Stylus Zone 1 | Marginal II Cusp Zone 1 |  |  |  | 0.0006* |
| H | Marginal II Basis Zone 1 | Marginal I Cusp Zone 1 |  |  |  | 0.0005* |
| H | Marginal I Stylus Zone 2 | Lateral Cusp Zone 1 |  |  |  | <.0001* |
| H | Marginal II Basis Zone 1 | Marginal II Cusp Zone 1 |  |  |  | 0.0022* |
| H | Marginal II Stylus Zone 2 | Lateral Cusp Zone 1 |  |  |  | 0.0002* |
| H | Marginal I Basis Zone 3 | Central Stylus Zone 1 |  |  |  | 0.2836 |
| H | Central Cusp Zone 1 | Lateral Cusp Zone 1 |  |  |  | <.0001* |
| H | Marginal I Stylus Zone 1 | Marginal I Cusp Zone 1 |  |  |  | <.0001* |
| H | Marginal I Stylus Zone 1 | Marginal II Cusp Zone 1 |  |  |  | 0.0005* |
| H | Marginal II Cusp Zone 3 | Marginal I Stylus Zone 3 |  |  |  | 0.0006* |
| H | Lateral Cusp Zone 3 | Lateral Stylus Zone 3 |  |  |  | <.0001* |
| H | Marginal I Cusp Zone 2 | Marginal I Basis Zone 3 |  |  |  | 0.0004* |
| H | Marginal II Basis Zone 3 | Lateral Cusp Zone 1 |  |  |  | 0.0032* |
| H | Marginal II Cusp Zone 2 | Lateral Stylus Zone 1 |  |  |  | 0.0008* |
| H | Lateral Cusp Zone 2 | Marginal I Cusp Zone 3 |  |  |  | 0.0001* |
| H | Central Stylus Zone 3 | Marginal I Cusp Zone 4 |  |  |  | 0.3074 |
| H | Central Stylus Zone 2 | Marginal I Cusp Zone 3 |  |  |  | 0.4870 |
| H | Marginal II Cusp Zone 3 | Marginal II Stylus Zone 3 |  |  |  | 0.0111* |
| H | Marginal II Cusp Zone 4 | Central Cusp Zone 2 |  |  |  | <.0001* |
| H | Marginal I Cusp Zone 3 | Marginal II Basis Zone 4 |  |  |  | <.0001* |
| H | Marginal I Cusp Zone 2 | Marginal II Basis Zone 3 |  |  |  | 0.0108* |
| H | Marginal I Stylus Zone 2 | Marginal I Stylus Zone 1 |  |  |  | 0.0017* |
| H | Marginal I Basis Zone 3 | Lateral Cusp Zone 1 |  |  |  | 0.0021* |
| H | Marginal II Cusp Zone 3 | Marginal I Basis Zone 4 |  |  |  | 0.0001* |
| H | Marginal II Stylus Zone 2 | Marginal I Stylus Zone 1 |  |  |  | 0.0097* |
| H | Central Cusp Zone 1 | Marginal I Stylus Zone 1 |  |  |  | 0.0016* |
| H | Lateral Cusp Zone 3 | Marginal I Cusp Zone 4 |  |  |  | <.0001* |
| H | Marginal I Cusp Zone 2 | Central Cusp Zone 1 |  |  |  | 0.0010* |
| H | Lateral Cusp Zone 1 | Marginal I Cusp Zone 1 |  |  |  | 0.0016* |
| H | Marginal I Stylus Zone 2 | Marginal II Basis Zone 1 |  |  |  | 0.0417* |
| H | Lateral Cusp Zone 1 | Marginal II Cusp Zone 1 |  |  |  | 0.0088* |
| H | Marginal II Stylus Zone 2 | Marginal II Basis Zone 1 |  |  |  | 0.1031 |
| H | Marginal I Stylus Zone 2 | Marginal II Stylus Zone 1 |  |  |  | 0.0278* |
| H | Marginal I Stylus Zone 2 | Marginal I Basis Zone 1 |  |  |  | 0.0158* |
| H | Marginal I Cusp Zone 2 | Marginal II Stylus Zone 2 |  |  |  | 0.0233* |
| H | Marginal II Stylus Zone 2 | Marginal II Stylus Zone 1 |  |  |  | 0.0794 |
| H | Marginal II Stylus Zone 2 | Marginal I Basis Zone 1 |  |  |  | 0.0536 |
| H | Marginal I Cusp Zone 2 | Marginal I Stylus Zone 2 |  |  |  | 0.0068* |
| H | Central Cusp Zone 1 | Marginal II Basis Zone 1 |  |  |  | 0.0468* |
| H | Marginal II Basis Zone 3 | Marginal I Stylus Zone 1 |  |  |  | 0.0772 |
| H | Lateral Stylus Zone 1 | Central Stylus Zone 1 |  |  |  | 0.8293 |
| H | Central Cusp Zone 1 | Marginal II Stylus Zone 1 |  |  |  | 0.0303* |
| H | Central Cusp Zone 1 | Marginal I Basis Zone 1 |  |  |  | 0.0164* |
| H | Lateral Cusp Zone 2 | Lateral Stylus Zone 2 |  |  |  | 0.0088* |
| H | Central Stylus Zone 2 | Lateral Stylus Zone 2 |  |  |  | 0.8939 |
| H | Marginal II Basis Zone 3 | Marginal II Basis Zone 1 |  |  |  | 0.3520 |
| H | Marginal I Basis Zone 3 | Marginal I Stylus Zone 1 |  |  |  | 0.0747 |
| H | Marginal II Basis Zone 3 | Marginal II Stylus Zone 1 |  |  |  | 0.3105 |
| H | Marginal II Basis Zone 3 | Marginal I Basis Zone 1 |  |  |  | 0.2499 |
| H | Marginal II Cusp Zone 2 | Marginal I Basis Zone 3 |  |  |  | 0.1852 |
| H | Central Stylus Zone 1 | Marginal I Cusp Zone 1 |  |  |  | 0.9577 |
| H | Central Stylus Zone 1 | Marginal II Cusp Zone 1 |  |  |  | 0.9745 |
| H | Marginal I Basis Zone 2 | Central Stylus Zone 1 |  |  |  | 0.9751 |
| H | Marginal I Basis Zone 3 | Marginal II Basis Zone 1 |  |  |  | 0.3972 |
| H | Marginal II Basis Zone 2 | Central Stylus Zone 1 |  |  |  | 0.9876 |
| H | Marginal I Basis Zone 3 | Marginal II Stylus Zone 1 |  |  |  | 0.3449 |
| H | Marginal I Basis Zone 3 | Marginal I Basis Zone 1 |  |  |  | 0.2709 |
| H | Marginal I Cusp Zone 4 | Marginal II Cusp Zone 4 |  |  |  | <.0001* |
| H | Lateral Stylus Zone 1 | Lateral Cusp Zone 1 |  |  |  | 0.1038 |
| H | Marginal II Cusp Zone 2 | Marginal II Basis Zone 3 |  |  |  | 0.5724 |
| H | Marginal II Cusp Zone 2 | Central Cusp Zone 1 |  |  |  | 0.3854 |
| H | Marginal II Cusp Zone 2 | Marginal II Stylus Zone 2 |  |  |  | 0.7740 |
| H | Marginal II Cusp Zone 2 | Marginal I Stylus Zone 2 |  |  |  | 0.6507 |
| H | Marginal I Basis Zone 2 | Lateral Cusp Zone 1 |  |  |  | 0.6009 |
| H | Marginal II Basis Zone 2 | Lateral Cusp Zone 1 |  |  |  | 0.8172 |
| H | Lateral Stylus Zone 3 | Marginal II Cusp Zone 4 |  |  |  | 0.1426 |
| H | Lateral Stylus Zone 2 | Marginal II Cusp Zone 3 |  |  |  | 0.7011 |
| H | Central Cusp Zone 2 | Marginal I Stylus Zone 4 |  |  |  | 0.0730 |
| H | Marginal II Cusp Zone 3 | Marginal II Basis Zone 4 |  |  |  | 0.4994 |
| H | Lateral Stylus Zone 1 | Marginal I Stylus Zone 1 |  |  |  | 0.7367 |
| H | Marginal I Stylus Zone 2 | Marginal II Basis Zone 2 |  |  |  | 0.9599 |
| H | Marginal I Stylus Zone 2 | Marginal I Basis Zone 2 |  |  |  | 0.9081 |
| H | Marginal II Stylus Zone 2 | Marginal II Basis Zone 2 |  |  |  | 0.9874 |
| H | Marginal II Stylus Zone 2 | Marginal I Basis Zone 2 |  |  |  | 0.9706 |
| H | Lateral Stylus Zone 1 | Marginal II Basis Zone 1 |  |  |  | 0.9813 |
| H | Central Cusp Zone 1 | Marginal II Basis Zone 2 |  |  |  | 0.9778 |
| H | Marginal II Basis Zone 4 | Marginal I Stylus Zone 3 |  |  |  | 0.7473 |
| H | Central Cusp Zone 1 | Marginal I Basis Zone 2 |  |  |  | 0.9403 |
| H | Lateral Stylus Zone 1 | Marginal II Stylus Zone 1 |  |  |  | 0.9764 |
| H | Lateral Stylus Zone 1 | Marginal I Basis Zone 1 |  |  |  | 0.9613 |
| H | Central Cusp Zone 2 | Marginal II Stylus Zone 4 |  |  |  | 0.6089 |
| H | Marginal I Basis Zone 2 | Marginal I Stylus Zone 1 |  |  |  | 0.9924 |
| H | Marginal II Basis Zone 3 | Marginal II Basis Zone 2 |  |  |  | 0.9998 |
| H | Marginal II Basis Zone 2 | Marginal I Stylus Zone 1 |  |  |  | 0.9988 |
| H | Marginal II Basis Zone 3 | Marginal I Basis Zone 2 |  |  |  | 0.9994 |
| H | Marginal I Stylus Zone 2 | Lateral Stylus Zone 1 |  |  |  | 0.9917 |
| H | Marginal II Basis Zone 4 | Marginal II Stylus Zone 3 |  |  |  | 0.9897 |
| H | Marginal I Cusp Zone 3 | Marginal II Cusp Zone 3 |  |  |  | 0.9978 |
| H | Marginal II Stylus Zone 2 | Lateral Stylus Zone 1 |  |  |  | 0.9987 |
| H | Marginal I Basis Zone 2 | Marginal II Basis Zone 1 |  |  |  | 0.9999 |
| H | Marginal II Basis Zone 4 | Marginal I Basis Zone 4 |  |  |  | 0.7093 |
| H | Marginal I Basis Zone 1 | Central Stylus Zone 1 |  |  |  | 1.0000 |
| H | Marginal II Stylus Zone 1 | Central Stylus Zone 1 |  |  |  | 1.0000 |
| H | Marginal II Basis Zone 2 | Marginal II Basis Zone 1 |  |  |  | 1.0000 |
| H | Central Cusp Zone 1 | Lateral Stylus Zone 1 |  |  |  | 0.9967 |
| H | Marginal I Basis Zone 2 | Marginal II Stylus Zone 1 |  |  |  | 0.9999 |
| H | Marginal I Basis Zone 3 | Marginal II Basis Zone 2 |  |  |  | 1.0000 |
| H | Marginal I Basis Zone 2 | Marginal I Basis Zone 1 |  |  |  | 0.9999 |
| H | Marginal II Basis Zone 1 | Central Stylus Zone 1 |  |  |  | 1.0000 |
| H | Marginal II Basis Zone 2 | Marginal II Stylus Zone 1 |  |  |  | 1.0000 |
| H | Marginal I Basis Zone 3 | Marginal I Basis Zone 2 |  |  |  | 0.9999 |
| H | Marginal II Basis Zone 2 | Marginal I Basis Zone 1 |  |  |  | 1.0000 |
| H | Marginal II Basis Zone 3 | Lateral Stylus Zone 1 |  |  |  | 1.0000 |
| H | Marginal I Stylus Zone 1 | Central Stylus Zone 1 |  |  |  | 1.0000 |
| H | Marginal I Cusp Zone 2 | Marginal II Cusp Zone 2 |  |  |  | 1.0000 |
| H | Marginal I Basis Zone 3 | Lateral Stylus Zone 1 |  |  |  | 1.0000 |
| H | Marginal I Basis Zone 1 | Lateral Cusp Zone 1 |  |  |  | 1.0000 |
| H | Marginal II Stylus Zone 1 | Lateral Cusp Zone 1 |  |  |  | 1.0000 |
| H | Marginal II Basis Zone 1 | Lateral Cusp Zone 1 |  |  |  | 1.0000 |
| H | Lateral Stylus Zone 2 | Marginal I Cusp Zone 3 |  |  |  | 1.0000 |
| H | Marginal I Stylus Zone 2 | Marginal I Basis Zone 3 |  |  |  | 1.0000 |
| H | Marginal I Stylus Zone 1 | Lateral Cusp Zone 1 |  |  |  | 1.0000 |
| H | Marginal II Stylus Zone 2 | Marginal I Basis Zone 3 |  |  |  | 1.0000 |
| H | Lateral Cusp Zone 1 | Central Stylus Zone 1 |  |  |  | 1.0000 |
| H | Lateral Cusp Zone 4 | Central Stylus Zone 4 |  |  |  | 1.0000 |
| H | Marginal I Cusp Zone 4 | Lateral Stylus Zone 3 |  |  |  | 1.0000 |
| H | Central Cusp Zone 1 | Marginal I Basis Zone 3 |  |  |  | 1.0000 |
| H | Lateral Stylus Zone 1 | Marginal II Basis Zone 2 |  |  |  | 1.0000 |
| H | Marginal II Stylus Zone 4 | Marginal I Stylus Zone 4 |  |  |  | 1.0000 |
| H | Lateral Stylus Zone 1 | Marginal I Basis Zone 2 |  |  |  | 1.0000 |
| H | Marginal I Basis Zone 4 | Marginal I Stylus Zone 3 |  |  |  | 1.0000 |
| H | Marginal I Stylus Zone 2 | Marginal II Basis Zone 3 |  |  |  | 1.0000 |
| H | Central Stylus Zone 3 | Lateral Cusp Zone 3 |  |  |  | 1.0000 |
| H | Marginal II Stylus Zone 2 | Marginal II Basis Zone 3 |  |  |  | 1.0000 |
| H | Marginal II Stylus Zone 3 | Marginal I Stylus Zone 3 |  |  |  | 1.0000 |
| H | Marginal I Basis Zone 1 | Marginal I Stylus Zone 1 |  |  |  | 1.0000 |
| H | Marginal II Stylus Zone 1 | Marginal I Stylus Zone 1 |  |  |  | 1.0000 |
| H | Marginal II Basis Zone 3 | Marginal I Basis Zone 3 |  |  |  | 1.0000 |
| H | Central Cusp Zone 1 | Marginal II Basis Zone 3 |  |  |  | 1.0000 |
| H | Marginal II Basis Zone 1 | Marginal I Stylus Zone 1 |  |  |  | 1.0000 |
| H | Marginal I Stylus Zone 2 | Central Cusp Zone 1 |  |  |  | 1.0000 |
| H | Marginal I Basis Zone 4 | Marginal II Stylus Zone 3 |  |  |  | 1.0000 |
| H | Marginal II Stylus Zone 2 | Central Cusp Zone 1 |  |  |  | 1.0000 |
| H | Marginal I Basis Zone 1 | Marginal II Basis Zone 1 |  |  |  | 1.0000 |
| H | Lateral Cusp Zone 2 | Central Stylus Zone 2 |  |  |  | 1.0000 |
| H | Marginal II Stylus Zone 1 | Marginal II Basis Zone 1 |  |  |  | 1.0000 |
| H | Marginal I Stylus Zone 2 | Marginal II Stylus Zone 2 |  |  |  | 1.0000 |
| H | Marginal I Basis Zone 2 | Marginal II Basis Zone 2 |  |  |  | 1.0000 |
| H | Marginal II Cusp Zone 1 | Marginal I Cusp Zone 1 |  |  |  | 1.0000 |
| H | Marginal I Basis Zone 1 | Marginal II Stylus Zone 1 |  |  |  | 1.0000 |
| E | Central Cusp Zone 4 | Marginal I Cusp Zone 1 | 27723.22 | 39 | <.0001* | <.0001* |
| E | Central Cusp Zone 4 | Marginal II Cusp Zone 1 |  |  |  | <.0001* |
| E | Central Cusp Zone 4 | Lateral Stylus Zone 1 |  |  |  | <.0001* |
| E | Central Cusp Zone 4 | Marginal I Stylus Zone 1 |  |  |  | <.0001* |
| E | Central Cusp Zone 4 | Marginal II Stylus Zone 1 |  |  |  | <.0001* |
| E | Central Cusp Zone 4 | Marginal II Basis Zone 1 |  |  |  | <.0001* |
| E | Central Cusp Zone 4 | Marginal I Basis Zone 1 |  |  |  | <.0001* |
| E | Central Cusp Zone 4 | Marginal II Basis Zone 2 |  |  |  | <.0001* |
| E | Central Cusp Zone 4 | Marginal I Basis Zone 2 |  |  |  | <.0001* |
| E | Central Cusp Zone 4 | Lateral Cusp Zone 1 |  |  |  | <.0001* |
| E | Central Cusp Zone 4 | Marginal I Basis Zone 3 |  |  |  | <.0001* |
| E | Central Cusp Zone 4 | Central Stylus Zone 1 |  |  |  | <.0001* |
| E | Central Cusp Zone 4 | Marginal II Basis Zone 3 |  |  |  | <.0001* |
| E | Central Cusp Zone 4 | Marginal I Stylus Zone 2 |  |  |  | <.0001* |
| E | Central Cusp Zone 4 | Marginal II Stylus Zone 2 |  |  |  | <.0001* |
| E | Central Cusp Zone 4 | Central Cusp Zone 1 |  |  |  | <.0001* |
| E | Central Cusp Zone 4 | Marginal II Cusp Zone 2 |  |  |  | <.0001* |
| E | Central Cusp Zone 4 | Marginal I Cusp Zone 2 |  |  |  | <.0001* |
| E | Central Cusp Zone 4 | Marginal I Stylus Zone 3 |  |  |  | <.0001* |
| E | Central Cusp Zone 4 | Marginal II Stylus Zone 3 |  |  |  | <.0001* |
| E | Central Cusp Zone 4 | Marginal I Basis Zone 4 |  |  |  | <.0001* |
| E | Central Cusp Zone 4 | Marginal II Basis Zone 4 |  |  |  | <.0001* |
| E | Central Cusp Zone 4 | Lateral Stylus Zone 2 |  |  |  | <.0001* |
| E | Central Stylus Zone 4 | Marginal I Cusp Zone 1 |  |  |  | <.0001* |
| E | Central Stylus Zone 4 | Marginal II Cusp Zone 1 |  |  |  | <.0001* |
| E | Central Stylus Zone 4 | Lateral Stylus Zone 1 |  |  |  | <.0001* |
| E | Central Stylus Zone 4 | Marginal I Stylus Zone 1 |  |  |  | <.0001* |
| E | Central Stylus Zone 4 | Marginal II Stylus Zone 1 |  |  |  | <.0001* |
| E | Central Stylus Zone 4 | Marginal II Basis Zone 1 |  |  |  | <.0001* |
| E | Central Stylus Zone 4 | Marginal I Basis Zone 1 |  |  |  | <.0001* |
| E | Central Stylus Zone 4 | Marginal II Basis Zone 2 |  |  |  | <.0001* |
| E | Central Stylus Zone 4 | Marginal I Basis Zone 2 |  |  |  | <.0001* |
| E | Central Stylus Zone 4 | Lateral Cusp Zone 1 |  |  |  | <.0001* |
| E | Central Stylus Zone 4 | Marginal I Basis Zone 3 |  |  |  | <.0001* |
| E | Central Stylus Zone 4 | Central Stylus Zone 1 |  |  |  | <.0001* |
| E | Central Stylus Zone 4 | Marginal II Basis Zone 3 |  |  |  | <.0001* |
| E | Central Stylus Zone 4 | Marginal I Stylus Zone 2 |  |  |  | <.0001* |
| E | Central Stylus Zone 4 | Marginal II Stylus Zone 2 |  |  |  | <.0001* |
| E | Central Cusp Zone 4 | Marginal II Cusp Zone 3 |  |  |  | <.0001* |
| E | Lateral Cusp Zone 4 | Marginal I Cusp Zone 1 |  |  |  | <.0001* |
| E | Lateral Cusp Zone 4 | Marginal II Cusp Zone 1 |  |  |  | <.0001* |
| E | Lateral Cusp Zone 4 | Lateral Stylus Zone 1 |  |  |  | <.0001* |
| E | Lateral Cusp Zone 4 | Marginal I Stylus Zone 1 |  |  |  | <.0001* |
| E | Lateral Cusp Zone 4 | Marginal II Stylus Zone 1 |  |  |  | <.0001* |
| E | Lateral Cusp Zone 4 | Marginal II Basis Zone 1 |  |  |  | <.0001* |
| E | Lateral Cusp Zone 4 | Marginal I Basis Zone 1 |  |  |  | <.0001* |
| E | Central Cusp Zone 4 | Marginal I Cusp Zone 3 |  |  |  | <.0001* |
| E | Lateral Cusp Zone 4 | Marginal II Basis Zone 2 |  |  |  | <.0001* |
| E | Lateral Cusp Zone 4 | Marginal I Basis Zone 2 |  |  |  | <.0001* |
| E | Central Cusp Zone 4 | Lateral Cusp Zone 2 |  |  |  | <.0001* |
| E | Lateral Cusp Zone 4 | Lateral Cusp Zone 1 |  |  |  | <.0001* |
| E | Central Stylus Zone 4 | Central Cusp Zone 1 |  |  |  | <.0001* |
| E | Central Cusp Zone 4 | Marginal I Stylus Zone 4 |  |  |  | <.0001* |
| E | Lateral Cusp Zone 4 | Marginal I Basis Zone 3 |  |  |  | <.0001* |
| E | Lateral Cusp Zone 4 | Central Stylus Zone 1 |  |  |  | <.0001* |
| E | Lateral Cusp Zone 4 | Marginal II Basis Zone 3 |  |  |  | <.0001* |
| E | Central Cusp Zone 4 | Marginal II Stylus Zone 4 |  |  |  | <.0001* |
| E | Lateral Cusp Zone 4 | Marginal I Stylus Zone 2 |  |  |  | <.0001* |
| E | Lateral Cusp Zone 4 | Marginal II Stylus Zone 2 |  |  |  | <.0001* |
| E | Central Cusp Zone 4 | Central Stylus Zone 2 |  |  |  | <.0001* |
| E | Central Stylus Zone 4 | Marginal II Cusp Zone 2 |  |  |  | <.0001* |
| E | Central Cusp Zone 4 | Lateral Stylus Zone 3 |  |  |  | <.0001* |
| E | Central Stylus Zone 4 | Marginal I Cusp Zone 2 |  |  |  | <.0001* |
| E | Central Stylus Zone 4 | Marginal I Stylus Zone 3 |  |  |  | <.0001* |
| E | Central Stylus Zone 4 | Marginal II Stylus Zone 3 |  |  |  | <.0001* |
| E | Lateral Cusp Zone 4 | Central Cusp Zone 1 |  |  |  | <.0001* |
| E | Central Stylus Zone 4 | Marginal I Basis Zone 4 |  |  |  | <.0001* |
| E | Central Stylus Zone 4 | Marginal II Basis Zone 4 |  |  |  | <.0001* |
| E | Central Stylus Zone 4 | Lateral Stylus Zone 2 |  |  |  | <.0001* |
| E | Lateral Cusp Zone 4 | Marginal II Cusp Zone 2 |  |  |  | <.0001* |
| E | Lateral Cusp Zone 4 | Marginal I Cusp Zone 2 |  |  |  | <.0001* |
| E | Central Cusp Zone 3 | Marginal I Cusp Zone 1 |  |  |  | <.0001* |
| E | Central Cusp Zone 3 | Marginal II Cusp Zone 1 |  |  |  | <.0001* |
| E | Central Cusp Zone 3 | Lateral Stylus Zone 1 |  |  |  | <.0001* |
| E | Central Cusp Zone 3 | Marginal I Stylus Zone 1 |  |  |  | <.0001* |
| E | Central Cusp Zone 3 | Marginal II Stylus Zone 1 |  |  |  | <.0001* |
| E | Central Cusp Zone 3 | Marginal II Basis Zone 1 |  |  |  | <.0001* |
| E | Central Cusp Zone 3 | Marginal I Basis Zone 1 |  |  |  | <.0001* |
| E | Lateral Cusp Zone 4 | Marginal I Stylus Zone 3 |  |  |  | <.0001* |
| E | Lateral Cusp Zone 4 | Marginal II Stylus Zone 3 |  |  |  | <.0001* |
| E | Lateral Cusp Zone 4 | Marginal I Basis Zone 4 |  |  |  | <.0001* |
| E | Central Cusp Zone 3 | Marginal II Basis Zone 2 |  |  |  | <.0001* |
| E | Central Cusp Zone 3 | Marginal I Basis Zone 2 |  |  |  | <.0001* |
| E | Central Cusp Zone 4 | Central Cusp Zone 2 |  |  |  | <.0001* |
| E | Central Cusp Zone 3 | Lateral Cusp Zone 1 |  |  |  | <.0001* |
| E | Lateral Cusp Zone 4 | Marginal II Basis Zone 4 |  |  |  | <.0001* |
| E | Central Cusp Zone 3 | Marginal I Basis Zone 3 |  |  |  | <.0001* |
| E | Central Cusp Zone 3 | Central Stylus Zone 1 |  |  |  | <.0001* |
| E | Central Cusp Zone 3 | Marginal II Basis Zone 3 |  |  |  | <.0001* |
| E | Lateral Cusp Zone 4 | Lateral Stylus Zone 2 |  |  |  | <.0001* |
| E | Central Cusp Zone 3 | Marginal I Stylus Zone 2 |  |  |  | <.0001* |
| E | Central Cusp Zone 3 | Marginal II Stylus Zone 2 |  |  |  | <.0001* |
| E | Central Stylus Zone 4 | Marginal II Cusp Zone 3 |  |  |  | <.0001* |
| E | Central Cusp Zone 4 | Marginal II Cusp Zone 4 |  |  |  | <.0001* |
| E | Central Cusp Zone 3 | Central Cusp Zone 1 |  |  |  | <.0001* |
| E | Central Stylus Zone 4 | Marginal I Cusp Zone 3 |  |  |  | <.0001* |
| E | Central Stylus Zone 4 | Lateral Cusp Zone 2 |  |  |  | <.0001* |
| E | Central Cusp Zone 4 | Lateral Cusp Zone 3 |  |  |  | <.0001* |
| E | Lateral Stylus Zone 4 | Marginal I Cusp Zone 1 |  |  |  | <.0001* |
| E | Lateral Stylus Zone 4 | Marginal II Cusp Zone 1 |  |  |  | <.0001* |
| E | Lateral Stylus Zone 4 | Lateral Stylus Zone 1 |  |  |  | <.0001* |
| E | Lateral Stylus Zone 4 | Marginal I Stylus Zone 1 |  |  |  | <.0001* |
| E | Lateral Stylus Zone 4 | Marginal II Stylus Zone 1 |  |  |  | <.0001* |
| E | Lateral Stylus Zone 4 | Marginal II Basis Zone 1 |  |  |  | <.0001* |
| E | Lateral Stylus Zone 4 | Marginal I Basis Zone 1 |  |  |  | <.0001* |
| E | Central Cusp Zone 4 | Marginal I Cusp Zone 4 |  |  |  | <.0001* |
| E | Central Stylus Zone 4 | Marginal I Stylus Zone 4 |  |  |  | <.0001* |
| E | Lateral Stylus Zone 4 | Marginal II Basis Zone 2 |  |  |  | <.0001* |
| E | Central Stylus Zone 4 | Marginal II Stylus Zone 4 |  |  |  | <.0001* |
| E | Lateral Stylus Zone 4 | Marginal I Basis Zone 2 |  |  |  | <.0001* |
| E | Central Cusp Zone 3 | Marginal II Cusp Zone 2 |  |  |  | <.0001* |
| E | Lateral Stylus Zone 4 | Lateral Cusp Zone 1 |  |  |  | <.0001* |
| E | Central Stylus Zone 4 | Central Stylus Zone 2 |  |  |  | <.0001* |
| E | Central Cusp Zone 3 | Marginal I Cusp Zone 2 |  |  |  | <.0001* |
| E | Lateral Cusp Zone 4 | Marginal II Cusp Zone 3 |  |  |  | <.0001* |
| E | Lateral Stylus Zone 4 | Marginal I Basis Zone 3 |  |  |  | <.0001* |
| E | Lateral Stylus Zone 4 | Central Stylus Zone 1 |  |  |  | <.0001* |
| E | Central Cusp Zone 3 | Marginal I Stylus Zone 3 |  |  |  | <.0001* |
| E | Lateral Stylus Zone 4 | Marginal II Basis Zone 3 |  |  |  | <.0001* |
| E | Lateral Stylus Zone 4 | Marginal I Stylus Zone 2 |  |  |  | <.0001* |
| E | Central Cusp Zone 4 | Central Stylus Zone 3 |  |  |  | <.0001* |
| E | Central Cusp Zone 3 | Marginal II Stylus Zone 3 |  |  |  | <.0001* |
| E | Lateral Stylus Zone 4 | Marginal II Stylus Zone 2 |  |  |  | <.0001* |
| E | Central Cusp Zone 3 | Marginal I Basis Zone 4 |  |  |  | <.0001* |
| E | Central Stylus Zone 4 | Lateral Stylus Zone 3 |  |  |  | <.0001* |
| E | Central Stylus Zone 3 | Marginal I Cusp Zone 1 |  |  |  | <.0001* |
| E | Central Stylus Zone 3 | Marginal II Cusp Zone 1 |  |  |  | <.0001* |
| E | Central Stylus Zone 3 | Lateral Stylus Zone 1 |  |  |  | <.0001* |
| E | Central Stylus Zone 3 | Marginal I Stylus Zone 1 |  |  |  | <.0001* |
| E | Central Stylus Zone 3 | Marginal II Stylus Zone 1 |  |  |  | <.0001* |
| E | Central Stylus Zone 3 | Marginal II Basis Zone 1 |  |  |  | <.0001* |
| E | Central Stylus Zone 3 | Marginal I Basis Zone 1 |  |  |  | <.0001* |
| E | Lateral Cusp Zone 4 | Marginal I Cusp Zone 3 |  |  |  | <.0001* |
| E | Lateral Cusp Zone 4 | Lateral Cusp Zone 2 |  |  |  | <.0001* |
| E | Central Cusp Zone 3 | Marginal II Basis Zone 4 |  |  |  | <.0001* |
| E | Central Stylus Zone 3 | Marginal II Basis Zone 2 |  |  |  | <.0001* |
| E | Central Stylus Zone 3 | Marginal I Basis Zone 2 |  |  |  | <.0001* |
| E | Central Stylus Zone 3 | Lateral Cusp Zone 1 |  |  |  | <.0001* |
| E | Lateral Cusp Zone 4 | Marginal I Stylus Zone 4 |  |  |  | <.0001* |
| E | Central Cusp Zone 3 | Lateral Stylus Zone 2 |  |  |  | <.0001* |
| E | Lateral Cusp Zone 4 | Marginal II Stylus Zone 4 |  |  |  | <.0001* |
| E | Marginal I Cusp Zone 4 | Marginal I Cusp Zone 1 |  |  |  | <.0001* |
| E | Marginal I Cusp Zone 4 | Marginal II Cusp Zone 1 |  |  |  | <.0001* |
| E | Marginal I Cusp Zone 4 | Lateral Stylus Zone 1 |  |  |  | <.0001* |
| E | Marginal I Cusp Zone 4 | Marginal I Stylus Zone 1 |  |  |  | <.0001* |
| E | Marginal I Cusp Zone 4 | Marginal II Stylus Zone 1 |  |  |  | <.0001* |
| E | Marginal I Cusp Zone 4 | Marginal II Basis Zone 1 |  |  |  | <.0001* |
| E | Marginal I Cusp Zone 4 | Marginal I Basis Zone 1 |  |  |  | <.0001* |
| E | Central Stylus Zone 3 | Marginal I Basis Zone 3 |  |  |  | <.0001* |
| E | Central Stylus Zone 3 | Central Stylus Zone 1 |  |  |  | <.0001* |
| E | Lateral Cusp Zone 4 | Central Stylus Zone 2 |  |  |  | <.0001* |
| E | Central Stylus Zone 3 | Marginal II Basis Zone 3 |  |  |  | <.0001* |
| E | Central Stylus Zone 3 | Marginal I Stylus Zone 2 |  |  |  | <.0001* |
| E | Lateral Stylus Zone 4 | Central Cusp Zone 1 |  |  |  | <.0001* |
| E | Central Cusp Zone 4 | Lateral Stylus Zone 4 |  |  |  | <.0001* |
| E | Lateral Cusp Zone 3 | Marginal I Cusp Zone 1 |  |  |  | <.0001* |
| E | Lateral Cusp Zone 3 | Marginal II Cusp Zone 1 |  |  |  | <.0001* |
| E | Central Stylus Zone 3 | Marginal II Stylus Zone 2 |  |  |  | <.0001* |
| E | Lateral Cusp Zone 3 | Lateral Stylus Zone 1 |  |  |  | <.0001* |
| E | Lateral Cusp Zone 3 | Marginal I Stylus Zone 1 |  |  |  | <.0001* |
| E | Lateral Cusp Zone 3 | Marginal II Stylus Zone 1 |  |  |  | <.0001* |
| E | Lateral Cusp Zone 3 | Marginal II Basis Zone 1 |  |  |  | <.0001* |
| E | Lateral Cusp Zone 3 | Marginal I Basis Zone 1 |  |  |  | <.0001* |
| E | Marginal I Cusp Zone 4 | Marginal II Basis Zone 2 |  |  |  | <.0001* |
| E | Marginal I Cusp Zone 4 | Marginal I Basis Zone 2 |  |  |  | <.0001* |
| E | Marginal I Cusp Zone 4 | Lateral Cusp Zone 1 |  |  |  | <.0001* |
| E | Lateral Cusp Zone 3 | Marginal II Basis Zone 2 |  |  |  | <.0001* |
| E | Lateral Cusp Zone 4 | Lateral Stylus Zone 3 |  |  |  | <.0001* |
| E | Lateral Cusp Zone 3 | Marginal I Basis Zone 2 |  |  |  | <.0001* |
| E | Lateral Cusp Zone 3 | Lateral Cusp Zone 1 |  |  |  | <.0001* |
| E | Marginal II Cusp Zone 4 | Marginal I Cusp Zone 1 |  |  |  | <.0001* |
| E | Marginal II Cusp Zone 4 | Marginal II Cusp Zone 1 |  |  |  | <.0001* |
| E | Marginal II Cusp Zone 4 | Lateral Stylus Zone 1 |  |  |  | <.0001* |
| E | Marginal I Cusp Zone 4 | Marginal I Basis Zone 3 |  |  |  | <.0001* |
| E | Marginal II Cusp Zone 4 | Marginal I Stylus Zone 1 |  |  |  | <.0001* |
| E | Marginal II Cusp Zone 4 | Marginal II Stylus Zone 1 |  |  |  | <.0001* |
| E | Marginal I Cusp Zone 4 | Central Stylus Zone 1 |  |  |  | <.0001* |
| E | Marginal II Cusp Zone 4 | Marginal II Basis Zone 1 |  |  |  | <.0001* |
| E | Marginal II Cusp Zone 4 | Marginal I Basis Zone 1 |  |  |  | <.0001* |
| E | Marginal I Cusp Zone 4 | Marginal II Basis Zone 3 |  |  |  | <.0001* |
| E | Marginal I Cusp Zone 4 | Marginal I Stylus Zone 2 |  |  |  | <.0001* |
| E | Marginal I Cusp Zone 4 | Marginal II Stylus Zone 2 |  |  |  | <.0001* |
| E | Lateral Stylus Zone 4 | Marginal II Cusp Zone 2 |  |  |  | <.0001* |
| E | Lateral Cusp Zone 3 | Marginal I Basis Zone 3 |  |  |  | <.0001* |
| E | Lateral Cusp Zone 3 | Central Stylus Zone 1 |  |  |  | <.0001* |
| E | Lateral Cusp Zone 3 | Marginal II Basis Zone 3 |  |  |  | <.0001* |
| E | Marginal II Cusp Zone 4 | Marginal II Basis Zone 2 |  |  |  | <.0001* |
| E | Marginal II Cusp Zone 4 | Marginal I Basis Zone 2 |  |  |  | <.0001* |
| E | Lateral Cusp Zone 3 | Marginal I Stylus Zone 2 |  |  |  | <.0001* |
| E | Lateral Cusp Zone 3 | Marginal II Stylus Zone 2 |  |  |  | <.0001* |
| E | Central Stylus Zone 4 | Central Cusp Zone 2 |  |  |  | <.0001* |
| E | Marginal II Cusp Zone 4 | Lateral Cusp Zone 1 |  |  |  | <.0001* |
| E | Lateral Stylus Zone 4 | Marginal I Cusp Zone 2 |  |  |  | <.0001* |
| E | Central Stylus Zone 3 | Central Cusp Zone 1 |  |  |  | <.0001* |
| E | Lateral Stylus Zone 4 | Marginal I Stylus Zone 3 |  |  |  | <.0001* |
| E | Lateral Stylus Zone 4 | Marginal II Stylus Zone 3 |  |  |  | <.0001* |
| E | Marginal II Cusp Zone 4 | Marginal I Basis Zone 3 |  |  |  | <.0001* |
| E | Marginal II Cusp Zone 4 | Central Stylus Zone 1 |  |  |  | <.0001* |
| E | Lateral Stylus Zone 4 | Marginal I Basis Zone 4 |  |  |  | <.0001* |
| E | Marginal II Cusp Zone 4 | Marginal II Basis Zone 3 |  |  |  | <.0001* |
| E | Marginal II Cusp Zone 4 | Marginal I Stylus Zone 2 |  |  |  | <.0001* |
| E | Marginal II Cusp Zone 4 | Marginal II Stylus Zone 2 |  |  |  | <.0001* |
| E | Lateral Stylus Zone 4 | Marginal II Basis Zone 4 |  |  |  | <.0001* |
| E | Marginal I Cusp Zone 4 | Central Cusp Zone 1 |  |  |  | <.0001* |
| E | Central Cusp Zone 3 | Marginal II Cusp Zone 3 |  |  |  | <.0001* |
| E | Central Cusp Zone 2 | Marginal I Cusp Zone 1 |  |  |  | <.0001* |
| E | Central Stylus Zone 3 | Marginal II Cusp Zone 2 |  |  |  | <.0001* |
| E | Central Cusp Zone 2 | Marginal II Cusp Zone 1 |  |  |  | <.0001* |
| E | Central Cusp Zone 2 | Lateral Stylus Zone 1 |  |  |  | <.0001* |
| E | Central Cusp Zone 2 | Marginal I Stylus Zone 1 |  |  |  | <.0001* |
| E | Central Cusp Zone 2 | Marginal II Stylus Zone 1 |  |  |  | <.0001* |
| E | Central Cusp Zone 2 | Marginal II Basis Zone 1 |  |  |  | <.0001* |
| E | Central Cusp Zone 2 | Marginal I Basis Zone 1 |  |  |  | <.0001* |
| E | Lateral Stylus Zone 4 | Lateral Stylus Zone 2 |  |  |  | <.0001* |
| E | Lateral Cusp Zone 3 | Central Cusp Zone 1 |  |  |  | <.0001* |
| E | Central Stylus Zone 3 | Marginal I Cusp Zone 2 |  |  |  | <.0001* |
| E | Central Cusp Zone 2 | Marginal II Basis Zone 2 |  |  |  | <.0001* |
| E | Central Cusp Zone 3 | Marginal I Cusp Zone 3 |  |  |  | <.0001* |
| E | Central Stylus Zone 3 | Marginal I Stylus Zone 3 |  |  |  | <.0001* |
| E | Central Cusp Zone 2 | Marginal I Basis Zone 2 |  |  |  | <.0001* |
| E | Central Cusp Zone 3 | Lateral Cusp Zone 2 |  |  |  | <.0001* |
| E | Central Cusp Zone 4 | Central Cusp Zone 3 |  |  |  | <.0001* |
| E | Central Stylus Zone 3 | Marginal II Stylus Zone 3 |  |  |  | <.0001* |
| E | Central Stylus Zone 4 | Marginal II Cusp Zone 4 |  |  |  | <.0001* |
| E | Lateral Cusp Zone 4 | Central Cusp Zone 2 |  |  |  | <.0001* |
| E | Central Cusp Zone 2 | Lateral Cusp Zone 1 |  |  |  | <.0001* |
| E | Central Stylus Zone 3 | Marginal I Basis Zone 4 |  |  |  | <.0001* |
| E | Marginal II Cusp Zone 4 | Central Cusp Zone 1 |  |  |  | <.0001* |
| E | Marginal I Cusp Zone 4 | Marginal II Cusp Zone 2 |  |  |  | <.0001* |
| E | Central Cusp Zone 2 | Marginal I Basis Zone 3 |  |  |  | <.0001* |
| E | Central Cusp Zone 2 | Central Stylus Zone 1 |  |  |  | <.0001* |
| E | Central Cusp Zone 3 | Marginal I Stylus Zone 4 |  |  |  | <.0001* |
| E | Central Cusp Zone 2 | Marginal II Basis Zone 3 |  |  |  | <.0001* |
| E | Central Stylus Zone 3 | Marginal II Basis Zone 4 |  |  |  | <.0001* |
| E | Central Cusp Zone 2 | Marginal I Stylus Zone 2 |  |  |  | <.0001* |
| E | Central Cusp Zone 3 | Marginal II Stylus Zone 4 |  |  |  | <.0001* |
| E | Lateral Cusp Zone 3 | Marginal II Cusp Zone 2 |  |  |  | <.0001* |
| E | Marginal I Cusp Zone 4 | Marginal I Cusp Zone 2 |  |  |  | <.0001* |
| E | Central Cusp Zone 2 | Marginal II Stylus Zone 2 |  |  |  | <.0001* |
| E | Central Stylus Zone 4 | Lateral Cusp Zone 3 |  |  |  | <.0001* |
| E | Marginal I Cusp Zone 4 | Marginal I Stylus Zone 3 |  |  |  | <.0001* |
| E | Central Cusp Zone 3 | Central Stylus Zone 2 |  |  |  | <.0001* |
| E | Marginal I Cusp Zone 4 | Marginal II Stylus Zone 3 |  |  |  | <.0001* |
| E | Central Stylus Zone 3 | Lateral Stylus Zone 2 |  |  |  | <.0001* |
| E | Lateral Cusp Zone 3 | Marginal I Cusp Zone 2 |  |  |  | <.0001* |
| E | Central Stylus Zone 4 | Marginal I Cusp Zone 4 |  |  |  | <.0001* |
| E | Marginal I Cusp Zone 4 | Marginal I Basis Zone 4 |  |  |  | <.0001* |
| E | Lateral Cusp Zone 3 | Marginal I Stylus Zone 3 |  |  |  | <.0001* |
| E | Lateral Cusp Zone 3 | Marginal II Stylus Zone 3 |  |  |  | <.0001* |
| E | Marginal II Cusp Zone 4 | Marginal II Cusp Zone 2 |  |  |  | <.0001* |
| E | Lateral Cusp Zone 3 | Marginal I Basis Zone 4 |  |  |  | <.0001* |
| E | Marginal I Cusp Zone 4 | Marginal II Basis Zone 4 |  |  |  | <.0001* |
| E | Central Cusp Zone 3 | Lateral Stylus Zone 3 |  |  |  | <.0001* |
| E | Marginal II Cusp Zone 4 | Marginal I Cusp Zone 2 |  |  |  | <.0001* |
| E | Lateral Cusp Zone 4 | Marginal II Cusp Zone 4 |  |  |  | <.0001* |
| E | Lateral Cusp Zone 3 | Marginal II Basis Zone 4 |  |  |  | <.0001* |
| E | Marginal II Cusp Zone 4 | Marginal I Stylus Zone 3 |  |  |  | <.0001* |
| E | Marginal I Cusp Zone 4 | Lateral Stylus Zone 2 |  |  |  | <.0001* |
| E | Marginal II Cusp Zone 4 | Marginal II Stylus Zone 3 |  |  |  | <.0001* |
| E | Central Stylus Zone 4 | Central Stylus Zone 3 |  |  |  | <.0001* |
| E | Lateral Stylus Zone 3 | Marginal I Cusp Zone 1 |  |  |  | <.0001* |
| E | Lateral Stylus Zone 3 | Marginal II Cusp Zone 1 |  |  |  | <.0001* |
| E | Lateral Stylus Zone 3 | Lateral Stylus Zone 1 |  |  |  | <.0001* |
| E | Lateral Stylus Zone 3 | Marginal I Stylus Zone 1 |  |  |  | <.0001* |
| E | Lateral Stylus Zone 3 | Marginal II Stylus Zone 1 |  |  |  | <.0001* |
| E | Lateral Stylus Zone 3 | Marginal II Basis Zone 1 |  |  |  | <.0001* |
| E | Lateral Stylus Zone 3 | Marginal I Basis Zone 1 |  |  |  | <.0001* |
| E | Central Cusp Zone 2 | Central Cusp Zone 1 |  |  |  | <.0001* |
| E | Marginal II Cusp Zone 4 | Marginal I Basis Zone 4 |  |  |  | <.0001* |
| E | Lateral Cusp Zone 3 | Lateral Stylus Zone 2 |  |  |  | <.0001* |
| E | Lateral Stylus Zone 4 | Marginal II Cusp Zone 3 |  |  |  | <.0001* |
| E | Lateral Stylus Zone 3 | Marginal II Basis Zone 2 |  |  |  | <.0001* |
| E | Lateral Cusp Zone 4 | Lateral Cusp Zone 3 |  |  |  | <.0001* |
| E | Marginal II Cusp Zone 4 | Marginal II Basis Zone 4 |  |  |  | <.0001* |
| E | Lateral Stylus Zone 3 | Marginal I Basis Zone 2 |  |  |  | <.0001* |
| E | Lateral Stylus Zone 3 | Lateral Cusp Zone 1 |  |  |  | <.0001* |
| E | Central Stylus Zone 2 | Marginal I Cusp Zone 1 |  |  |  | <.0001* |
| E | Central Stylus Zone 2 | Marginal II Cusp Zone 1 |  |  |  | <.0001* |
| E | Central Stylus Zone 2 | Lateral Stylus Zone 1 |  |  |  | <.0001* |
| E | Central Stylus Zone 2 | Marginal I Stylus Zone 1 |  |  |  | <.0001* |
| E | Central Stylus Zone 2 | Marginal II Stylus Zone 1 |  |  |  | <.0001* |
| E | Central Stylus Zone 2 | Marginal II Basis Zone 1 |  |  |  | <.0001* |
| E | Central Stylus Zone 2 | Marginal I Basis Zone 1 |  |  |  | <.0001* |
| E | Lateral Cusp Zone 4 | Marginal I Cusp Zone 4 |  |  |  | <.0001* |
| E | Marginal II Cusp Zone 4 | Lateral Stylus Zone 2 |  |  |  | <.0001* |
| E | Marginal II Stylus Zone 4 | Marginal I Cusp Zone 1 |  |  |  | <.0001* |
| E | Marginal II Stylus Zone 4 | Marginal II Cusp Zone 1 |  |  |  | <.0001* |
| E | Marginal II Stylus Zone 4 | Lateral Stylus Zone 1 |  |  |  | <.0001* |
| E | Marginal II Stylus Zone 4 | Marginal I Stylus Zone 1 |  |  |  | <.0001* |
| E | Marginal II Stylus Zone 4 | Marginal II Stylus Zone 1 |  |  |  | <.0001* |
| E | Marginal II Stylus Zone 4 | Marginal II Basis Zone 1 |  |  |  | <.0001* |
| E | Lateral Stylus Zone 4 | Marginal I Cusp Zone 3 |  |  |  | <.0001* |
| E | Marginal II Stylus Zone 4 | Marginal I Basis Zone 1 |  |  |  | <.0001* |
| E | Lateral Stylus Zone 3 | Marginal I Basis Zone 3 |  |  |  | <.0001* |
| E | Lateral Stylus Zone 3 | Central Stylus Zone 1 |  |  |  | <.0001* |
| E | Marginal I Stylus Zone 4 | Marginal I Cusp Zone 1 |  |  |  | <.0001* |
| E | Marginal I Stylus Zone 4 | Marginal II Cusp Zone 1 |  |  |  | <.0001* |
| E | Lateral Stylus Zone 4 | Lateral Cusp Zone 2 |  |  |  | <.0001* |
| E | Central Cusp Zone 2 | Marginal II Cusp Zone 2 |  |  |  | <.0001* |
| E | Marginal I Stylus Zone 4 | Lateral Stylus Zone 1 |  |  |  | <.0001* |
| E | Marginal I Stylus Zone 4 | Marginal I Stylus Zone 1 |  |  |  | <.0001* |
| E | Marginal I Stylus Zone 4 | Marginal II Stylus Zone 1 |  |  |  | <.0001* |
| E | Lateral Stylus Zone 3 | Marginal II Basis Zone 3 |  |  |  | <.0001* |
| E | Central Stylus Zone 2 | Marginal II Basis Zone 2 |  |  |  | <.0001* |
| E | Marginal I Stylus Zone 4 | Marginal II Basis Zone 1 |  |  |  | <.0001* |
| E | Marginal I Stylus Zone 4 | Marginal I Basis Zone 1 |  |  |  | <.0001* |
| E | Central Stylus Zone 2 | Marginal I Basis Zone 2 |  |  |  | <.0001* |
| E | Lateral Stylus Zone 3 | Marginal I Stylus Zone 2 |  |  |  | <.0001* |
| E | Lateral Stylus Zone 3 | Marginal II Stylus Zone 2 |  |  |  | <.0001* |
| E | Central Stylus Zone 2 | Lateral Cusp Zone 1 |  |  |  | <.0001* |
| E | Marginal II Stylus Zone 4 | Marginal II Basis Zone 2 |  |  |  | <.0001* |
| E | Central Stylus Zone 4 | Lateral Stylus Zone 4 |  |  |  | <.0001* |
| E | Marginal II Stylus Zone 4 | Marginal I Basis Zone 2 |  |  |  | <.0001* |
| E | Central Cusp Zone 2 | Marginal I Cusp Zone 2 |  |  |  | <.0001* |
| E | Lateral Cusp Zone 2 | Marginal I Cusp Zone 1 |  |  |  | <.0001* |
| E | Lateral Cusp Zone 2 | Marginal II Cusp Zone 1 |  |  |  | <.0001* |
| E | Lateral Stylus Zone 4 | Marginal I Stylus Zone 4 |  |  |  | <.0001* |
| E | Marginal I Stylus Zone 4 | Marginal II Basis Zone 2 |  |  |  | <.0001* |
| E | Marginal II Stylus Zone 4 | Lateral Cusp Zone 1 |  |  |  | <.0001* |
| E | Lateral Cusp Zone 2 | Lateral Stylus Zone 1 |  |  |  | <.0001* |
| E | Lateral Cusp Zone 2 | Marginal I Stylus Zone 1 |  |  |  | <.0001* |
| E | Lateral Cusp Zone 2 | Marginal II Stylus Zone 1 |  |  |  | <.0001* |
| E | Lateral Cusp Zone 2 | Marginal II Basis Zone 1 |  |  |  | <.0001* |
| E | Lateral Cusp Zone 2 | Marginal I Basis Zone 1 |  |  |  | <.0001* |
| E | Marginal I Stylus Zone 4 | Marginal I Basis Zone 2 |  |  |  | <.0001* |
| E | Central Cusp Zone 2 | Marginal I Stylus Zone 3 |  |  |  | <.0001* |
| E | Marginal I Cusp Zone 3 | Marginal I Cusp Zone 1 |  |  |  | <.0001* |
| E | Marginal I Cusp Zone 3 | Marginal II Cusp Zone 1 |  |  |  | <.0001* |
| E | Marginal I Cusp Zone 3 | Lateral Stylus Zone 1 |  |  |  | <.0001* |
| E | Marginal I Cusp Zone 3 | Marginal I Stylus Zone 1 |  |  |  | <.0001* |
| E | Marginal I Cusp Zone 3 | Marginal II Stylus Zone 1 |  |  |  | <.0001* |
| E | Central Stylus Zone 2 | Marginal I Basis Zone 3 |  |  |  | <.0001* |
| E | Marginal I Cusp Zone 3 | Marginal II Basis Zone 1 |  |  |  | <.0001* |
| E | Central Stylus Zone 2 | Central Stylus Zone 1 |  |  |  | <.0001* |
| E | Lateral Stylus Zone 4 | Marginal II Stylus Zone 4 |  |  |  | <.0001* |
| E | Marginal I Cusp Zone 3 | Marginal I Basis Zone 1 |  |  |  | <.0001* |
| E | Marginal I Stylus Zone 4 | Lateral Cusp Zone 1 |  |  |  | <.0001* |
| E | Central Cusp Zone 2 | Marginal II Stylus Zone 3 |  |  |  | <.0001* |
| E | Lateral Cusp Zone 4 | Central Stylus Zone 3 |  |  |  | <.0001* |
| E | Central Stylus Zone 2 | Marginal II Basis Zone 3 |  |  |  | <.0001* |
| E | Central Stylus Zone 2 | Marginal I Stylus Zone 2 |  |  |  | <.0001* |
| E | Central Cusp Zone 2 | Marginal I Basis Zone 4 |  |  |  | <.0001* |
| E | Central Stylus Zone 3 | Marginal II Cusp Zone 3 |  |  |  | <.0001* |
| E | Central Stylus Zone 2 | Marginal II Stylus Zone 2 |  |  |  | <.0001* |
| E | Marginal II Stylus Zone 4 | Marginal I Basis Zone 3 |  |  |  | <.0001* |
| E | Marginal II Stylus Zone 4 | Central Stylus Zone 1 |  |  |  | <.0001* |
| E | Lateral Stylus Zone 4 | Central Stylus Zone 2 |  |  |  | <.0001* |
| E | Lateral Cusp Zone 2 | Marginal II Basis Zone 2 |  |  |  | <.0001* |
| E | Lateral Cusp Zone 2 | Marginal I Basis Zone 2 |  |  |  | <.0001* |
| E | Marginal II Stylus Zone 4 | Marginal II Basis Zone 3 |  |  |  | <.0001* |
| E | Central Cusp Zone 4 | Lateral Cusp Zone 4 |  |  |  | <.0001* |
| E | Marginal I Stylus Zone 4 | Marginal I Basis Zone 3 |  |  |  | <.0001* |
| E | Marginal I Cusp Zone 3 | Marginal II Basis Zone 2 |  |  |  | <.0001* |
| E | Marginal II Stylus Zone 4 | Marginal I Stylus Zone 2 |  |  |  | <.0001* |
| E | Marginal I Stylus Zone 4 | Central Stylus Zone 1 |  |  |  | <.0001* |
| E | Central Cusp Zone 3 | Central Cusp Zone 2 |  |  |  | <.0001* |
| E | Marginal II Cusp Zone 3 | Marginal I Cusp Zone 1 |  |  |  | <.0001* |
| E | Marginal I Cusp Zone 3 | Marginal I Basis Zone 2 |  |  |  | <.0001* |
| E | Marginal II Cusp Zone 3 | Marginal II Cusp Zone 1 |  |  |  | <.0001* |
| E | Lateral Cusp Zone 2 | Lateral Cusp Zone 1 |  |  |  | <.0001* |
| E | Marginal II Stylus Zone 4 | Marginal II Stylus Zone 2 |  |  |  | <.0001* |
| E | Marginal II Cusp Zone 3 | Lateral Stylus Zone 1 |  |  |  | <.0001* |
| E | Marginal II Cusp Zone 3 | Marginal I Stylus Zone 1 |  |  |  | <.0001* |
| E | Marginal II Cusp Zone 3 | Marginal II Stylus Zone 1 |  |  |  | <.0001* |
| E | Marginal II Cusp Zone 3 | Marginal II Basis Zone 1 |  |  |  | <.0001* |
| E | Marginal I Stylus Zone 4 | Marginal II Basis Zone 3 |  |  |  | <.0001* |
| E | Marginal II Cusp Zone 3 | Marginal I Basis Zone 1 |  |  |  | <.0001* |
| E | Marginal I Cusp Zone 3 | Lateral Cusp Zone 1 |  |  |  | <.0001* |
| E | Marginal I Stylus Zone 4 | Marginal I Stylus Zone 2 |  |  |  | <.0001* |
| E | Central Cusp Zone 2 | Marginal II Basis Zone 4 |  |  |  | <.0001* |
| E | Marginal I Stylus Zone 4 | Marginal II Stylus Zone 2 |  |  |  | <.0001* |
| E | Central Stylus Zone 3 | Marginal I Cusp Zone 3 |  |  |  | <.0001* |
| E | Central Stylus Zone 3 | Lateral Cusp Zone 2 |  |  |  | <.0001* |
| E | Lateral Cusp Zone 2 | Marginal I Basis Zone 3 |  |  |  | <.0001* |
| E | Lateral Cusp Zone 2 | Central Stylus Zone 1 |  |  |  | <.0001* |
| E | Lateral Stylus Zone 3 | Central Cusp Zone 1 |  |  |  | <.0001* |
| E | Lateral Stylus Zone 4 | Lateral Stylus Zone 3 |  |  |  | <.0001* |
| E | Marginal II Cusp Zone 3 | Marginal II Basis Zone 2 |  |  |  | <.0001* |
| E | Central Cusp Zone 2 | Lateral Stylus Zone 2 |  |  |  | <.0001* |
| E | Marginal I Cusp Zone 3 | Marginal I Basis Zone 3 |  |  |  | <.0001* |
| E | Lateral Cusp Zone 2 | Marginal II Basis Zone 3 |  |  |  | <.0001* |
| E | Marginal I Cusp Zone 3 | Central Stylus Zone 1 |  |  |  | <.0001* |
| E | Marginal II Cusp Zone 3 | Marginal I Basis Zone 2 |  |  |  | <.0001* |
| E | Lateral Cusp Zone 2 | Marginal I Stylus Zone 2 |  |  |  | <.0001* |
| E | Marginal I Cusp Zone 3 | Marginal II Basis Zone 3 |  |  |  | <.0001* |
| E | Marginal II Cusp Zone 3 | Lateral Cusp Zone 1 |  |  |  | <.0001* |
| E | Lateral Cusp Zone 2 | Marginal II Stylus Zone 2 |  |  |  | <.0001* |
| E | Marginal I Cusp Zone 4 | Marginal II Cusp Zone 3 |  |  |  | <.0001* |
| E | Marginal I Cusp Zone 3 | Marginal I Stylus Zone 2 |  |  |  | <.0001* |
| E | Marginal I Cusp Zone 3 | Marginal II Stylus Zone 2 |  |  |  | <.0001* |
| E | Central Stylus Zone 3 | Marginal I Stylus Zone 4 |  |  |  | <.0001* |
| E | Central Stylus Zone 3 | Marginal II Stylus Zone 4 |  |  |  | <.0001* |
| E | Lateral Cusp Zone 3 | Marginal II Cusp Zone 3 |  |  |  | <.0001* |
| E | Lateral Cusp Zone 4 | Lateral Stylus Zone 4 |  |  |  | <.0001* |
| E | Marginal II Cusp Zone 3 | Marginal I Basis Zone 3 |  |  |  | <.0001* |
| E | Marginal II Cusp Zone 3 | Central Stylus Zone 1 |  |  |  | <.0001* |
| E | Central Stylus Zone 2 | Central Cusp Zone 1 |  |  |  | <.0001* |
| E | Marginal II Cusp Zone 3 | Marginal II Basis Zone 3 |  |  |  | <.0001* |
| E | Marginal I Cusp Zone 4 | Marginal I Cusp Zone 3 |  |  |  | <.0001* |
| E | Marginal II Cusp Zone 3 | Marginal I Stylus Zone 2 |  |  |  | <.0001* |
| E | Central Stylus Zone 3 | Central Stylus Zone 2 |  |  |  | <.0001* |
| E | Marginal II Cusp Zone 3 | Marginal II Stylus Zone 2 |  |  |  | <.0001* |
| E | Marginal I Cusp Zone 4 | Lateral Cusp Zone 2 |  |  |  | <.0001* |
| E | Marginal II Stylus Zone 4 | Central Cusp Zone 1 |  |  |  | <.0001* |
| E | Lateral Stylus Zone 3 | Marginal II Cusp Zone 2 |  |  |  | <.0001* |
| E | Lateral Cusp Zone 3 | Marginal I Cusp Zone 3 |  |  |  | <.0001* |
| E | Marginal I Stylus Zone 4 | Central Cusp Zone 1 |  |  |  | <.0001* |
| E | Lateral Cusp Zone 3 | Lateral Cusp Zone 2 |  |  |  | <.0001* |
| E | Marginal II Cusp Zone 4 | Marginal II Cusp Zone 3 |  |  |  | <.0001* |
| E | Central Cusp Zone 4 | Central Stylus Zone 4 |  |  |  | <.0001* |
| E | Marginal I Cusp Zone 4 | Marginal I Stylus Zone 4 |  |  |  | <.0001* |
| E | Central Cusp Zone 3 | Marginal II Cusp Zone 4 |  |  |  | <.0001* |
| E | Lateral Stylus Zone 3 | Marginal I Cusp Zone 2 |  |  |  | <.0001* |
| E | Marginal I Cusp Zone 4 | Marginal II Stylus Zone 4 |  |  |  | <.0001* |
| E | Central Stylus Zone 3 | Lateral Stylus Zone 3 |  |  |  | <.0001* |
| E | Lateral Stylus Zone 3 | Marginal I Stylus Zone 3 |  |  |  | <.0001* |
| E | Lateral Cusp Zone 2 | Central Cusp Zone 1 |  |  |  | <.0001* |
| E | Lateral Cusp Zone 3 | Marginal I Stylus Zone 4 |  |  |  | <.0001* |
| E | Lateral Stylus Zone 3 | Marginal II Stylus Zone 3 |  |  |  | <.0001* |
| E | Marginal I Cusp Zone 3 | Central Cusp Zone 1 |  |  |  | <.0001* |
| E | Central Stylus Zone 2 | Marginal II Cusp Zone 2 |  |  |  | <.0001* |
| E | Marginal I Cusp Zone 4 | Central Stylus Zone 2 |  |  |  | <.0001* |
| E | Marginal II Cusp Zone 4 | Marginal I Cusp Zone 3 |  |  |  | <.0001* |
| E | Lateral Cusp Zone 3 | Marginal II Stylus Zone 4 |  |  |  | <.0001* |
| E | Lateral Stylus Zone 3 | Marginal I Basis Zone 4 |  |  |  | <.0001* |
| E | Marginal II Cusp Zone 4 | Lateral Cusp Zone 2 |  |  |  | <.0001* |
| E | Central Stylus Zone 4 | Central Cusp Zone 3 |  |  |  | <.0001* |
| E | Central Cusp Zone 3 | Lateral Cusp Zone 3 |  |  |  | <.0001* |
| E | Marginal II Stylus Zone 4 | Marginal II Cusp Zone 2 |  |  |  | <.0001* |
| E | Lateral Cusp Zone 3 | Central Stylus Zone 2 |  |  |  | <.0001* |
| E | Central Stylus Zone 2 | Marginal I Cusp Zone 2 |  |  |  | <.0001* |
| E | Marginal I Stylus Zone 4 | Marginal II Cusp Zone 2 |  |  |  | <.0001* |
| E | Marginal II Cusp Zone 3 | Central Cusp Zone 1 |  |  |  | <.0001* |
| E | Lateral Stylus Zone 3 | Marginal II Basis Zone 4 |  |  |  | <.0001* |
| E | Central Stylus Zone 2 | Marginal I Stylus Zone 3 |  |  |  | <.0001* |
| E | Marginal II Cusp Zone 4 | Marginal I Stylus Zone 4 |  |  |  | <.0001* |
| E | Central Cusp Zone 3 | Marginal I Cusp Zone 4 |  |  |  | <.0001* |
| E | Central Stylus Zone 2 | Marginal II Stylus Zone 3 |  |  |  | <.0001* |
| E | Marginal II Stylus Zone 4 | Marginal I Cusp Zone 2 |  |  |  | <.0001* |
| E | Marginal I Cusp Zone 4 | Lateral Stylus Zone 3 |  |  |  | <.0001* |
| E | Marginal II Cusp Zone 4 | Marginal II Stylus Zone 4 |  |  |  | <.0001* |
| E | Central Stylus Zone 2 | Marginal I Basis Zone 4 |  |  |  | <.0001* |
| E | Lateral Stylus Zone 2 | Marginal I Cusp Zone 1 |  |  |  | <.0001* |
| E | Lateral Stylus Zone 2 | Marginal II Cusp Zone 1 |  |  |  | <.0001* |
| E | Marginal I Stylus Zone 4 | Marginal I Cusp Zone 2 |  |  |  | <.0001* |
| E | Marginal II Stylus Zone 4 | Marginal I Stylus Zone 3 |  |  |  | <.0001* |
| E | Lateral Stylus Zone 2 | Lateral Stylus Zone 1 |  |  |  | <.0001* |
| E | Lateral Stylus Zone 2 | Marginal I Stylus Zone 1 |  |  |  | <.0001* |
| E | Lateral Stylus Zone 2 | Marginal II Stylus Zone 1 |  |  |  | <.0001* |
| E | Lateral Stylus Zone 2 | Marginal II Basis Zone 1 |  |  |  | <.0001* |
| E | Lateral Stylus Zone 2 | Marginal I Basis Zone 1 |  |  |  | <.0001* |
| E | Lateral Stylus Zone 4 | Central Cusp Zone 2 |  |  |  | <.0001* |
| E | Lateral Stylus Zone 3 | Lateral Stylus Zone 2 |  |  |  | <.0001* |
| E | Lateral Cusp Zone 2 | Marginal II Cusp Zone 2 |  |  |  | <.0001* |
| E | Marginal II Stylus Zone 4 | Marginal II Stylus Zone 3 |  |  |  | <.0001* |
| E | Marginal I Stylus Zone 4 | Marginal I Stylus Zone 3 |  |  |  | <.0001* |
| E | Marginal I Cusp Zone 3 | Marginal II Cusp Zone 2 |  |  |  | <.0001* |
| E | Lateral Cusp Zone 3 | Lateral Stylus Zone 3 |  |  |  | <.0001* |
| E | Marginal II Cusp Zone 4 | Central Stylus Zone 2 |  |  |  | <.0001* |
| E | Marginal II Stylus Zone 4 | Marginal I Basis Zone 4 |  |  |  | <.0001* |
| E | Marginal I Stylus Zone 4 | Marginal II Stylus Zone 3 |  |  |  | <.0001* |
| E | Marginal II Basis Zone 4 | Marginal I Cusp Zone 1 |  |  |  | <.0001* |
| E | Marginal II Basis Zone 4 | Marginal II Cusp Zone 1 |  |  |  | <.0001* |
| E | Central Stylus Zone 2 | Marginal II Basis Zone 4 |  |  |  | <.0001* |
| E | Marginal II Basis Zone 4 | Lateral Stylus Zone 1 |  |  |  | <.0001* |
| E | Marginal II Basis Zone 4 | Marginal I Stylus Zone 1 |  |  |  | <.0001* |
| E | Marginal II Basis Zone 4 | Marginal II Stylus Zone 1 |  |  |  | <.0001* |
| E | Marginal I Stylus Zone 4 | Marginal I Basis Zone 4 |  |  |  | <.0001* |
| E | Marginal II Basis Zone 4 | Marginal II Basis Zone 1 |  |  |  | <.0001* |
| E | Marginal II Basis Zone 4 | Marginal I Basis Zone 1 |  |  |  | <.0001* |
| E | Lateral Cusp Zone 2 | Marginal I Cusp Zone 2 |  |  |  | <.0001* |
| E | Lateral Stylus Zone 2 | Marginal II Basis Zone 2 |  |  |  | <.0001* |
| E | Central Cusp Zone 2 | Marginal II Cusp Zone 3 |  |  |  | <.0001* |
| E | Lateral Stylus Zone 2 | Marginal I Basis Zone 2 |  |  |  | <.0001* |
| E | Marginal I Cusp Zone 3 | Marginal I Cusp Zone 2 |  |  |  | <.0001* |
| E | Lateral Cusp Zone 2 | Marginal I Stylus Zone 3 |  |  |  | <.0001* |
| E | Lateral Stylus Zone 2 | Lateral Cusp Zone 1 |  |  |  | <.0001* |
| E | Marginal II Stylus Zone 4 | Marginal II Basis Zone 4 |  |  |  | <.0001* |
| E | Central Cusp Zone 3 | Central Stylus Zone 3 |  |  |  | <.0001* |
| E | Marginal I Cusp Zone 3 | Marginal I Stylus Zone 3 |  |  |  | <.0001* |
| E | Marginal II Cusp Zone 3 | Marginal II Cusp Zone 2 |  |  |  | <.0001* |
| E | Lateral Cusp Zone 2 | Marginal II Stylus Zone 3 |  |  |  | <.0001* |
| E | Central Stylus Zone 2 | Lateral Stylus Zone 2 |  |  |  | <.0001* |
| E | Marginal I Basis Zone 4 | Marginal I Cusp Zone 1 |  |  |  | <.0001* |
| E | Marginal I Basis Zone 4 | Marginal II Cusp Zone 1 |  |  |  | <.0001* |
| E | Marginal I Basis Zone 4 | Lateral Stylus Zone 1 |  |  |  | <.0001* |
| E | Marginal I Basis Zone 4 | Marginal I Stylus Zone 1 |  |  |  | <.0001* |
| E | Marginal I Basis Zone 4 | Marginal II Stylus Zone 1 |  |  |  | <.0001* |
| E | Marginal I Stylus Zone 4 | Marginal II Basis Zone 4 |  |  |  | <.0001* |
| E | Marginal I Cusp Zone 3 | Marginal II Stylus Zone 3 |  |  |  | <.0001* |
| E | Marginal I Basis Zone 4 | Marginal II Basis Zone 1 |  |  |  | <.0001* |
| E | Marginal I Basis Zone 4 | Marginal I Basis Zone 1 |  |  |  | <.0001* |
| E | Marginal II Basis Zone 4 | Marginal II Basis Zone 2 |  |  |  | <.0001* |
| E | Lateral Cusp Zone 2 | Marginal I Basis Zone 4 |  |  |  | <.0001* |
| E | Marginal II Cusp Zone 4 | Lateral Stylus Zone 3 |  |  |  | <.0001* |
| E | Marginal II Basis Zone 4 | Marginal I Basis Zone 2 |  |  |  | <.0001* |
| E | Marginal II Stylus Zone 3 | Marginal I Cusp Zone 1 |  |  |  | <.0001* |
| E | Marginal II Stylus Zone 3 | Marginal II Cusp Zone 1 |  |  |  | <.0001* |
| E | Marginal II Stylus Zone 3 | Lateral Stylus Zone 1 |  |  |  | <.0001* |
| E | Marginal II Stylus Zone 3 | Marginal I Stylus Zone 1 |  |  |  | <.0001* |
| E | Marginal II Stylus Zone 3 | Marginal II Stylus Zone 1 |  |  |  | <.0001* |
| E | Marginal I Cusp Zone 3 | Marginal I Basis Zone 4 |  |  |  | <.0001* |
| E | Marginal II Stylus Zone 3 | Marginal II Basis Zone 1 |  |  |  | <.0001* |
| E | Marginal II Stylus Zone 3 | Marginal I Basis Zone 1 |  |  |  | <.0001* |
| E | Marginal II Basis Zone 4 | Lateral Cusp Zone 1 |  |  |  | <.0001* |
| E | Central Cusp Zone 2 | Marginal I Cusp Zone 3 |  |  |  | <.0001* |
| E | Marginal I Stylus Zone 3 | Marginal I Cusp Zone 1 |  |  |  | <.0001* |
| E | Marginal I Stylus Zone 3 | Marginal II Cusp Zone 1 |  |  |  | <.0001* |
| E | Lateral Stylus Zone 2 | Marginal I Basis Zone 3 |  |  |  | <.0001* |
| E | Marginal II Stylus Zone 4 | Lateral Stylus Zone 2 |  |  |  | <.0001* |
| E | Lateral Stylus Zone 2 | Central Stylus Zone 1 |  |  |  | <.0001* |
| E | Marginal I Stylus Zone 3 | Lateral Stylus Zone 1 |  |  |  | <.0001* |
| E | Marginal I Stylus Zone 3 | Marginal I Stylus Zone 1 |  |  |  | <.0001* |
| E | Marginal I Stylus Zone 3 | Marginal II Stylus Zone 1 |  |  |  | <.0001* |
| E | Marginal I Stylus Zone 3 | Marginal II Basis Zone 1 |  |  |  | <.0001* |
| E | Marginal I Stylus Zone 3 | Marginal I Basis Zone 1 |  |  |  | <.0001* |
| E | Marginal II Cusp Zone 3 | Marginal I Cusp Zone 2 |  |  |  | <.0001* |
| E | Central Cusp Zone 2 | Lateral Cusp Zone 2 |  |  |  | <.0001* |
| E | Lateral Stylus Zone 2 | Marginal II Basis Zone 3 |  |  |  | <.0001* |
| E | Lateral Cusp Zone 4 | Central Cusp Zone 3 |  |  |  | <.0001* |
| E | Marginal I Cusp Zone 2 | Marginal I Cusp Zone 1 |  |  |  | <.0001* |
| E | Marginal I Cusp Zone 2 | Marginal II Cusp Zone 1 |  |  |  | <.0001* |
| E | Lateral Stylus Zone 2 | Marginal I Stylus Zone 2 |  |  |  | <.0001* |
| E | Marginal I Stylus Zone 4 | Lateral Stylus Zone 2 |  |  |  | <.0001* |
| E | Marginal I Cusp Zone 2 | Lateral Stylus Zone 1 |  |  |  | <.0001* |
| E | Marginal I Cusp Zone 2 | Marginal I Stylus Zone 1 |  |  |  | <.0001* |
| E | Marginal I Cusp Zone 2 | Marginal II Stylus Zone 1 |  |  |  | <.0001* |
| E | Marginal I Cusp Zone 2 | Marginal II Basis Zone 1 |  |  |  | <.0001* |
| E | Marginal I Cusp Zone 2 | Marginal I Basis Zone 1 |  |  |  | <.0001* |
| E | Marginal II Cusp Zone 3 | Marginal I Stylus Zone 3 |  |  |  | <.0001* |
| E | Marginal I Basis Zone 4 | Marginal II Basis Zone 2 |  |  |  | <.0001* |
| E | Lateral Stylus Zone 2 | Marginal II Stylus Zone 2 |  |  |  | <.0001* |
| E | Lateral Cusp Zone 2 | Marginal II Basis Zone 4 |  |  |  | <.0001* |
| E | Marginal I Basis Zone 4 | Marginal I Basis Zone 2 |  |  |  | <.0001* |
| E | Marginal II Cusp Zone 3 | Marginal II Stylus Zone 3 |  |  |  | <.0001* |
| E | Marginal I Cusp Zone 3 | Marginal II Basis Zone 4 |  |  |  | <.0001* |
| E | Marginal II Stylus Zone 3 | Marginal II Basis Zone 2 |  |  |  | <.0001* |
| E | Marginal II Basis Zone 4 | Marginal I Basis Zone 3 |  |  |  | <.0001* |
| E | Marginal II Basis Zone 4 | Central Stylus Zone 1 |  |  |  | <.0001* |
| E | Marginal I Basis Zone 4 | Lateral Cusp Zone 1 |  |  |  | <.0001* |
| E | Marginal II Stylus Zone 3 | Marginal I Basis Zone 2 |  |  |  | <.0001* |
| E | Marginal II Cusp Zone 3 | Marginal I Basis Zone 4 |  |  |  | <.0001* |
| E | Marginal II Basis Zone 4 | Marginal II Basis Zone 3 |  |  |  | <.0001* |
| E | Marginal I Stylus Zone 3 | Marginal II Basis Zone 2 |  |  |  | <.0001* |
| E | Marginal II Cusp Zone 2 | Marginal I Cusp Zone 1 |  |  |  | <.0001* |
| E | Central Stylus Zone 3 | Central Cusp Zone 2 |  |  |  | <.0001* |
| E | Marginal II Cusp Zone 2 | Marginal II Cusp Zone 1 |  |  |  | <.0001* |
| E | Marginal I Stylus Zone 3 | Marginal I Basis Zone 2 |  |  |  | <.0001* |
| E | Marginal II Stylus Zone 3 | Lateral Cusp Zone 1 |  |  |  | <.0001* |
| E | Marginal II Basis Zone 4 | Marginal I Stylus Zone 2 |  |  |  | <.0001* |
| E | Central Cusp Zone 2 | Marginal I Stylus Zone 4 |  |  |  | <.0001* |
| E | Marginal II Cusp Zone 2 | Lateral Stylus Zone 1 |  |  |  | <.0001* |
| E | Marginal II Cusp Zone 2 | Marginal I Stylus Zone 1 |  |  |  | <.0001* |
| E | Marginal II Cusp Zone 2 | Marginal II Stylus Zone 1 |  |  |  | <.0001* |
| E | Marginal II Cusp Zone 2 | Marginal II Basis Zone 1 |  |  |  | <.0001* |
| E | Marginal II Cusp Zone 2 | Marginal I Basis Zone 1 |  |  |  | <.0001* |
| E | Marginal II Basis Zone 4 | Marginal II Stylus Zone 2 |  |  |  | <.0001* |
| E | Lateral Cusp Zone 2 | Lateral Stylus Zone 2 |  |  |  | <.0001* |
| E | Marginal I Cusp Zone 2 | Marginal II Basis Zone 2 |  |  |  | <.0001* |
| E | Marginal I Stylus Zone 3 | Lateral Cusp Zone 1 |  |  |  | <.0001* |
| E | Marginal I Cusp Zone 2 | Marginal I Basis Zone 2 |  |  |  | <.0001* |
| E | Central Cusp Zone 2 | Marginal II Stylus Zone 4 |  |  |  | <.0001* |
| E | Marginal I Cusp Zone 3 | Lateral Stylus Zone 2 |  |  |  | <.0001* |
| E | Marginal I Cusp Zone 2 | Lateral Cusp Zone 1 |  |  |  | <.0001* |
| E | Marginal I Basis Zone 4 | Marginal I Basis Zone 3 |  |  |  | <.0001* |
| E | Marginal I Basis Zone 4 | Central Stylus Zone 1 |  |  |  | <.0001* |
| E | Lateral Stylus Zone 4 | Marginal II Cusp Zone 4 |  |  |  | <.0001* |
| E | Marginal II Cusp Zone 3 | Marginal II Basis Zone 4 |  |  |  | <.0001* |
| E | Marginal I Basis Zone 4 | Marginal II Basis Zone 3 |  |  |  | <.0001* |
| E | Marginal II Stylus Zone 3 | Marginal I Basis Zone 3 |  |  |  | <.0001* |
| E | Marginal II Stylus Zone 3 | Central Stylus Zone 1 |  |  |  | <.0001* |
| E | Marginal I Basis Zone 4 | Marginal I Stylus Zone 2 |  |  |  | <.0001* |
| E | Central Cusp Zone 2 | Central Stylus Zone 2 |  |  |  | <.0001* |
| E | Marginal II Cusp Zone 2 | Marginal II Basis Zone 2 |  |  |  | <.0001* |
| E | Marginal I Basis Zone 4 | Marginal II Stylus Zone 2 |  |  |  | <.0001* |
| E | Marginal II Stylus Zone 3 | Marginal II Basis Zone 3 |  |  |  | <.0001* |
| E | Marginal I Stylus Zone 3 | Marginal I Basis Zone 3 |  |  |  | <.0001* |
| E | Marginal II Cusp Zone 2 | Marginal I Basis Zone 2 |  |  |  | <.0001* |
| E | Marginal I Stylus Zone 3 | Central Stylus Zone 1 |  |  |  | <.0001* |
| E | Marginal II Stylus Zone 3 | Marginal I Stylus Zone 2 |  |  |  | <.0001* |
| E | Central Cusp Zone 3 | Lateral Stylus Zone 4 |  |  |  | <.0001* |
| E | Marginal I Stylus Zone 3 | Marginal II Basis Zone 3 |  |  |  | <.0001* |
| E | Marginal II Cusp Zone 2 | Lateral Cusp Zone 1 |  |  |  | <.0001* |
| E | Marginal II Stylus Zone 3 | Marginal II Stylus Zone 2 |  |  |  | <.0001* |
| E | Marginal I Cusp Zone 2 | Marginal I Basis Zone 3 |  |  |  | <.0001* |
| E | Marginal I Stylus Zone 3 | Marginal I Stylus Zone 2 |  |  |  | <.0001* |
| E | Marginal I Cusp Zone 2 | Central Stylus Zone 1 |  |  |  | <.0001* |
| E | Marginal II Cusp Zone 3 | Lateral Stylus Zone 2 |  |  |  | <.0001* |
| E | Marginal I Stylus Zone 3 | Marginal II Stylus Zone 2 |  |  |  | <.0001* |
| E | Marginal I Cusp Zone 2 | Marginal II Basis Zone 3 |  |  |  | <.0001* |
| E | Marginal I Cusp Zone 2 | Marginal I Stylus Zone 2 |  |  |  | <.0001* |
| E | Marginal I Cusp Zone 4 | Central Cusp Zone 2 |  |  |  | <.0001* |
| E | Marginal I Cusp Zone 2 | Marginal II Stylus Zone 2 |  |  |  | <.0001* |
| E | Lateral Stylus Zone 4 | Lateral Cusp Zone 3 |  |  |  | <.0001* |
| E | Central Cusp Zone 1 | Marginal I Cusp Zone 1 |  |  |  | <.0001* |
| E | Central Cusp Zone 1 | Marginal II Cusp Zone 1 |  |  |  | <.0001* |
| E | Marginal II Cusp Zone 2 | Marginal I Basis Zone 3 |  |  |  | <.0001* |
| E | Marginal II Cusp Zone 2 | Central Stylus Zone 1 |  |  |  | <.0001* |
| E | Central Cusp Zone 1 | Lateral Stylus Zone 1 |  |  |  | <.0001* |
| E | Central Cusp Zone 1 | Marginal I Stylus Zone 1 |  |  |  | <.0001* |
| E | Central Cusp Zone 1 | Marginal II Stylus Zone 1 |  |  |  | <.0001* |
| E | Central Cusp Zone 1 | Marginal II Basis Zone 1 |  |  |  | <.0001* |
| E | Central Cusp Zone 1 | Marginal I Basis Zone 1 |  |  |  | <.0001* |
| E | Lateral Stylus Zone 2 | Central Cusp Zone 1 |  |  |  | <.0001* |
| E | Central Cusp Zone 2 | Lateral Stylus Zone 3 |  |  |  | <.0001* |
| E | Marginal II Cusp Zone 2 | Marginal II Basis Zone 3 |  |  |  | <.0001* |
| E | Marginal II Cusp Zone 2 | Marginal I Stylus Zone 2 |  |  |  | <.0001* |
| E | Lateral Cusp Zone 3 | Central Cusp Zone 2 |  |  |  | <.0001* |
| E | Marginal II Cusp Zone 2 | Marginal II Stylus Zone 2 |  |  |  | <.0001* |
| E | Lateral Stylus Zone 4 | Marginal I Cusp Zone 4 |  |  |  | <.0001* |
| E | Marginal II Basis Zone 4 | Central Cusp Zone 1 |  |  |  | <.0001* |
| E | Lateral Stylus Zone 3 | Marginal II Cusp Zone 3 |  |  |  | <.0001* |
| E | Central Cusp Zone 1 | Marginal II Basis Zone 2 |  |  |  | <.0001* |
| E | Central Cusp Zone 1 | Marginal I Basis Zone 2 |  |  |  | <.0001* |
| E | Central Cusp Zone 1 | Lateral Cusp Zone 1 |  |  |  | <.0001* |
| E | Central Stylus Zone 3 | Marginal II Cusp Zone 4 |  |  |  | <.0001* |
| E | Marginal I Basis Zone 4 | Central Cusp Zone 1 |  |  |  | <.0001* |
| E | Central Stylus Zone 4 | Lateral Cusp Zone 4 |  |  |  | <.0001* |
| E | Marginal II Cusp Zone 4 | Central Cusp Zone 2 |  |  |  | <.0001* |
| E | Lateral Stylus Zone 3 | Marginal I Cusp Zone 3 |  |  |  | <.0001* |
| E | Marginal II Stylus Zone 3 | Central Cusp Zone 1 |  |  |  | <.0001* |
| E | Central Cusp Zone 1 | Marginal I Basis Zone 3 |  |  |  | <.0001* |
| E | Central Cusp Zone 1 | Central Stylus Zone 1 |  |  |  | <.0001* |
| E | Lateral Stylus Zone 3 | Lateral Cusp Zone 2 |  |  |  | <.0001* |
| E | Lateral Stylus Zone 2 | Marginal II Cusp Zone 2 |  |  |  | <.0001* |
| E | Marginal I Stylus Zone 3 | Central Cusp Zone 1 |  |  |  | <.0001* |
| E | Central Stylus Zone 2 | Marginal II Cusp Zone 3 |  |  |  | <.0001* |
| E | Central Cusp Zone 1 | Marginal II Basis Zone 3 |  |  |  | <.0001* |
| E | Central Cusp Zone 1 | Marginal I Stylus Zone 2 |  |  |  | <.0001* |
| E | Lateral Stylus Zone 4 | Central Stylus Zone 3 |  |  |  | <.0001* |
| E | Marginal I Cusp Zone 2 | Central Cusp Zone 1 |  |  |  | <.0001* |
| E | Central Cusp Zone 1 | Marginal II Stylus Zone 2 |  |  |  | <.0001* |
| E | Marginal II Stylus Zone 2 | Marginal I Cusp Zone 1 |  |  |  | <.0001* |
| E | Marginal II Stylus Zone 2 | Marginal II Cusp Zone 1 |  |  |  | <.0001* |
| E | Central Stylus Zone 3 | Lateral Cusp Zone 3 |  |  |  | <.0001* |
| E | Marginal II Stylus Zone 4 | Marginal II Cusp Zone 3 |  |  |  | <.0001* |
| E | Marginal II Stylus Zone 2 | Lateral Stylus Zone 1 |  |  |  | <.0001* |
| E | Marginal II Stylus Zone 2 | Marginal I Stylus Zone 1 |  |  |  | <.0001* |
| E | Marginal II Stylus Zone 2 | Marginal II Stylus Zone 1 |  |  |  | <.0001* |
| E | Marginal II Stylus Zone 2 | Marginal II Basis Zone 1 |  |  |  | <.0001* |
| E | Marginal II Stylus Zone 2 | Marginal I Basis Zone 1 |  |  |  | <.0001* |
| E | Marginal I Stylus Zone 2 | Marginal I Cusp Zone 1 |  |  |  | <.0001* |
| E | Marginal I Stylus Zone 2 | Marginal II Cusp Zone 1 |  |  |  | <.0001* |
| E | Lateral Stylus Zone 2 | Marginal I Cusp Zone 2 |  |  |  | <.0001* |
| E | Marginal II Basis Zone 4 | Marginal II Cusp Zone 2 |  |  |  | <.0001* |
| E | Marginal I Stylus Zone 2 | Lateral Stylus Zone 1 |  |  |  | <.0001* |
| E | Marginal I Stylus Zone 2 | Marginal I Stylus Zone 1 |  |  |  | <.0001* |
| E | Marginal I Stylus Zone 2 | Marginal II Stylus Zone 1 |  |  |  | <.0001* |
| E | Marginal II Basis Zone 3 | Marginal I Cusp Zone 1 |  |  |  | <.0001* |
| E | Marginal I Stylus Zone 2 | Marginal II Basis Zone 1 |  |  |  | <.0001* |
| E | Marginal II Basis Zone 3 | Marginal II Cusp Zone 1 |  |  |  | <.0001* |
| E | Marginal I Stylus Zone 2 | Marginal I Basis Zone 1 |  |  |  | <.0001* |
| E | Marginal II Basis Zone 3 | Lateral Stylus Zone 1 |  |  |  | <.0001* |
| E | Marginal II Basis Zone 3 | Marginal I Stylus Zone 1 |  |  |  | <.0001* |
| E | Marginal II Basis Zone 3 | Marginal II Stylus Zone 1 |  |  |  | <.0001* |
| E | Lateral Stylus Zone 3 | Marginal I Stylus Zone 4 |  |  |  | <.0001* |
| E | Marginal II Basis Zone 3 | Marginal II Basis Zone 1 |  |  |  | <.0001* |
| E | Marginal I Stylus Zone 4 | Marginal II Cusp Zone 3 |  |  |  | <.0001* |
| E | Marginal II Basis Zone 3 | Marginal I Basis Zone 1 |  |  |  | <.0001* |
| E | Central Stylus Zone 1 | Marginal I Cusp Zone 1 |  |  |  | <.0001* |
| E | Central Stylus Zone 1 | Marginal II Cusp Zone 1 |  |  |  | <.0001* |
| E | Marginal I Basis Zone 3 | Marginal I Cusp Zone 1 |  |  |  | <.0001* |
| E | Marginal I Basis Zone 3 | Marginal II Cusp Zone 1 |  |  |  | <.0001* |
| E | Lateral Stylus Zone 2 | Marginal I Stylus Zone 3 |  |  |  | <.0001* |
| E | Marginal II Cusp Zone 2 | Central Cusp Zone 1 |  |  |  | <.0001* |
| E | Central Stylus Zone 1 | Lateral Stylus Zone 1 |  |  |  | <.0001* |
| E | Central Stylus Zone 1 | Marginal I Stylus Zone 1 |  |  |  | <.0001* |
| E | Central Stylus Zone 1 | Marginal II Stylus Zone 1 |  |  |  | <.0001* |
| E | Marginal I Basis Zone 3 | Lateral Stylus Zone 1 |  |  |  | <.0001* |
| E | Marginal I Cusp Zone 4 | Marginal II Cusp Zone 4 |  |  |  | <.0001* |
| E | Central Stylus Zone 1 | Marginal II Basis Zone 1 |  |  |  | <.0001* |
| E | Marginal I Basis Zone 3 | Marginal I Stylus Zone 1 |  |  |  | <.0001* |
| E | Marginal I Basis Zone 3 | Marginal II Stylus Zone 1 |  |  |  | <.0001* |
| E | Central Stylus Zone 2 | Marginal I Cusp Zone 3 |  |  |  | <.0001* |
| E | Central Stylus Zone 1 | Marginal I Basis Zone 1 |  |  |  | <.0001* |
| E | Marginal I Basis Zone 3 | Marginal II Basis Zone 1 |  |  |  | <.0001* |
| E | Marginal I Basis Zone 3 | Marginal I Basis Zone 1 |  |  |  | <.0001* |
| E | Lateral Stylus Zone 3 | Marginal II Stylus Zone 4 |  |  |  | <.0001* |
| E | Lateral Stylus Zone 2 | Marginal II Stylus Zone 3 |  |  |  | <.0001* |
| E | Central Stylus Zone 3 | Marginal I Cusp Zone 4 |  |  |  | <.0001* |
| E | Central Stylus Zone 2 | Lateral Cusp Zone 2 |  |  |  | <.0001* |
| E | Marginal II Basis Zone 4 | Marginal I Cusp Zone 2 |  |  |  | <.0001* |
| E | Marginal II Stylus Zone 2 | Marginal II Basis Zone 2 |  |  |  | <.0001* |
| E | Lateral Stylus Zone 2 | Marginal I Basis Zone 4 |  |  |  | <.0001* |
| E | Marginal I Basis Zone 4 | Marginal II Cusp Zone 2 |  |  |  | <.0001* |
| E | Marginal II Stylus Zone 2 | Marginal I Basis Zone 2 |  |  |  | <.0001* |
| E | Marginal I Stylus Zone 2 | Marginal II Basis Zone 2 |  |  |  | <.0001* |
| E | Marginal II Stylus Zone 4 | Marginal I Cusp Zone 3 |  |  |  | <.0001* |
| E | Marginal I Stylus Zone 2 | Marginal I Basis Zone 2 |  |  |  | <.0001* |
| E | Lateral Cusp Zone 3 | Marginal II Cusp Zone 4 |  |  |  | <.0001* |
| E | Lateral Stylus Zone 3 | Central Stylus Zone 2 |  |  |  | 0.0130* |
| E | Marginal II Basis Zone 4 | Marginal I Stylus Zone 3 |  |  |  | <.0001* |
| E | Marginal II Basis Zone 3 | Marginal II Basis Zone 2 |  |  |  | <.0001* |
| E | Lateral Cusp Zone 1 | Marginal I Cusp Zone 1 |  |  |  | <.0001* |
| E | Lateral Cusp Zone 1 | Marginal II Cusp Zone 1 |  |  |  | <.0001* |
| E | Marginal II Stylus Zone 2 | Lateral Cusp Zone 1 |  |  |  | <.0001* |
| E | Marginal II Stylus Zone 3 | Marginal II Cusp Zone 2 |  |  |  | <.0001* |
| E | Lateral Cusp Zone 2 | Marginal II Cusp Zone 3 |  |  |  | <.0001* |
| E | Marginal II Basis Zone 3 | Marginal I Basis Zone 2 |  |  |  | <.0001* |
| E | Marginal II Stylus Zone 4 | Lateral Cusp Zone 2 |  |  |  | <.0001* |
| E | Lateral Cusp Zone 1 | Lateral Stylus Zone 1 |  |  |  | <.0001* |
| E | Lateral Cusp Zone 1 | Marginal I Stylus Zone 1 |  |  |  | <.0001* |
| E | Lateral Cusp Zone 1 | Marginal II Stylus Zone 1 |  |  |  | <.0001* |
| E | Lateral Cusp Zone 1 | Marginal II Basis Zone 1 |  |  |  | <.0001* |
| E | Lateral Cusp Zone 1 | Marginal I Basis Zone 1 |  |  |  | <.0001* |
| E | Central Stylus Zone 1 | Marginal II Basis Zone 2 |  |  |  | 0.2060 |
| E | Marginal I Stylus Zone 2 | Lateral Cusp Zone 1 |  |  |  | <.0001* |
| E | Marginal I Stylus Zone 4 | Marginal I Cusp Zone 3 |  |  |  | <.0001* |
| E | Marginal I Basis Zone 3 | Marginal II Basis Zone 2 |  |  |  | 0.0007* |
| E | Marginal II Basis Zone 4 | Marginal II Stylus Zone 3 |  |  |  | <.0001* |
| E | Marginal I Basis Zone 2 | Marginal I Cusp Zone 1 |  |  |  | <.0001* |
| E | Marginal I Cusp Zone 3 | Marginal II Cusp Zone 3 |  |  |  | <.0001* |
| E | Marginal I Basis Zone 2 | Marginal II Cusp Zone 1 |  |  |  | 0.0003* |
| E | Central Stylus Zone 1 | Marginal I Basis Zone 2 |  |  |  | 0.2953 |
| E | Marginal I Stylus Zone 3 | Marginal II Cusp Zone 2 |  |  |  | 0.0002* |
| E | Marginal I Basis Zone 3 | Marginal I Basis Zone 2 |  |  |  | 0.0007* |
| E | Marginal II Basis Zone 3 | Lateral Cusp Zone 1 |  |  |  | 0.0017* |
| E | Marginal II Basis Zone 2 | Marginal I Cusp Zone 1 |  |  |  | 0.0028* |
| E | Marginal I Basis Zone 2 | Lateral Stylus Zone 1 |  |  |  | 0.0004* |
| E | Marginal II Basis Zone 2 | Marginal II Cusp Zone 1 |  |  |  | 0.0099* |
| E | Marginal I Basis Zone 2 | Marginal I Stylus Zone 1 |  |  |  | 0.0008* |
| E | Marginal I Basis Zone 2 | Marginal II Stylus Zone 1 |  |  |  | 0.0040* |
| E | Marginal I Basis Zone 2 | Marginal II Basis Zone 1 |  |  |  | 0.0142* |
| E | Marginal I Basis Zone 2 | Marginal I Basis Zone 1 |  |  |  | 0.0056* |
| E | Marginal I Stylus Zone 4 | Lateral Cusp Zone 2 |  |  |  | <.0001* |
| E | Marginal II Basis Zone 2 | Lateral Stylus Zone 1 |  |  |  | 0.0161* |
| E | Marginal II Basis Zone 2 | Marginal I Stylus Zone 1 |  |  |  | 0.0237* |
| E | Marginal II Basis Zone 2 | Marginal II Stylus Zone 1 |  |  |  | 0.0583 |
| E | Central Stylus Zone 2 | Marginal I Stylus Zone 4 |  |  |  | 0.5148 |
| E | Marginal II Basis Zone 2 | Marginal II Basis Zone 1 |  |  |  | 0.1271 |
| E | Marginal II Basis Zone 2 | Marginal I Basis Zone 1 |  |  |  | 0.0821 |
| E | Marginal II Basis Zone 4 | Marginal I Basis Zone 4 |  |  |  | <.0001* |
| E | Central Stylus Zone 1 | Lateral Cusp Zone 1 |  |  |  | 0.8141 |
| E | Marginal I Basis Zone 3 | Lateral Cusp Zone 1 |  |  |  | 0.0282* |
| E | Marginal I Basis Zone 4 | Marginal I Cusp Zone 2 |  |  |  | 0.0003* |
| E | Lateral Stylus Zone 2 | Marginal II Basis Zone 4 |  |  |  | 0.0037* |
| E | Marginal I Cusp Zone 2 | Marginal II Cusp Zone 2 |  |  |  | 0.0936 |
| E | Marginal I Cusp Zone 4 | Lateral Cusp Zone 3 |  |  |  | 0.0039* |
| E | Central Stylus Zone 2 | Marginal II Stylus Zone 4 |  |  |  | 0.9975 |
| E | Marginal II Stylus Zone 3 | Marginal I Cusp Zone 2 |  |  |  | 0.8593 |
| E | Marginal I Basis Zone 4 | Marginal I Stylus Zone 3 |  |  |  | 0.5282 |
| E | Marginal II Stylus Zone 2 | Marginal I Basis Zone 3 |  |  |  | 0.9774 |
| E | Marginal II Stylus Zone 2 | Central Stylus Zone 1 |  |  |  | 1.0000 |
| E | Marginal I Stylus Zone 2 | Marginal I Basis Zone 3 |  |  |  | 0.9999 |
| E | Lateral Cusp Zone 1 | Marginal II Basis Zone 2 |  |  |  | 1.0000 |
| E | Marginal I Stylus Zone 3 | Marginal I Cusp Zone 2 |  |  |  | 0.9999 |
| E | Marginal II Stylus Zone 4 | Marginal I Stylus Zone 4 |  |  |  | 0.8726 |
| E | Marginal I Stylus Zone 2 | Central Stylus Zone 1 |  |  |  | 1.0000 |
| E | Marginal I Basis Zone 4 | Marginal II Stylus Zone 3 |  |  |  | 1.0000 |
| E | Marginal II Stylus Zone 2 | Marginal II Basis Zone 3 |  |  |  | 1.0000 |
| E | Lateral Cusp Zone 1 | Marginal I Basis Zone 2 |  |  |  | 1.0000 |
| E | Marginal II Stylus Zone 3 | Marginal I Stylus Zone 3 |  |  |  | 1.0000 |
| E | Marginal II Basis Zone 3 | Marginal I Basis Zone 3 |  |  |  | 1.0000 |
| E | Lateral Cusp Zone 2 | Marginal I Cusp Zone 3 |  |  |  | 1.0000 |
| E | Marginal II Basis Zone 3 | Central Stylus Zone 1 |  |  |  | 1.0000 |
| E | Marginal II Stylus Zone 2 | Marginal I Stylus Zone 2 |  |  |  | 1.0000 |
| E | Marginal I Basis Zone 1 | Marginal I Cusp Zone 1 |  |  |  | 1.0000 |
| E | Marginal I Basis Zone 1 | Marginal II Cusp Zone 1 |  |  |  | 1.0000 |
| E | Marginal II Basis Zone 1 | Marginal I Cusp Zone 1 |  |  |  | 1.0000 |
| E | Marginal I Stylus Zone 2 | Marginal II Basis Zone 3 |  |  |  | 1.0000 |
| E | Marginal II Basis Zone 1 | Marginal II Cusp Zone 1 |  |  |  | 1.0000 |
| E | Marginal II Stylus Zone 1 | Marginal I Cusp Zone 1 |  |  |  | 1.0000 |
| E | Marginal I Stylus Zone 1 | Marginal I Cusp Zone 1 |  |  |  | 1.0000 |
| E | Lateral Stylus Zone 1 | Marginal I Cusp Zone 1 |  |  |  | 1.0000 |
| E | Marginal II Stylus Zone 1 | Marginal II Cusp Zone 1 |  |  |  | 1.0000 |
| E | Marginal I Stylus Zone 1 | Marginal II Cusp Zone 1 |  |  |  | 1.0000 |
| E | Marginal I Basis Zone 2 | Marginal II Basis Zone 2 |  |  |  | 1.0000 |
| E | Lateral Stylus Zone 1 | Marginal II Cusp Zone 1 |  |  |  | 1.0000 |
| E | Marginal I Basis Zone 1 | Lateral Stylus Zone 1 |  |  |  | 1.0000 |
| E | Marginal I Basis Zone 1 | Marginal I Stylus Zone 1 |  |  |  | 1.0000 |
| E | Marginal I Basis Zone 1 | Marginal II Stylus Zone 1 |  |  |  | 1.0000 |
| E | Marginal II Basis Zone 1 | Lateral Stylus Zone 1 |  |  |  | 1.0000 |
| E | Central Stylus Zone 1 | Marginal I Basis Zone 3 |  |  |  | 1.0000 |
| E | Marginal II Basis Zone 1 | Marginal I Stylus Zone 1 |  |  |  | 1.0000 |
| E | Marginal II Basis Zone 1 | Marginal II Stylus Zone 1 |  |  |  | 1.0000 |
| E | Marginal I Basis Zone 1 | Marginal II Basis Zone 1 |  |  |  | 1.0000 |
| E | Marginal II Cusp Zone 1 | Marginal I Cusp Zone 1 |  |  |  | 1.0000 |
| E | Marginal II Stylus Zone 1 | Lateral Stylus Zone 1 |  |  |  | 1.0000 |
| E | Marginal I Stylus Zone 1 | Lateral Stylus Zone 1 |  |  |  | 1.0000 |
| E | Marginal II Stylus Zone 1 | Marginal I Stylus Zone 1 |  |  |  | 1.0000 |

**Supplementary Table 2.** Proportions of the individual elements: pairwise comparison between the zones and tooth types performed according to the Wilcoxon method (orange p-values = highly significant, red = significant, black = not significant).

| **Parameter** | **Structure 1** | **Structure 2** | **1-Way Test, ChiSquare approximation** | | | **Wilcoxon method** |
| --- | --- | --- | --- | --- | --- | --- |
|  |  |  | **ChiSquare** | **df** | **p-value** | **p-value** |
| All elements | Zone 3 | Zone 1 | 32.6677 | 3 | <.0001* | <.0001* |
| All elements | Zone 2 | Zone 1 |  |  |  | <.0001* |
| All elements | Zone 4 | Zone 1 |  |  |  | 0.0004* |
| All elements | Zone 3 | Zone 2 |  |  |  | 0.0335* |
| All elements | Zone 4 | Zone 2 |  |  |  | 0.6023 |
| All elements | Zone 4 | Zone 3 |  |  |  | 0.2853 |
| Ca | Zone 4 | Zone 1 | 10.5484 | 3 | 0.0144* | 0.0015* |
| Ca | Zone 4 | Zone 2 |  |  |  | 0.0223* |
| Ca | Zone 3 | Zone 1 |  |  |  | 0.0972 |
| Ca | Zone 4 | Zone 3 |  |  |  | 0.2185 |
| Ca | Zone 2 | Zone 1 |  |  |  | 0.2589 |
| Ca | Zone 3 | Zone 2 |  |  |  | 0.3909 |
| Cl | Zone 4 | Zone 1 | 19.1450 | 3 | 0.0003* | 0.0001* |
| Cl | Zone 3 | Zone 1 |  |  |  | 0.0005* |
| Cl | Zone 2 | Zone 1 |  |  |  | 0.0114* |
| Cl | Zone 4 | Zone 2 |  |  |  | 0.0864 |
| Cl | Zone 3 | Zone 2 |  |  |  | 0.2131 |
| Cl | Zone 4 | Zone 3 |  |  |  | 0.8735 |
| Mg | Zone 3 | Zone 1 | 30.1555 | 3 | <.0001* | <.0001* |
| Mg | Zone 4 | Zone 1 |  |  |  | <.0001* |
| Mg | Zone 2 | Zone 1 |  |  |  | 0.0009* |
| Mg | Zone 4 | Zone 2 |  |  |  | 0.0320* |
| Mg | Zone 3 | Zone 2 |  |  |  | 0.0684 |
| Mg | Zone 4 | Zone 3 |  |  |  | 0.9148 |
| Na | Zone 3 | Zone 1 | 69.6560 | 3 | <.0001* | <.0001* |
| Na | Zone 2 | Zone 1 |  |  |  | <.0001* |
| Na | Zone 4 | Zone 1 |  |  |  | <.0001* |
| Na | Zone 3 | Zone 2 |  |  |  | <.0001* |
| Na | Zone 4 | Zone 2 |  |  |  | 0.0093* |
| Na | Zone 4 | Zone 3 |  |  |  | 0.4644 |
| P | Zone 4 | Zone 1 | 9.8423 | 3 | 0.0200* | 0.0066* |
| P | Zone 3 | Zone 1 |  |  |  | 0.0219* |
| P | Zone 4 | Zone 2 |  |  |  | 0.0621 |
| P | Zone 3 | Zone 2 |  |  |  | 0.1185 |
| P | Zone 2 | Zone 1 |  |  |  | 0.2776 |
| P | Zone 4 | Zone 3 |  |  |  | 0.9437 |
| S | Zone 4 | Zone 1 | 40.6278 | 3 | <.0001* | <.0001* |
| S | Zone 4 | Zone 1 |  |  |  | <.0001* |
| S | Zone 2 | Zone 1 |  |  |  | <.0001* |
| S | Zone 3 | Zone 2 |  |  |  | 0.0151* |
| S | Zone 4 | Zone 2 |  |  |  | 0.0453* |
| S | Zone 4 | Zone 3 |  |  |  | 0.6446 |
| Si | Zone 3 | Zone 1 | 22.6226 | 3 | <.0001* | <.0001* |
| Si | Zone 4 | Zone 1 |  |  |  | 0.0002* |
| Si | Zone 2 | Zone 1 |  |  |  | 0.0098* |
| Si | Zone 3 | Zone 2 |  |  |  | 0.0443* |
| Si | Zone 4 | Zone 2 |  |  |  | 0.0559 |
| Si | Zone 4 | Zone 3 |  |  |  | 0.8032 |
| All elements | Lateral Zone 3 | Central Zone 1 | 146.7164 | 15 | <.0001* | <.0001* |
| All elements | Lateral Zone 4 | Central Zone 1 |  |  |  | <.0001* |
| All elements | Lateral Zone 2 | Central Zone 1 |  |  |  | <.0001* |
| All elements | Central Zone 3 | Central Zone 1 |  |  |  | <.0001* |
| All elements | Central Zone 4 | Central Zone 1 |  |  |  | 0.0008* |
| All elements | Lateral Zone 3 | Central Zone 2 |  |  |  | 0.0009* |
| All elements | Lateral Zone 3 | Lateral Zone 1 |  |  |  | 0.0006* |
| All elements | Lateral Zone 4 | Central Zone 2 |  |  |  | 0.0017* |
| All elements | Central Zone 2 | Central Zone 1 |  |  |  | 0.0034* |
| All elements | Lateral Zone 4 | Lateral Zone 1 |  |  |  | 0.0010* |
| All elements | Lateral Zone 3 | Central Zone 4 |  |  |  | 0.0032* |
| All elements | Marginal I Zone 3 | Marginal I Zone 1 |  |  |  | 0.0008* |
| All elements | Marginal I Zone 2 | Marginal I Zone 1 |  |  |  | 0.0015* |
| All elements | Lateral Zone 2 | Lateral Zone 1 |  |  |  | 0.0035* |
| All elements | Marginal II Zone 3 | Marginal II Zone 1 |  |  |  | 0.0041* |
| All elements | Lateral Zone 4 | Central Zone 4 |  |  |  | 0.0083* |
| All elements | Lateral Zone 1 | Central Zone 1 |  |  |  | 0.0103* |
| All elements | Marginal II Zone 2 | Marginal II Zone 1 |  |  |  | 0.0063* |
| All elements | Lateral Zone 2 | Central Zone 2 |  |  |  | 0.0115* |
| All elements | Lateral Zone 3 | Central Zone 3 |  |  |  | 0.0128* |
| All elements | Marginal II Zone 3 | Marginal I Zone 1 |  |  |  | 0.0081* |
| All elements | Marginal II Zone 2 | Marginal I Zone 1 |  |  |  | 0.0193* |
| All elements | Lateral Zone 4 | Central Zone 3 |  |  |  | 0.0421* |
| All elements | Lateral Zone 2 | Central Zone 4 |  |  |  | 0.0712 |
| All elements | Marginal II Zone 3 | Marginal II Zone 2 |  |  |  | 0.0628 |
| All elements | Marginal I Zone 3 | Marginal I Zone 2 |  |  |  | 0.0567 |
| All elements | Central Zone 3 | Central Zone 2 |  |  |  | 0.1249 |
| All elements | Marginal II Zone 3 | Marginal I Zone 2 |  |  |  | 0.1572 |
| All elements | Marginal I Zone 4 | Marginal I Zone 1 |  |  |  | 0.1452 |
| All elements | Lateral Zone 2 | Central Zone 3 |  |  |  | 0.2625 |
| All elements | Lateral Zone 3 | Lateral Zone 2 |  |  |  | 0.2802 |
| All elements | Marginal II Zone 3 | Marginal I Zone 4 |  |  |  | 0.2986 |
| All elements | Marginal I Zone 3 | Central Zone 1 |  |  |  | 0.4588 |
| All elements | Lateral Zone 4 | Lateral Zone 2 |  |  |  | 0.4484 |
| All elements | Central Zone 4 | Central Zone 2 |  |  |  | 0.5166 |
| All elements | Marginal II Zone 3 | Central Zone 1 |  |  |  | 0.6349 |
| All elements | Marginal I Zone 4 | Marginal I Zone 2 |  |  |  | 0.6441 |
| All elements | Marginal II Zone 4 | Marginal II Zone 1 |  |  |  | 0.7896 |
| All elements | Marginal II Zone 1 | Marginal I Zone 1 |  |  |  | 0.8977 |
| All elements | Marginal II Zone 3 | Marginal I Zone 3 |  |  |  | 0.9795 |
| All elements | Marginal II Zone 2 | Marginal I Zone 2 |  |  |  | 0.9795 |
| All elements | Marginal II Zone 4 | Marginal I Zone 1 |  |  |  | 0.9783 |
| All elements | Marginal II Zone 2 | Marginal I Zone 4 |  |  |  | 0.9247 |
| All elements | Lateral Zone 1 | Central Zone 2 |  |  |  | 0.6349 |
| All elements | Marginal II Zone 4 | Marginal I Zone 4 |  |  |  | 0.5257 |
| All elements | Lateral Zone 4 | Lateral Zone 3 |  |  |  | 0.5351 |
| All elements | Marginal I Zone 4 | Central Zone 1 |  |  |  | 0.4875 |
| All elements | Marginal I Zone 2 | Central Zone 1 |  |  |  | 0.4091 |
| All elements | Marginal I Zone 4 | Marginal I Zone 3 |  |  |  | 0.2410 |
| All elements | Central Zone 4 | Central Zone 3 |  |  |  | 0.3038 |
| All elements | Lateral Zone 1 | Central Zone 4 |  |  |  | 0.2790 |
| All elements | Marginal II Zone 2 | Central Zone 1 |  |  |  | 0.2790 |
| All elements | Marginal II Zone 4 | Marginal I Zone 2 |  |  |  | 0.2012 |
| All elements | Marginal II Zone 1 | Marginal I Zone 4 |  |  |  | 0.1564 |
| All elements | Marginal II Zone 4 | Marginal II Zone 2 |  |  |  | 0.1148 |
| All elements | Marginal I Zone 3 | Lateral Zone 1 |  |  |  | 0.0760 |
| All elements | Marginal II Zone 3 | Lateral Zone 1 |  |  |  | 0.0409* |
| All elements | Lateral Zone 1 | Central Zone 3 |  |  |  | 0.0551 |
| All elements | Marginal I Zone 4 | Lateral Zone 1 |  |  |  | 0.0253* |
| All elements | Marginal II Zone 2 | Marginal I Zone 3 |  |  |  | 0.0288* |
| All elements | Marginal II Zone 4 | Marginal II Zone 3 |  |  |  | 0.0308* |
| All elements | Marginal II Zone 4 | Marginal I Zone 3 |  |  |  | 0.0240* |
| All elements | Marginal I Zone 3 | Central Zone 2 |  |  |  | 0.0401* |
| All elements | Marginal II Zone 4 | Central Zone 1 |  |  |  | 0.0263* |
| All elements | Marginal II Zone 3 | Central Zone 2 |  |  |  | 0.0176* |
| All elements | Marginal I Zone 2 | Lateral Zone 1 |  |  |  | 0.0051* |
| All elements | Marginal I Zone 4 | Central Zone 2 |  |  |  | 0.0069* |
| All elements | Marginal I Zone 4 | Central Zone 4 |  |  |  | 0.0069* |
| All elements | Marginal I Zone 3 | Central Zone 4 |  |  |  | 0.0056* |
| All elements | Marginal II Zone 1 | Marginal I Zone 2 |  |  |  | 0.0016* |
| All elements | Marginal II Zone 2 | Lateral Zone 1 |  |  |  | 0.0016* |
| All elements | Marginal II Zone 3 | Central Zone 4 |  |  |  | 0.0025* |
| All elements | Marginal II Zone 4 | Lateral Zone 1 |  |  |  | 0.0009* |
| All elements | Marginal I Zone 4 | Central Zone 3 |  |  |  | 0.0016* |
| All elements | Marginal I Zone 4 | Lateral Zone 2 |  |  |  | 0.0003* |
| All elements | Marginal II Zone 1 | Marginal I Zone 3 |  |  |  | 0.0004* |
| All elements | Marginal I Zone 4 | Lateral Zone 3 |  |  |  | 0.0002* |
| All elements | Marginal I Zone 4 | Lateral Zone 4 |  |  |  | 0.0002* |
| All elements | Marginal I Zone 3 | Central Zone 3 |  |  |  | 0.0008* |
| All elements | Marginal I Zone 1 | Central Zone 1 |  |  |  | 0.0007* |
| All elements | Marginal I Zone 2 | Central Zone 2 |  |  |  | 0.0006* |
| All elements | Marginal I Zone 2 | Central Zone 4 |  |  |  | 0.0005* |
| All elements | Marginal II Zone 2 | Central Zone 4 |  |  |  | 0.0004* |
| All elements | Marginal II Zone 4 | Central Zone 4 |  |  |  | 0.0003* |
| All elements | Marginal I Zone 3 | Lateral Zone 2 |  |  |  | <.0001* |
| All elements | Marginal II Zone 2 | Central Zone 2 |  |  |  | 0.0003* |
| All elements | Marginal I Zone 1 | Lateral Zone 1 |  |  |  | <.0001* |
| All elements | Marginal I Zone 3 | Lateral Zone 3 |  |  |  | <.0001* |
| All elements | Marginal I Zone 3 | Lateral Zone 4 |  |  |  | <.0001* |
| All elements | Marginal II Zone 3 | Central Zone 3 |  |  |  | 0.0002* |
| All elements | Marginal II Zone 1 | Central Zone 1 |  |  |  | 0.0002* |
| All elements | Marginal II Zone 4 | Central Zone 2 |  |  |  | 0.0001* |
| All elements | Marginal II Zone 4 | Lateral Zone 2 |  |  |  | <.0001* |
| All elements | Marginal I Zone 2 | Lateral Zone 2 |  |  |  | <.0001* |
| All elements | Marginal II Zone 3 | Lateral Zone 2 |  |  |  | <.0001* |
| All elements | Marginal I Zone 1 | Lateral Zone 2 |  |  |  | <.0001* |
| All elements | Marginal I Zone 1 | Lateral Zone 3 |  |  |  | <.0001* |
| All elements | Marginal I Zone 1 | Lateral Zone 4 |  |  |  | <.0001* |
| All elements | Marginal I Zone 2 | Lateral Zone 3 |  |  |  | <.0001* |
| All elements | Marginal I Zone 2 | Lateral Zone 4 |  |  |  | <.0001* |
| All elements | Marginal II Zone 4 | Lateral Zone 4 |  |  |  | <.0001* |
| All elements | Marginal II Zone 4 | Lateral Zone 3 |  |  |  | <.0001* |
| All elements | Marginal I Zone 2 | Central Zone 3 |  |  |  | <.0001* |
| All elements | Marginal II Zone 3 | Lateral Zone 4 |  |  |  | <.0001* |
| All elements | Marginal II Zone 4 | Central Zone 3 |  |  |  | <.0001* |
| All elements | Marginal II Zone 1 | Lateral Zone 1 |  |  |  | <.0001* |
| All elements | Marginal II Zone 2 | Lateral Zone 2 |  |  |  | <.0001* |
| All elements | Marginal II Zone 3 | Lateral Zone 3 |  |  |  | <.0001* |
| All elements | Marginal II Zone 2 | Lateral Zone 4 |  |  |  | <.0001* |
| All elements | Marginal II Zone 1 | Lateral Zone 2 |  |  |  | <.0001* |
| All elements | Marginal II Zone 1 | Lateral Zone 3 |  |  |  | <.0001* |
| All elements | Marginal II Zone 1 | Lateral Zone 4 |  |  |  | <.0001* |
| All elements | Marginal II Zone 2 | Lateral Zone 3 |  |  |  | <.0001* |
| All elements | Marginal II Zone 2 | Central Zone 3 |  |  |  | <.0001* |
| All elements | Marginal I Zone 1 | Central Zone 4 |  |  |  | <.0001* |
| All elements | Marginal I Zone 1 | Central Zone 2 |  |  |  | <.0001* |
| All elements | Marginal I Zone 1 | Central Zone 3 |  |  |  | <.0001* |
| All elements | Marginal II Zone 1 | Central Zone 4 |  |  |  | <.0001* |
| All elements | Marginal II Zone 1 | Central Zone 2 |  |  |  | <.0001* |
| All elements | Marginal II Zone 1 | Central Zone 3 |  |  |  | <.0001* |
| Ca | Lateral Zone 3 | Lateral Zone 1 | 99.5815 | 14 | <.0001* | 0.0079* |
| Ca | Central Zone 3 | Central Zone 1 |  |  |  | 0.0354* |
| Ca | Central Zone 4 | Central Zone 1 |  |  |  | 0.0555 |
| Ca | Lateral Zone 4 | Lateral Zone 1 |  |  |  | 0.0326* |
| Ca | Lateral Zone 3 | Central Zone 1 |  |  |  | 0.0621 |
| Ca | Central Zone 2 | Central Zone 1 |  |  |  | 0.0967 |
| Ca | Lateral Zone 3 | Lateral Zone 2 |  |  |  | 0.0520 |
| Ca | Lateral Zone 2 | Lateral Zone 1 |  |  |  | 0.0695 |
| Ca | Marginal II Zone 3 | Marginal I Zone 1 |  |  |  | 0.0262* |
| Ca | Marginal II Zone 3 | Marginal I Zone 2 |  |  |  | 0.0233* |
| Ca | Marginal II Zone 2 | Marginal I Zone 1 |  |  |  | 0.0640 |
| Ca | Marginal II Zone 4 | Marginal II Zone 1 |  |  |  | 0.0504 |
| Ca | Marginal II Zone 4 | Marginal I Zone 2 |  |  |  | 0.0200* |
| Ca | Lateral Zone 4 | Central Zone 1 |  |  |  | 0.2031 |
| Ca | Marginal II Zone 2 | Marginal I Zone 2 |  |  |  | 0.0703 |
| Ca | Marginal II Zone 4 | Marginal II Zone 2 |  |  |  | 0.0756 |
| Ca | Marginal II Zone 3 | Marginal II Zone 1 |  |  |  | 0.0918 |
| Ca | Marginal II Zone 3 | Marginal I Zone 3 |  |  |  | 0.0901 |
| Ca | Lateral Zone 3 | Central Zone 2 |  |  |  | 0.2336 |
| Ca | Central Zone 3 | Central Zone 2 |  |  |  | 0.3038 |
| Ca | Marginal II Zone 4 | Marginal I Zone 3 |  |  |  | 0.0472* |
| Ca | Lateral Zone 3 | Central Zone 4 |  |  |  | 0.2706 |
| Ca | Marginal II Zone 1 | Marginal I Zone 1 |  |  |  | 0.1223 |
| Ca | Lateral Zone 2 | Central Zone 1 |  |  |  | 0.3141 |
| Ca | Marginal II Zone 2 | Marginal II Zone 1 |  |  |  | 0.1771 |
| Ca | Lateral Zone 3 | Central Zone 3 |  |  |  | 0.3118 |
| Ca | Marginal II Zone 4 | Marginal I Zone 1 |  |  |  | 0.0518 |
| Ca | Central Zone 4 | Central Zone 2 |  |  |  | 0.4017 |
| Ca | Lateral Zone 4 | Lateral Zone 2 |  |  |  | 0.3703 |
| Ca | Marginal I Zone 3 | Marginal I Zone 1 |  |  |  | 0.1314 |
| Ca | Marginal II Zone 2 | Marginal I Zone 3 |  |  |  | 0.2845 |
| Ca | Marginal II Zone 3 | Marginal II Zone 2 |  |  |  | 0.3767 |
| Ca | Lateral Zone 4 | Central Zone 2 |  |  |  | 0.5817 |
| Ca | Marginal I Zone 3 | Marginal I Zone 2 |  |  |  | 0.4005 |
| Ca | Lateral Zone 1 | Central Zone 1 |  |  |  | 0.6349 |
| Ca | Lateral Zone 4 | Central Zone 4 |  |  |  | 0.6348 |
| Ca | Marginal II Zone 4 | Marginal II Zone 3 |  |  |  | 0.5360 |
| Ca | Lateral Zone 2 | Central Zone 2 |  |  |  | 0.6900 |
| Ca | Marginal I Zone 2 | Marginal I Zone 1 |  |  |  | 0.4534 |
| Ca | Lateral Zone 4 | Central Zone 3 |  |  |  | 0.7182 |
| Ca | Marginal II Zone 1 | Marginal I Zone 2 |  |  |  | 0.6064 |
| Ca | Lateral Zone 2 | Central Zone 4 |  |  |  | 0.9848 |
| Ca | Marginal II Zone 1 | Marginal I Zone 3 |  |  |  | 1.0000 |
| Ca | Lateral Zone 2 | Central Zone 3 |  |  |  | 0.8942 |
| Ca | Central Zone 4 | Central Zone 3 |  |  |  | 0.6350 |
| Ca | Lateral Zone 1 | Central Zone 2 |  |  |  | 0.3520 |
| Ca | Lateral Zone 4 | Lateral Zone 3 |  |  |  | 0.2180 |
| Ca | Lateral Zone 1 | Central Zone 4 |  |  |  | 0.2313 |
| Ca | Lateral Zone 1 | Central Zone 3 |  |  |  | 0.1655 |
| Ca | Marginal II Zone 4 | Central Zone 1 |  |  |  | 0.0671 |
| Ca | Marginal I Zone 1 | Lateral Zone 3 |  |  |  | 0.0127* |
| Ca | Marginal II Zone 4 | Lateral Zone 1 |  |  |  | 0.0169* |
| Ca | Marginal II Zone 4 | Lateral Zone 3 |  |  |  | 0.0050* |
| Ca | Marginal I Zone 2 | Lateral Zone 3 |  |  |  | 0.0022* |
| Ca | Marginal I Zone 3 | Lateral Zone 3 |  |  |  | 0.0022* |
| Ca | Marginal I Zone 1 | Lateral Zone 1 |  |  |  | 0.0098* |
| Ca | Marginal I Zone 1 | Lateral Zone 2 |  |  |  | 0.0098* |
| Ca | Marginal I Zone 1 | Lateral Zone 4 |  |  |  | 0.0098* |
| Ca | Marginal II Zone 4 | Lateral Zone 2 |  |  |  | 0.0035* |
| Ca | Marginal II Zone 4 | Lateral Zone 4 |  |  |  | 0.0035* |
| Ca | Marginal I Zone 2 | Lateral Zone 1 |  |  |  | 0.0014* |
| Ca | Marginal I Zone 2 | Lateral Zone 2 |  |  |  | 0.0014* |
| Ca | Marginal I Zone 2 | Lateral Zone 4 |  |  |  | 0.0014* |
| Ca | Marginal I Zone 3 | Lateral Zone 1 |  |  |  | 0.0014* |
| Ca | Marginal I Zone 3 | Lateral Zone 2 |  |  |  | 0.0014* |
| Ca | Marginal I Zone 3 | Lateral Zone 4 |  |  |  | 0.0014* |
| Ca | Marginal II Zone 1 | Lateral Zone 3 |  |  |  | 0.0003* |
| Ca | Marginal II Zone 2 | Lateral Zone 3 |  |  |  | 0.0002* |
| Ca | Marginal II Zone 3 | Lateral Zone 3 |  |  |  | 0.0002* |
| Ca | Marginal II Zone 3 | Lateral Zone 1 |  |  |  | 0.0005* |
| Ca | Marginal II Zone 4 | Central Zone 2 |  |  |  | 0.0043* |
| Ca | Marginal II Zone 3 | Central Zone 1 |  |  |  | 0.0016* |
| Ca | Marginal I Zone 1 | Central Zone 1 |  |  |  | 0.0077* |
| Ca | Marginal I Zone 1 | Central Zone 2 |  |  |  | 0.0077* |
| Ca | Marginal I Zone 1 | Central Zone 3 |  |  |  | 0.0077* |
| Ca | Marginal I Zone 1 | Central Zone 4 |  |  |  | 0.0077* |
| Ca | Marginal II Zone 4 | Central Zone 3 |  |  |  | 0.0025* |
| Ca | Marginal II Zone 4 | Central Zone 4 |  |  |  | 0.0025* |
| Ca | Marginal II Zone 2 | Lateral Zone 1 |  |  |  | 0.0002* |
| Ca | Marginal II Zone 1 | Lateral Zone 1 |  |  |  | 0.0002* |
| Ca | Marginal II Zone 1 | Lateral Zone 2 |  |  |  | 0.0002* |
| Ca | Marginal II Zone 1 | Lateral Zone 4 |  |  |  | 0.0002* |
| Ca | Marginal II Zone 2 | Central Zone 1 |  |  |  | 0.0006* |
| Ca | Marginal I Zone 2 | Central Zone 1 |  |  |  | 0.0009* |
| Ca | Marginal I Zone 2 | Central Zone 2 |  |  |  | 0.0009* |
| Ca | Marginal I Zone 2 | Central Zone 3 |  |  |  | 0.0009* |
| Ca | Marginal I Zone 2 | Central Zone 4 |  |  |  | 0.0009* |
| Ca | Marginal I Zone 3 | Central Zone 1 |  |  |  | 0.0009* |
| Ca | Marginal I Zone 3 | Central Zone 2 |  |  |  | 0.0009* |
| Ca | Marginal I Zone 3 | Central Zone 3 |  |  |  | 0.0009* |
| Ca | Marginal I Zone 3 | Central Zone 4 |  |  |  | 0.0009* |
| Ca | Marginal II Zone 2 | Lateral Zone 2 |  |  |  | <.0001* |
| Ca | Marginal II Zone 2 | Lateral Zone 4 |  |  |  | <.0001* |
| Ca | Marginal II Zone 3 | Lateral Zone 2 |  |  |  | <.0001* |
| Ca | Marginal II Zone 3 | Lateral Zone 4 |  |  |  | <.0001* |
| Ca | Marginal II Zone 1 | Central Zone 1 |  |  |  | <.0001* |
| Ca | Marginal II Zone 1 | Central Zone 2 |  |  |  | <.0001* |
| Ca | Marginal II Zone 1 | Central Zone 3 |  |  |  | <.0001* |
| Ca | Marginal II Zone 1 | Central Zone 4 |  |  |  | <.0001* |
| Ca | Marginal II Zone 3 | Central Zone 2 |  |  |  | <.0001* |
| Ca | Marginal II Zone 2 | Central Zone 2 |  |  |  | <.0001* |
| Ca | Marginal II Zone 2 | Central Zone 3 |  |  |  | <.0001* |
| Ca | Marginal II Zone 2 | Central Zone 4 |  |  |  | <.0001* |
| Ca | Marginal II Zone 3 | Central Zone 3 |  |  |  | <.0001* |
| Ca | Marginal II Zone 3 | Central Zone 4 |  |  |  | <.0001* |
| Cl | Lateral Zone 3 | Central Zone 1 | 82.7762 | 13 | <.0001* | <.0001* |
| Cl | Lateral Zone 2 | Central Zone 1 |  |  |  | <.0001* |
| Cl | Central Zone 3 | Central Zone 1 |  |  |  | <.0001* |
| Cl | Lateral Zone 4 | Central Zone 1 |  |  |  | 0.0002* |
| Cl | Central Zone 2 | Central Zone 1 |  |  |  | 0.0005* |
| Cl | Lateral Zone 3 | Central Zone 4 |  |  |  | 0.0004* |
| Cl | Lateral Zone 2 | Central Zone 4 |  |  |  | 0.0006* |
| Cl | Central Zone 4 | Central Zone 1 |  |  |  | 0.0015* |
| Cl | Lateral Zone 3 | Central Zone 2 |  |  |  | 0.0003* |
| Cl | Lateral Zone 3 | Central Zone 3 |  |  |  | 0.0009* |
| Cl | Lateral Zone 2 | Central Zone 2 |  |  |  | 0.0007* |
| Cl | Lateral Zone 2 | Central Zone 3 |  |  |  | 0.0012* |
| Cl | Lateral Zone 2 | Lateral Zone 1 |  |  |  | 0.0011* |
| Cl | Lateral Zone 3 | Lateral Zone 1 |  |  |  | 0.0011* |
| Cl | Lateral Zone 1 | Central Zone 1 |  |  |  | 0.0103* |
| Cl | Lateral Zone 4 | Central Zone 4 |  |  |  | 0.0103* |
| Cl | Lateral Zone 4 | Central Zone 2 |  |  |  | 0.0106* |
| Cl | Lateral Zone 4 | Lateral Zone 1 |  |  |  | 0.0139* |
| Cl | Lateral Zone 4 | Central Zone 3 |  |  |  | 0.0261* |
| Cl | Lateral Zone 3 | Lateral Zone 2 |  |  |  | 0.0141* |
| Cl | Central Zone 3 | Central Zone 2 |  |  |  | 0.1280 |
| Cl | Lateral Zone 1 | Central Zone 2 |  |  |  | 0.2299 |
| Cl | Lateral Zone 4 | Lateral Zone 2 |  |  |  | 0.2914 |
| Cl | Marginal I Zone 3 | Marginal I Zone 1 |  |  |  | 0.2703 |
| Cl | Central Zone 4 | Central Zone 2 |  |  |  | 0.5750 |
| Cl | Marginal I Zone 3 | Marginal I Zone 2 |  |  |  | 0.4034 |
| Cl | Marginal I Zone 2 | Marginal I Zone 1 |  |  |  | 0.3913 |
| Cl | Lateral Zone 1 | Central Zone 4 |  |  |  | 0.7180 |
| Cl | Marginal II Zone 3 | Marginal II Zone 2 |  |  |  | 0.5296 |
| Cl | Marginal II Zone 3 | Marginal II Zone 1 |  |  |  | 0.5486 |
| Cl | Marginal I Zone 3 | Central Zone 1 |  |  |  | 0.7941 |
| Cl | Marginal II Zone 2 | Marginal II Zone 1 |  |  |  | 0.6528 |
| Cl | Marginal II Zone 1 | Marginal I Zone 2 |  |  |  | 0.5510 |
| Cl | Marginal II Zone 3 | Marginal I Zone 2 |  |  |  | 0.5296 |
| Cl | Marginal II Zone 1 | Marginal I Zone 1 |  |  |  | 0.3768 |
| Cl | Lateral Zone 1 | Central Zone 3 |  |  |  | 0.6325 |
| Cl | Marginal II Zone 2 | Marginal I Zone 2 |  |  |  | 0.4034 |
| Cl | Marginal II Zone 3 | Marginal I Zone 3 |  |  |  | 0.3443 |
| Cl | Marginal II Zone 2 | Marginal I Zone 3 |  |  |  | 0.2963 |
| Cl | Marginal II Zone 3 | Marginal I Zone 1 |  |  |  | 0.2683 |
| Cl | Marginal II Zone 2 | Marginal I Zone 1 |  |  |  | 0.1779 |
| Cl | Marginal II Zone 1 | Marginal I Zone 3 |  |  |  | 0.1360 |
| Cl | Marginal I Zone 2 | Central Zone 1 |  |  |  | 0.4337 |
| Cl | Marginal II Zone 3 | Central Zone 1 |  |  |  | 0.2477 |
| Cl | Central Zone 4 | Central Zone 3 |  |  |  | 0.2139 |
| Cl | Marginal I Zone 1 | Central Zone 1 |  |  |  | 0.2170 |
| Cl | Lateral Zone 4 | Lateral Zone 3 |  |  |  | 0.1050 |
| Cl | Marginal I Zone 3 | Lateral Zone 1 |  |  |  | 0.0865 |
| Cl | Marginal I Zone 1 | Lateral Zone 1 |  |  |  | 0.0628 |
| Cl | Marginal II Zone 1 | Lateral Zone 1 |  |  |  | 0.0674 |
| Cl | Marginal I Zone 3 | Central Zone 4 |  |  |  | 0.0793 |
| Cl | Marginal I Zone 2 | Lateral Zone 1 |  |  |  | 0.0371* |
| Cl | Marginal I Zone 3 | Central Zone 2 |  |  |  | 0.0447* |
| Cl | Marginal I Zone 1 | Lateral Zone 4 |  |  |  | 0.0381* |
| Cl | Marginal II Zone 2 | Central Zone 1 |  |  |  | 0.0573 |
| Cl | Marginal I Zone 3 | Lateral Zone 4 |  |  |  | 0.0231* |
| Cl | Marginal II Zone 2 | Lateral Zone 1 |  |  |  | 0.0232* |
| Cl | Marginal II Zone 3 | Lateral Zone 1 |  |  |  | 0.0231* |
| Cl | Marginal I Zone 3 | Central Zone 3 |  |  |  | 0.0286* |
| Cl | Marginal I Zone 2 | Lateral Zone 4 |  |  |  | 0.0140* |
| Cl | Marginal I Zone 2 | Central Zone 4 |  |  |  | 0.0333* |
| Cl | Marginal II Zone 1 | Lateral Zone 2 |  |  |  | 0.0115* |
| Cl | Marginal II Zone 1 | Lateral Zone 3 |  |  |  | 0.0115* |
| Cl | Marginal I Zone 1 | Central Zone 4 |  |  |  | 0.0366* |
| Cl | Marginal II Zone 1 | Lateral Zone 4 |  |  |  | 0.0195* |
| Cl | Marginal II Zone 1 | Central Zone 1 |  |  |  | 0.0499* |
| Cl | Marginal I Zone 2 | Central Zone 2 |  |  |  | 0.0114* |
| Cl | Marginal II Zone 3 | Lateral Zone 4 |  |  |  | 0.0082* |
| Cl | Marginal I Zone 3 | Lateral Zone 2 |  |  |  | 0.0037* |
| Cl | Marginal I Zone 3 | Lateral Zone 3 |  |  |  | 0.0037* |
| Cl | Marginal II Zone 3 | Lateral Zone 2 |  |  |  | 0.0037* |
| Cl | Marginal II Zone 3 | Lateral Zone 3 |  |  |  | 0.0037* |
| Cl | Marginal I Zone 1 | Lateral Zone 2 |  |  |  | 0.0043* |
| Cl | Marginal I Zone 1 | Lateral Zone 3 |  |  |  | 0.0043* |
| Cl | Marginal II Zone 3 | Central Zone 2 |  |  |  | 0.0088* |
| Cl | Marginal I Zone 2 | Lateral Zone 2 |  |  |  | 0.0019* |
| Cl | Marginal I Zone 2 | Lateral Zone 3 |  |  |  | 0.0019* |
| Cl | Marginal II Zone 2 | Lateral Zone 2 |  |  |  | 0.0019* |
| Cl | Marginal II Zone 2 | Lateral Zone 3 |  |  |  | 0.0019* |
| Cl | Marginal II Zone 1 | Central Zone 2 |  |  |  | 0.0129* |
| Cl | Marginal II Zone 1 | Central Zone 3 |  |  |  | 0.0162* |
| Cl | Marginal I Zone 1 | Central Zone 3 |  |  |  | 0.0093* |
| Cl | Marginal II Zone 3 | Central Zone 4 |  |  |  | 0.0109* |
| Cl | Marginal I Zone 1 | Central Zone 2 |  |  |  | 0.0060* |
| Cl | Marginal II Zone 1 | Central Zone 4 |  |  |  | 0.0234* |
| Cl | Marginal I Zone 2 | Central Zone 3 |  |  |  | 0.0057* |
| Cl | Marginal II Zone 2 | Lateral Zone 4 |  |  |  | 0.0026* |
| Cl | Marginal II Zone 2 | Central Zone 2 |  |  |  | 0.0030* |
| Cl | Marginal II Zone 3 | Central Zone 3 |  |  |  | 0.0034* |
| Cl | Marginal II Zone 2 | Central Zone 3 |  |  |  | 0.0020* |
| Cl | Marginal II Zone 2 | Central Zone 4 |  |  |  | 0.0032* |
| Mg | Central Zone 3 | Central Zone 1 | 122.7658 | 15 | <.0001* | <.0001* |
| Mg | Lateral Zone 3 | Central Zone 1 |  |  |  | <.0001* |
| Mg | Lateral Zone 4 | Central Zone 1 |  |  |  | <.0001* |
| Mg | Central Zone 4 | Central Zone 1 |  |  |  | <.0001* |
| Mg | Lateral Zone 2 | Central Zone 1 |  |  |  | <.0001* |
| Mg | Central Zone 2 | Central Zone 1 |  |  |  | 0.0003* |
| Mg | Lateral Zone 3 | Central Zone 2 |  |  |  | 0.0002* |
| Mg | Lateral Zone 3 | Lateral Zone 1 |  |  |  | 0.0001* |
| Mg | Lateral Zone 4 | Central Zone 2 |  |  |  | 0.0004* |
| Mg | Lateral Zone 4 | Lateral Zone 1 |  |  |  | 0.0002* |
| Mg | Lateral Zone 3 | Central Zone 4 |  |  |  | 0.0015* |
| Mg | Lateral Zone 2 | Lateral Zone 1 |  |  |  | 0.0010* |
| Mg | Lateral Zone 1 | Central Zone 1 |  |  |  | 0.0049* |
| Mg | Lateral Zone 3 | Central Zone 3 |  |  |  | 0.0055* |
| Mg | Lateral Zone 4 | Central Zone 4 |  |  |  | 0.0055* |
| Mg | Lateral Zone 2 | Central Zone 2 |  |  |  | 0.0062* |
| Mg | Lateral Zone 4 | Central Zone 3 |  |  |  | 0.0226* |
| Mg | Marginal II Zone 3 | Marginal I Zone 2 |  |  |  | 0.0072* |
| Mg | Central Zone 3 | Central Zone 2 |  |  |  | 0.0641 |
| Mg | Marginal II Zone 3 | Marginal I Zone 1 |  |  |  | 0.0084* |
| Mg | Marginal II Zone 3 | Marginal I Zone 3 |  |  |  | 0.0111* |
| Mg | Marginal II Zone 2 | Marginal I Zone 1 |  |  |  | 0.0120* |
| Mg | Lateral Zone 3 | Lateral Zone 2 |  |  |  | 0.0508 |
| Mg | Marginal II Zone 2 | Marginal I Zone 2 |  |  |  | 0.0217* |
| Mg | Marginal II Zone 1 | Marginal I Zone 1 |  |  |  | 0.0163* |
| Mg | Lateral Zone 2 | Central Zone 4 |  |  |  | 0.1062 |
| Mg | Marginal II Zone 1 | Marginal I Zone 2 |  |  |  | 0.0261* |
| Mg | Central Zone 4 | Central Zone 2 |  |  |  | 0.1327 |
| Mg | Marginal II Zone 3 | Marginal I Zone 4 |  |  |  | 0.0519 |
| Mg | Marginal II Zone 2 | Marginal I Zone 3 |  |  |  | 0.0453* |
| Mg | Lateral Zone 4 | Lateral Zone 2 |  |  |  | 0.1129 |
| Mg | Marginal II Zone 1 | Marginal I Zone 3 |  |  |  | 0.0442* |
| Mg | Marginal II Zone 3 | Marginal II Zone 1 |  |  |  | 0.1309 |
| Mg | Lateral Zone 2 | Central Zone 3 |  |  |  | 0.2465 |
| Mg | Marginal I Zone 3 | Marginal I Zone 1 |  |  |  | 0.0730 |
| Mg | Marginal II Zone 2 | Marginal I Zone 4 |  |  |  | 0.1508 |
| Mg | Marginal II Zone 3 | Central Zone 1 |  |  |  | 0.2597 |
| Mg | Marginal II Zone 1 | Marginal I Zone 4 |  |  |  | 0.1391 |
| Mg | Marginal II Zone 2 | Marginal II Zone 1 |  |  |  | 0.2883 |
| Mg | Marginal I Zone 2 | Marginal I Zone 1 |  |  |  | 0.2540 |
| Mg | Marginal I Zone 4 | Marginal I Zone 1 |  |  |  | 0.2302 |
| Mg | Marginal II Zone 4 | Marginal I Zone 1 |  |  |  | 0.2302 |
| Mg | Marginal II Zone 3 | Marginal II Zone 2 |  |  |  | 0.4274 |
| Mg | Marginal I Zone 3 | Marginal I Zone 2 |  |  |  | 0.4047 |
| Mg | Marginal II Zone 2 | Central Zone 1 |  |  |  | 0.7552 |
| Mg | Marginal I Zone 4 | Marginal I Zone 2 |  |  |  | 0.7309 |
| Mg | Marginal II Zone 4 | Marginal I Zone 2 |  |  |  | 0.7309 |
| Mg | Marginal II Zone 3 | Lateral Zone 1 |  |  |  | 1.0000 |
| Mg | Marginal II Zone 4 | Marginal I Zone 4 |  |  |  | 1.0000 |
| Mg | Marginal I Zone 4 | Marginal I Zone 3 |  |  |  | 0.8164 |
| Mg | Marginal II Zone 4 | Marginal I Zone 3 |  |  |  | 0.8164 |
| Mg | Marginal II Zone 2 | Lateral Zone 1 |  |  |  | 0.5387 |
| Mg | Lateral Zone 4 | Lateral Zone 3 |  |  |  | 0.5351 |
| Mg | Marginal II Zone 3 | Central Zone 2 |  |  |  | 0.4868 |
| Mg | Central Zone 4 | Central Zone 3 |  |  |  | 0.4763 |
| Mg | Marginal II Zone 4 | Marginal II Zone 1 |  |  |  | 0.1391 |
| Mg | Marginal II Zone 4 | Marginal II Zone 2 |  |  |  | 0.1508 |
| Mg | Lateral Zone 1 | Central Zone 2 |  |  |  | 0.2031 |
| Mg | Marginal II Zone 3 | Central Zone 4 |  |  |  | 0.1426 |
| Mg | Marginal II Zone 1 | Central Zone 1 |  |  |  | 0.1426 |
| Mg | Marginal II Zone 4 | Marginal II Zone 3 |  |  |  | 0.0519 |
| Mg | Marginal II Zone 2 | Central Zone 2 |  |  |  | 0.0980 |
| Mg | Marginal II Zone 3 | Central Zone 3 |  |  |  | 0.0886 |
| Mg | Marginal II Zone 3 | Lateral Zone 2 |  |  |  | 0.0128* |
| Mg | Marginal I Zone 4 | Lateral Zone 1 |  |  |  | 0.0098* |
| Mg | Marginal I Zone 4 | Lateral Zone 2 |  |  |  | 0.0098* |
| Mg | Marginal I Zone 4 | Lateral Zone 3 |  |  |  | 0.0098* |
| Mg | Marginal I Zone 4 | Lateral Zone 4 |  |  |  | 0.0098* |
| Mg | Marginal II Zone 4 | Lateral Zone 1 |  |  |  | 0.0098* |
| Mg | Marginal II Zone 4 | Lateral Zone 2 |  |  |  | 0.0098* |
| Mg | Marginal II Zone 4 | Lateral Zone 3 |  |  |  | 0.0098* |
| Mg | Marginal II Zone 4 | Lateral Zone 4 |  |  |  | 0.0098* |
| Mg | Lateral Zone 1 | Central Zone 4 |  |  |  | 0.0128* |
| Mg | Marginal II Zone 2 | Central Zone 4 |  |  |  | 0.0102* |
| Mg | Marginal II Zone 1 | Lateral Zone 1 |  |  |  | 0.0018* |
| Mg | Marginal II Zone 3 | Lateral Zone 4 |  |  |  | 0.0019* |
| Mg | Marginal II Zone 2 | Central Zone 3 |  |  |  | 0.0043* |
| Mg | Marginal I Zone 1 | Lateral Zone 1 |  |  |  | 0.0014* |
| Mg | Marginal I Zone 1 | Lateral Zone 2 |  |  |  | 0.0014* |
| Mg | Marginal I Zone 1 | Lateral Zone 3 |  |  |  | 0.0014* |
| Mg | Marginal I Zone 1 | Lateral Zone 4 |  |  |  | 0.0014* |
| Mg | Marginal II Zone 2 | Lateral Zone 2 |  |  |  | 0.0009* |
| Mg | Marginal II Zone 3 | Lateral Zone 3 |  |  |  | 0.0009* |
| Mg | Lateral Zone 1 | Central Zone 3 |  |  |  | 0.0032* |
| Mg | Marginal I Zone 4 | Central Zone 1 |  |  |  | 0.0077* |
| Mg | Marginal I Zone 4 | Central Zone 2 |  |  |  | 0.0077* |
| Mg | Marginal I Zone 4 | Central Zone 3 |  |  |  | 0.0077* |
| Mg | Marginal I Zone 4 | Central Zone 4 |  |  |  | 0.0076* |
| Mg | Marginal II Zone 4 | Central Zone 1 |  |  |  | 0.0077* |
| Mg | Marginal II Zone 4 | Central Zone 2 |  |  |  | 0.0077* |
| Mg | Marginal II Zone 4 | Central Zone 3 |  |  |  | 0.0077* |
| Mg | Marginal II Zone 4 | Central Zone 4 |  |  |  | 0.0076* |
| Mg | Marginal II Zone 2 | Lateral Zone 4 |  |  |  | 0.0004* |
| Mg | Marginal I Zone 2 | Lateral Zone 1 |  |  |  | 0.0003* |
| Mg | Marginal I Zone 2 | Lateral Zone 2 |  |  |  | 0.0003* |
| Mg | Marginal I Zone 2 | Lateral Zone 3 |  |  |  | 0.0003* |
| Mg | Marginal I Zone 2 | Lateral Zone 4 |  |  |  | 0.0003* |
| Mg | Marginal I Zone 3 | Lateral Zone 1 |  |  |  | 0.0003* |
| Mg | Marginal I Zone 3 | Lateral Zone 2 |  |  |  | 0.0003* |
| Mg | Marginal I Zone 3 | Lateral Zone 3 |  |  |  | 0.0003* |
| Mg | Marginal I Zone 3 | Lateral Zone 4 |  |  |  | 0.0003* |
| Mg | Marginal II Zone 2 | Lateral Zone 3 |  |  |  | 0.0003* |
| Mg | Marginal II Zone 1 | Lateral Zone 2 |  |  |  | 0.0002* |
| Mg | Marginal II Zone 1 | Central Zone 2 |  |  |  | 0.0008* |
| Mg | Marginal I Zone 1 | Central Zone 1 |  |  |  | 0.0009* |
| Mg | Marginal I Zone 1 | Central Zone 2 |  |  |  | 0.0009* |
| Mg | Marginal I Zone 1 | Central Zone 3 |  |  |  | 0.0009* |
| Mg | Marginal I Zone 1 | Central Zone 4 |  |  |  | 0.0009* |
| Mg | Marginal II Zone 1 | Lateral Zone 3 |  |  |  | <.0001* |
| Mg | Marginal II Zone 1 | Lateral Zone 4 |  |  |  | <.0001* |
| Mg | Marginal II Zone 1 | Central Zone 4 |  |  |  | 0.0002* |
| Mg | Marginal I Zone 2 | Central Zone 1 |  |  |  | 0.0002* |
| Mg | Marginal I Zone 2 | Central Zone 2 |  |  |  | 0.0002* |
| Mg | Marginal I Zone 2 | Central Zone 3 |  |  |  | 0.0002* |
| Mg | Marginal I Zone 2 | Central Zone 4 |  |  |  | 0.0002* |
| Mg | Marginal I Zone 3 | Central Zone 1 |  |  |  | 0.0002* |
| Mg | Marginal I Zone 3 | Central Zone 2 |  |  |  | 0.0002* |
| Mg | Marginal I Zone 3 | Central Zone 3 |  |  |  | 0.0002* |
| Mg | Marginal I Zone 3 | Central Zone 4 |  |  |  | 0.0002* |
| Mg | Marginal II Zone 1 | Central Zone 3 |  |  |  | 0.0001* |
| Na | Marginal I Zone 2 | Central Zone 1 | 91.3838 | 15 | <.0001* | <.0001* |
| Na | Marginal I Zone 3 | Central Zone 1 |  |  |  | <.0001* |
| Na | Marginal II Zone 2 | Central Zone 1 |  |  |  | <.0001* |
| Na | Marginal II Zone 3 | Central Zone 1 |  |  |  | <.0001* |
| Na | Marginal II Zone 3 | Central Zone 2 |  |  |  | <.0001* |
| Na | Marginal I Zone 3 | Central Zone 2 |  |  |  | 0.0001* |
| Na | Central Zone 3 | Central Zone 1 |  |  |  | 0.0004* |
| Na | Marginal II Zone 3 | Lateral Zone 1 |  |  |  | <.0001* |
| Na | Marginal II Zone 3 | Marginal II Zone 1 |  |  |  | <.0001* |
| Na | Marginal II Zone 2 | Marginal II Zone 1 |  |  |  | 0.0001* |
| Na | Marginal II Zone 3 | Marginal I Zone 1 |  |  |  | 0.0001* |
| Na | Marginal I Zone 3 | Marginal I Zone 1 |  |  |  | 0.0001* |
| Na | Marginal I Zone 4 | Central Zone 1 |  |  |  | 0.0005* |
| Na | Marginal II Zone 4 | Central Zone 1 |  |  |  | 0.0005* |
| Na | Marginal I Zone 3 | Lateral Zone 1 |  |  |  | 0.0003* |
| Na | Lateral Zone 3 | Central Zone 2 |  |  |  | 0.0013* |
| Na | Central Zone 2 | Central Zone 1 |  |  |  | 0.0022* |
| Na | Central Zone 4 | Central Zone 1 |  |  |  | 0.0019* |
| Na | Lateral Zone 2 | Central Zone 1 |  |  |  | 0.0019* |
| Na | Lateral Zone 3 | Central Zone 1 |  |  |  | 0.0019* |
| Na | Marginal II Zone 4 | Central Zone 2 |  |  |  | 0.0022* |
| Na | Marginal II Zone 2 | Marginal I Zone 1 |  |  |  | 0.0006* |
| Na | Marginal I Zone 4 | Central Zone 2 |  |  |  | 0.0036* |
| Na | Central Zone 3 | Central Zone 2 |  |  |  | 0.0068* |
| Na | Lateral Zone 2 | Central Zone 2 |  |  |  | 0.0050* |
| Na | Marginal II Zone 2 | Central Zone 2 |  |  |  | 0.0045* |
| Na | Lateral Zone 4 | Central Zone 2 |  |  |  | 0.0048* |
| Na | Marginal II Zone 1 | Central Zone 1 |  |  |  | 0.0043* |
| Na | Marginal II Zone 3 | Central Zone 3 |  |  |  | 0.0043* |
| Na | Marginal II Zone 3 | Central Zone 4 |  |  |  | 0.0023* |
| Na | Lateral Zone 3 | Lateral Zone 1 |  |  |  | 0.0029* |
| Na | Lateral Zone 3 | Central Zone 3 |  |  |  | 0.0058* |
| Na | Marginal II Zone 4 | Marginal II Zone 1 |  |  |  | 0.0011* |
| Na | Lateral Zone 1 | Central Zone 1 |  |  |  | 0.0068* |
| Na | Marginal I Zone 2 | Marginal I Zone 1 |  |  |  | 0.0031* |
| Na | Marginal II Zone 2 | Lateral Zone 1 |  |  |  | 0.0042* |
| Na | Lateral Zone 3 | Central Zone 4 |  |  |  | 0.0051* |
| Na | Lateral Zone 4 | Central Zone 1 |  |  |  | 0.0071* |
| Na | Marginal II Zone 3 | Marginal II Zone 2 |  |  |  | 0.0039* |
| Na | Marginal I Zone 4 | Marginal I Zone 1 |  |  |  | 0.0022* |
| Na | Marginal II Zone 4 | Marginal I Zone 1 |  |  |  | 0.0022* |
| Na | Marginal II Zone 4 | Lateral Zone 1 |  |  |  | 0.0043* |
| Na | Lateral Zone 2 | Lateral Zone 1 |  |  |  | 0.0086* |
| Na | Marginal I Zone 4 | Lateral Zone 1 |  |  |  | 0.0057* |
| Na | Lateral Zone 4 | Lateral Zone 1 |  |  |  | 0.0092* |
| Na | Lateral Zone 4 | Central Zone 3 |  |  |  | 0.0213* |
| Na | Marginal II Zone 3 | Marginal I Zone 2 |  |  |  | 0.0126* |
| Na | Marginal I Zone 3 | Central Zone 4 |  |  |  | 0.0151* |
| Na | Marginal I Zone 2 | Central Zone 2 |  |  |  | 0.0338* |
| Na | Marginal I Zone 2 | Lateral Zone 1 |  |  |  | 0.0210* |
| Na | Lateral Zone 4 | Central Zone 4 |  |  |  | 0.0192* |
| Na | Marginal I Zone 3 | Marginal I Zone 2 |  |  |  | 0.0215* |
| Na | Marginal II Zone 4 | Central Zone 4 |  |  |  | 0.0351* |
| Na | Central Zone 4 | Central Zone 2 |  |  |  | 0.0739 |
| Na | Marginal I Zone 1 | Central Zone 1 |  |  |  | 0.0654 |
| Na | Marginal II Zone 4 | Marginal I Zone 2 |  |  |  | 0.0307* |
| Na | Marginal I Zone 3 | Central Zone 3 |  |  |  | 0.0733 |
| Na | Marginal I Zone 4 | Marginal I Zone 2 |  |  |  | 0.0394* |
| Na | Lateral Zone 2 | Central Zone 4 |  |  |  | 0.0885 |
| Na | Marginal I Zone 4 | Central Zone 4 |  |  |  | 0.0678 |
| Na | Marginal II Zone 4 | Central Zone 3 |  |  |  | 0.1105 |
| Na | Lateral Zone 3 | Lateral Zone 2 |  |  |  | 0.1260 |
| Na | Marginal II Zone 4 | Marginal II Zone 2 |  |  |  | 0.0973 |
| Na | Lateral Zone 2 | Central Zone 3 |  |  |  | 0.1960 |
| Na | Marginal I Zone 4 | Central Zone 3 |  |  |  | 0.1855 |
| Na | Lateral Zone 4 | Lateral Zone 2 |  |  |  | 0.1563 |
| Na | Marginal II Zone 3 | Lateral Zone 2 |  |  |  | 0.4060 |
| Na | Marginal II Zone 1 | Marginal I Zone 1 |  |  |  | 0.4116 |
| Na | Marginal II Zone 3 | Marginal I Zone 3 |  |  |  | 0.4500 |
| Na | Marginal II Zone 2 | Central Zone 4 |  |  |  | 0.5181 |
| Na | Marginal II Zone 2 | Marginal I Zone 2 |  |  |  | 0.5766 |
| Na | Marginal I Zone 2 | Central Zone 4 |  |  |  | 0.6009 |
| Na | Marginal II Zone 3 | Marginal I Zone 4 |  |  |  | 0.5804 |
| Na | Marginal I Zone 4 | Lateral Zone 2 |  |  |  | 0.6065 |
| Na | Marginal I Zone 3 | Lateral Zone 2 |  |  |  | 0.6891 |
| Na | Marginal II Zone 4 | Lateral Zone 2 |  |  |  | 0.6734 |
| Na | Marginal II Zone 4 | Marginal I Zone 3 |  |  |  | 0.9199 |
| Na | Marginal II Zone 4 | Marginal I Zone 4 |  |  |  | 0.9362 |
| Na | Marginal I Zone 4 | Marginal I Zone 3 |  |  |  | 0.9199 |
| Na | Marginal II Zone 4 | Marginal II Zone 3 |  |  |  | 0.8802 |
| Na | Lateral Zone 4 | Lateral Zone 3 |  |  |  | 0.8691 |
| Na | Marginal II Zone 2 | Central Zone 3 |  |  |  | 0.7555 |
| Na | Marginal I Zone 4 | Lateral Zone 4 |  |  |  | 0.6255 |
| Na | Marginal I Zone 2 | Central Zone 3 |  |  |  | 0.6404 |
| Na | Marginal II Zone 4 | Lateral Zone 4 |  |  |  | 0.4808 |
| Na | Marginal I Zone 4 | Lateral Zone 3 |  |  |  | 0.3736 |
| Na | Marginal I Zone 3 | Lateral Zone 4 |  |  |  | 0.3787 |
| Na | Lateral Zone 1 | Central Zone 2 |  |  |  | 0.4299 |
| Na | Central Zone 4 | Central Zone 3 |  |  |  | 0.4067 |
| Na | Marginal II Zone 4 | Lateral Zone 3 |  |  |  | 0.3026 |
| Na | Marginal II Zone 3 | Lateral Zone 4 |  |  |  | 0.2751 |
| Na | Marginal II Zone 2 | Marginal I Zone 4 |  |  |  | 0.2090 |
| Na | Marginal I Zone 2 | Lateral Zone 2 |  |  |  | 0.1961 |
| Na | Marginal II Zone 1 | Lateral Zone 1 |  |  |  | 0.1661 |
| Na | Marginal II Zone 2 | Lateral Zone 2 |  |  |  | 0.1661 |
| Na | Marginal I Zone 3 | Lateral Zone 3 |  |  |  | 0.1568 |
| Na | Marginal I Zone 1 | Lateral Zone 1 |  |  |  | 0.1396 |
| Na | Marginal II Zone 3 | Lateral Zone 3 |  |  |  | 0.1316 |
| Na | Marginal I Zone 2 | Lateral Zone 4 |  |  |  | 0.0725 |
| Na | Lateral Zone 1 | Central Zone 4 |  |  |  | 0.0690 |
| Na | Marginal I Zone 1 | Central Zone 2 |  |  |  | 0.0887 |
| Na | Marginal II Zone 2 | Lateral Zone 4 |  |  |  | 0.0448* |
| Na | Marginal I Zone 1 | Central Zone 4 |  |  |  | 0.0392* |
| Na | Marginal II Zone 2 | Marginal I Zone 3 |  |  |  | 0.0302* |
| Na | Marginal I Zone 2 | Lateral Zone 3 |  |  |  | 0.0337* |
| Na | Marginal I Zone 1 | Lateral Zone 4 |  |  |  | 0.0221* |
| Na | Marginal II Zone 1 | Lateral Zone 4 |  |  |  | 0.0221* |
| Na | Marginal II Zone 2 | Lateral Zone 3 |  |  |  | 0.0178* |
| Na | Marginal II Zone 1 | Central Zone 2 |  |  |  | 0.0281* |
| Na | Marginal I Zone 1 | Lateral Zone 2 |  |  |  | 0.0151* |
| Na | Marginal II Zone 1 | Central Zone 4 |  |  |  | 0.0106* |
| Na | Marginal I Zone 1 | Lateral Zone 3 |  |  |  | 0.0074* |
| Na | Marginal II Zone 1 | Lateral Zone 2 |  |  |  | 0.0074* |
| Na | Marginal II Zone 1 | Lateral Zone 3 |  |  |  | 0.0074* |
| Na | Lateral Zone 1 | Central Zone 3 |  |  |  | 0.0104* |
| Na | Marginal I Zone 1 | Central Zone 3 |  |  |  | 0.0070* |
| Na | Marginal II Zone 1 | Marginal I Zone 4 |  |  |  | 0.0011* |
| Na | Marginal II Zone 1 | Central Zone 3 |  |  |  | 0.0018* |
| Na | Marginal II Zone 1 | Marginal I Zone 2 |  |  |  | 0.0002* |
| Na | Marginal II Zone 1 | Marginal I Zone 3 |  |  |  | <.0001 |
| P | Lateral Zone 3 | Central Zone 1 | 66.0764 | 15 | <.0001* | 0.0002* |
| P | Lateral Zone 4 | Central Zone 1 |  |  |  | 0.0010* |
| P | Lateral Zone 3 | Central Zone 2 |  |  |  | 0.0021* |
| P | Central Zone 4 | Central Zone 1 |  |  |  | 0.0039* |
| P | Central Zone 3 | Central Zone 1 |  |  |  | 0.0068* |
| P | Lateral Zone 3 | Central Zone 3 |  |  |  | 0.0050* |
| P | Lateral Zone 3 | Central Zone 4 |  |  |  | 0.0063* |
| P | Lateral Zone 4 | Central Zone 2 |  |  |  | 0.0092* |
| P | Lateral Zone 1 | Central Zone 1 |  |  |  | 0.0149* |
| P | Lateral Zone 4 | Central Zone 3 |  |  |  | 0.0235* |
| P | Lateral Zone 2 | Central Zone 1 |  |  |  | 0.0275* |
| P | Central Zone 2 | Central Zone 1 |  |  |  | 0.0382* |
| P | Lateral Zone 4 | Central Zone 4 |  |  |  | 0.0253* |
| P | Lateral Zone 3 | Lateral Zone 2 |  |  |  | 0.0230* |
| P | Lateral Zone 3 | Lateral Zone 1 |  |  |  | 0.0209* |
| P | Lateral Zone 4 | Lateral Zone 1 |  |  |  | 0.0529 |
| P | Lateral Zone 1 | Central Zone 2 |  |  |  | 0.1030 |
| P | Lateral Zone 2 | Central Zone 2 |  |  |  | 0.1148 |
| P | Lateral Zone 4 | Lateral Zone 2 |  |  |  | 0.1050 |
| P | Lateral Zone 2 | Central Zone 3 |  |  |  | 0.1542 |
| P | Marginal II Zone 2 | Marginal I Zone 2 |  |  |  | 0.0200* |
| P | Marginal II Zone 3 | Marginal I Zone 2 |  |  |  | 0.0200* |
| P | Lateral Zone 2 | Central Zone 4 |  |  |  | 0.1968 |
| P | Central Zone 3 | Central Zone 2 |  |  |  | 0.2481 |
| P | Central Zone 4 | Central Zone 2 |  |  |  | 0.2547 |
| P | Marginal II Zone 2 | Marginal II Zone 1 |  |  |  | 0.0304* |
| P | Marginal II Zone 3 | Marginal I Zone 3 |  |  |  | 0.0304* |
| P | Marginal II Zone 3 | Marginal II Zone 1 |  |  |  | 0.0304* |
| P | Marginal II Zone 3 | Marginal II Zone 2 |  |  |  | 0.0304* |
| P | Lateral Zone 2 | Lateral Zone 1 |  |  |  | 0.2265 |
| P | Marginal II Zone 2 | Marginal I Zone 3 |  |  |  | 0.0606 |
| P | Marginal II Zone 1 | Marginal I Zone 1 |  |  |  | 0.0518 |
| P | Marginal II Zone 2 | Marginal I Zone 1 |  |  |  | 0.0518 |
| P | Marginal II Zone 3 | Marginal I Zone 1 |  |  |  | 0.0518 |
| P | Marginal II Zone 4 | Marginal I Zone 2 |  |  |  | 0.0814 |
| P | Marginal II Zone 1 | Marginal I Zone 4 |  |  |  | 0.1002 |
| P | Marginal II Zone 2 | Marginal I Zone 4 |  |  |  | 0.1002 |
| P | Marginal II Zone 3 | Marginal I Zone 4 |  |  |  | 0.1002 |
| P | Marginal II Zone 4 | Marginal I Zone 3 |  |  |  | 0.1052 |
| P | Marginal II Zone 4 | Marginal II Zone 1 |  |  |  | 0.1052 |
| P | Marginal II Zone 4 | Marginal II Zone 2 |  |  |  | 0.1052 |
| P | Marginal II Zone 1 | Marginal I Zone 2 |  |  |  | 0.1779 |
| P | Lateral Zone 1 | Central Zone 4 |  |  |  | 0.4648 |
| P | Marginal II Zone 4 | Marginal I Zone 1 |  |  |  | 0.1489 |
| P | Marginal I Zone 3 | Marginal I Zone 2 |  |  |  | 0.2703 |
| P | Lateral Zone 1 | Central Zone 3 |  |  |  | 0.5392 |
| P | Marginal II Zone 1 | Marginal I Zone 3 |  |  |  | 0.3123 |
| P | Marginal II Zone 4 | Marginal I Zone 4 |  |  |  | 0.2207 |
| P | Marginal I Zone 3 | Marginal I Zone 1 |  |  |  | 0.5959 |
| P | Marginal I Zone 2 | Marginal I Zone 1 |  |  |  | 1.0000 |
| P | Marginal I Zone 4 | Marginal I Zone 2 |  |  |  | 1.0000 |
| P | Central Zone 4 | Central Zone 3 |  |  |  | 0.9080 |
| P | Marginal I Zone 4 | Marginal I Zone 1 |  |  |  | 0.7671 |
| P | Marginal II Zone 4 | Marginal II Zone 3 |  |  |  | 0.2472 |
| P | Marginal I Zone 4 | Marginal I Zone 3 |  |  |  | 0.1002 |
| P | Lateral Zone 4 | Lateral Zone 3 |  |  |  | 0.2812 |
| P | Marginal II Zone 4 | Lateral Zone 2 |  |  |  | 0.3818 |
| P | Marginal II Zone 2 | Lateral Zone 2 |  |  |  | 0.2215 |
| P | Marginal II Zone 3 | Lateral Zone 2 |  |  |  | 0.2215 |
| P | Marginal II Zone 4 | Central Zone 1 |  |  |  | 0.3445 |
| P | Marginal II Zone 4 | Lateral Zone 1 |  |  |  | 0.1700 |
| P | Marginal II Zone 4 | Lateral Zone 4 |  |  |  | 0.1704 |
| P | Marginal II Zone 3 | Central Zone 1 |  |  |  | 0.1869 |
| P | Marginal II Zone 4 | Lateral Zone 3 |  |  |  | 0.0929 |
| P | Marginal II Zone 2 | Central Zone 1 |  |  |  | 0.1600 |
| P | Marginal II Zone 1 | Lateral Zone 1 |  |  |  | 0.0598 |
| P | Marginal II Zone 2 | Lateral Zone 1 |  |  |  | 0.0598 |
| P | Marginal II Zone 3 | Lateral Zone 1 |  |  |  | 0.0598 |
| P | Marginal II Zone 3 | Lateral Zone 4 |  |  |  | 0.0600 |
| P | Marginal II Zone 4 | Central Zone 2 |  |  |  | 0.2312 |
| P | Marginal I Zone 4 | Lateral Zone 2 |  |  |  | 0.1306 |
| P | Marginal II Zone 2 | Lateral Zone 4 |  |  |  | 0.0452* |
| P | Marginal I Zone 3 | Lateral Zone 2 |  |  |  | 0.0628 |
| P | Marginal I Zone 1 | Lateral Zone 3 |  |  |  | 0.0291* |
| P | Marginal II Zone 3 | Central Zone 2 |  |  |  | 0.0968 |
| P | Marginal II Zone 1 | Lateral Zone 3 |  |  |  | 0.0222* |
| P | Marginal II Zone 2 | Lateral Zone 3 |  |  |  | 0.0222* |
| P | Marginal II Zone 3 | Lateral Zone 3 |  |  |  | 0.0222* |
| P | Marginal I Zone 4 | Lateral Zone 4 |  |  |  | 0.0539 |
| P | Marginal I Zone 3 | Lateral Zone 3 |  |  |  | 0.0186* |
| P | Marginal I Zone 4 | Lateral Zone 3 |  |  |  | 0.0377* |
| P | Marginal II Zone 1 | Central Zone 1 |  |  |  | 0.0806 |
| P | Marginal II Zone 3 | Central Zone 3 |  |  |  | 0.0806 |
| P | Marginal II Zone 1 | Lateral Zone 2 |  |  |  | 0.0382* |
| P | Marginal II Zone 4 | Central Zone 3 |  |  |  | 0.1472 |
| P | Marginal II Zone 4 | Central Zone 4 |  |  |  | 0.1258 |
| P | Marginal I Zone 1 | Lateral Zone 4 |  |  |  | 0.0251* |
| P | Marginal I Zone 3 | Lateral Zone 1 |  |  |  | 0.0179* |
| P | Marginal I Zone 3 | Lateral Zone 4 |  |  |  | 0.0180* |
| P | Marginal II Zone 1 | Lateral Zone 4 |  |  |  | 0.0180* |
| P | Marginal II Zone 3 | Central Zone 4 |  |  |  | 0.0540 |
| P | Marginal I Zone 1 | Lateral Zone 2 |  |  |  | 0.0374* |
| P | Marginal I Zone 4 | Lateral Zone 1 |  |  |  | 0.0352* |
| P | Marginal I Zone 2 | Lateral Zone 2 |  |  |  | 0.0141* |
| P | Marginal I Zone 2 | Lateral Zone 3 |  |  |  | 0.0046* |
| P | Marginal I Zone 1 | Lateral Zone 1 |  |  |  | 0.0114* |
| P | Marginal I Zone 3 | Central Zone 1 |  |  |  | 0.0370* |
| P | Marginal II Zone 2 | Central Zone 2 |  |  |  | 0.0368* |
| P | Marginal I Zone 2 | Lateral Zone 4 |  |  |  | 0.0044* |
| P | Marginal I Zone 2 | Lateral Zone 1 |  |  |  | 0.0037* |
| P | Marginal II Zone 1 | Central Zone 2 |  |  |  | 0.0192* |
| P | Marginal II Zone 1 | Central Zone 3 |  |  |  | 0.0192* |
| P | Marginal II Zone 2 | Central Zone 3 |  |  |  | 0.0192* |
| P | Marginal I Zone 4 | Central Zone 1 |  |  |  | 0.0507 |
| P | Marginal I Zone 1 | Central Zone 1 |  |  |  | 0.0237* |
| P | Marginal II Zone 1 | Central Zone 4 |  |  |  | 0.0082* |
| P | Marginal II Zone 2 | Central Zone 4 |  |  |  | 0.0082* |
| P | Marginal I Zone 4 | Central Zone 4 |  |  |  | 0.0282* |
| P | Marginal I Zone 1 | Central Zone 4 |  |  |  | 0.0110* |
| P | Marginal I Zone 1 | Central Zone 2 |  |  |  | 0.0138* |
| P | Marginal I Zone 1 | Central Zone 3 |  |  |  | 0.0138* |
| P | Marginal I Zone 2 | Central Zone 1 |  |  |  | 0.0052* |
| P | Marginal I Zone 3 | Central Zone 2 |  |  |  | 0.0073* |
| P | Marginal I Zone 3 | Central Zone 3 |  |  |  | 0.0073* |
| P | Marginal I Zone 4 | Central Zone 2 |  |  |  | 0.0274* |
| P | Marginal I Zone 4 | Central Zone 3 |  |  |  | 0.0274* |
| P | Marginal I Zone 3 | Central Zone 4 |  |  |  | 0.0048* |
| P | Marginal I Zone 2 | Central Zone 2 |  |  |  | 0.0025* |
| P | Marginal I Zone 2 | Central Zone 3 |  |  |  | 0.0025* |
| P | Marginal I Zone 2 | Central Zone 4 |  |  |  | 0.0017* |
| S | Central Zone 2 | Central Zone 1 | 52.4172 | 15 | <.0001* | 0.0004* |
| S | Central Zone 3 | Central Zone 1 |  |  |  | 0.0004* |
| S | Central Zone 4 | Central Zone 1 |  |  |  | 0.0006* |
| S | Marginal II Zone 3 | Lateral Zone 1 |  |  |  | 0.0039* |
| S | Marginal I Zone 3 | Marginal I Zone 1 |  |  |  | 0.0074* |
| S | Marginal II Zone 4 | Lateral Zone 1 |  |  |  | 0.0080* |
| S | Marginal II Zone 3 | Central Zone 1 |  |  |  | 0.0113* |
| S | Marginal I Zone 3 | Lateral Zone 1 |  |  |  | 0.0184* |
| S | Marginal II Zone 2 | Marginal I Zone 1 |  |  |  | 0.0118* |
| S | Marginal II Zone 3 | Marginal I Zone 1 |  |  |  | 0.0118* |
| S | Marginal II Zone 4 | Marginal I Zone 1 |  |  |  | 0.0118* |
| S | Marginal II Zone 2 | Lateral Zone 1 |  |  |  | 0.0216* |
| S | Marginal II Zone 4 | Central Zone 1 |  |  |  | 0.0216* |
| S | Marginal I Zone 4 | Marginal I Zone 1 |  |  |  | 0.0169* |
| S | Lateral Zone 3 | Lateral Zone 1 |  |  |  | 0.0341* |
| S | Marginal II Zone 3 | Marginal II Zone 1 |  |  |  | 0.0131* |
| S | Marginal I Zone 4 | Lateral Zone 1 |  |  |  | 0.0292* |
| S | Central Zone 3 | Central Zone 2 |  |  |  | 0.0423* |
| S | Lateral Zone 2 | Lateral Zone 1 |  |  |  | 0.0637 |
| S | Marginal II Zone 4 | Marginal II Zone 1 |  |  |  | 0.0306* |
| S | Lateral Zone 4 | Lateral Zone 1 |  |  |  | 0.0677 |
| S | Marginal I Zone 2 | Lateral Zone 1 |  |  |  | 0.0922 |
| S | Marginal I Zone 3 | Central Zone 1 |  |  |  | 0.0922 |
| S | Marginal II Zone 2 | Central Zone 1 |  |  |  | 0.0875 |
| S | Marginal II Zone 3 | Marginal I Zone 2 |  |  |  | 0.0814 |
| S | Marginal II Zone 3 | Lateral Zone 2 |  |  |  | 0.0987 |
| S | Central Zone 4 | Central Zone 2 |  |  |  | 0.1358 |
| S | Marginal I Zone 2 | Marginal I Zone 1 |  |  |  | 0.1278 |
| S | Marginal II Zone 4 | Marginal I Zone 2 |  |  |  | 0.1376 |
| S | Marginal II Zone 4 | Lateral Zone 2 |  |  |  | 0.1753 |
| S | Marginal II Zone 2 | Marginal II Zone 1 |  |  |  | 0.1282 |
| S | Marginal I Zone 4 | Central Zone 1 |  |  |  | 0.2159 |
| S | Lateral Zone 3 | Central Zone 1 |  |  |  | 0.2510 |
| S | Marginal II Zone 3 | Marginal II Zone 2 |  |  |  | 0.1735 |
| S | Marginal II Zone 4 | Marginal II Zone 2 |  |  |  | 0.1735 |
| S | Marginal I Zone 3 | Marginal I Zone 2 |  |  |  | 0.2701 |
| S | Marginal II Zone 3 | Lateral Zone 4 |  |  |  | 0.2298 |
| S | Marginal I Zone 4 | Marginal I Zone 2 |  |  |  | 0.2725 |
| S | Lateral Zone 3 | Lateral Zone 2 |  |  |  | 0.3314 |
| S | Lateral Zone 4 | Central Zone 1 |  |  |  | 0.3165 |
| S | Lateral Zone 2 | Central Zone 1 |  |  |  | 0.3772 |
| S | Marginal II Zone 2 | Marginal I Zone 2 |  |  |  | 0.3329 |
| S | Marginal II Zone 3 | Central Zone 2 |  |  |  | 0.3768 |
| S | Marginal II Zone 4 | Lateral Zone 4 |  |  |  | 0.3785 |
| S | Marginal II Zone 3 | Lateral Zone 3 |  |  |  | 0.4437 |
| S | Marginal I Zone 2 | Central Zone 1 |  |  |  | 0.4705 |
| S | Marginal II Zone 1 | Marginal I Zone 1 |  |  |  | 0.4777 |
| S | Marginal II Zone 3 | Marginal I Zone 3 |  |  |  | 0.4777 |
| S | Marginal II Zone 4 | Marginal I Zone 3 |  |  |  | 0.4777 |
| S | Lateral Zone 4 | Lateral Zone 2 |  |  |  | 0.5169 |
| S | Marginal I Zone 4 | Lateral Zone 2 |  |  |  | 0.5169 |
| S | Marginal II Zone 1 | Lateral Zone 1 |  |  |  | 0.5169 |
| S | Marginal II Zone 4 | Central Zone 2 |  |  |  | 0.5553 |
| S | Marginal I Zone 3 | Lateral Zone 2 |  |  |  | 0.5966 |
| S | Marginal II Zone 2 | Lateral Zone 2 |  |  |  | 0.6800 |
| S | Marginal II Zone 4 | Lateral Zone 3 |  |  |  | 0.6800 |
| S | Marginal I Zone 3 | Lateral Zone 4 |  |  |  | 0.6982 |
| S | Marginal I Zone 4 | Lateral Zone 4 |  |  |  | 0.6889 |
| S | Marginal II Zone 2 | Lateral Zone 4 |  |  |  | 0.6889 |
| S | Marginal II Zone 2 | Marginal I Zone 3 |  |  |  | 0.7469 |
| S | Marginal II Zone 3 | Marginal I Zone 4 |  |  |  | 0.8102 |
| S | Marginal II Zone 4 | Marginal I Zone 4 |  |  |  | 0.8102 |
| S | Marginal I Zone 4 | Lateral Zone 3 |  |  |  | 0.8597 |
| S | Lateral Zone 3 | Central Zone 2 |  |  |  | 0.9296 |
| S | Marginal II Zone 2 | Marginal I Zone 4 |  |  |  | 0.9362 |
| S | Marginal I Zone 1 | Lateral Zone 1 |  |  |  | 0.9616 |
| S | Marginal I Zone 3 | Lateral Zone 3 |  |  |  | 0.9616 |
| S | Marginal I Zone 2 | Lateral Zone 2 |  |  |  | 1.0000 |
| S | Marginal II Zone 3 | Central Zone 4 |  |  |  | 1.0000 |
| S | Marginal II Zone 2 | Lateral Zone 3 |  |  |  | 0.9530 |
| S | Marginal I Zone 4 | Marginal I Zone 3 |  |  |  | 0.7469 |
| S | Marginal II Zone 1 | Central Zone 1 |  |  |  | 0.7234 |
| S | Marginal II Zone 3 | Central Zone 3 |  |  |  | 0.7234 |
| S | Marginal II Zone 4 | Central Zone 4 |  |  |  | 0.6982 |
| S | Lateral Zone 4 | Central Zone 2 |  |  |  | 0.6800 |
| S | Lateral Zone 4 | Lateral Zone 3 |  |  |  | 0.6800 |
| S | Marginal II Zone 4 | Marginal II Zone 3 |  |  |  | 0.5752 |
| S | Marginal II Zone 2 | Central Zone 2 |  |  |  | 0.5553 |
| S | Marginal I Zone 4 | Central Zone 2 |  |  |  | 0.5169 |
| S | Central Zone 4 | Central Zone 3 |  |  |  | 0.4705 |
| S | Marginal I Zone 3 | Central Zone 2 |  |  |  | 0.4705 |
| S | Marginal II Zone 4 | Central Zone 3 |  |  |  | 0.3768 |
| S | Marginal I Zone 2 | Lateral Zone 4 |  |  |  | 0.3329 |
| S | Lateral Zone 3 | Central Zone 4 |  |  |  | 0.3356 |
| S | Marginal I Zone 4 | Central Zone 4 |  |  |  | 0.2725 |
| S | Marginal I Zone 4 | Central Zone 3 |  |  |  | 0.2629 |
| S | Marginal I Zone 2 | Lateral Zone 3 |  |  |  | 0.2290 |
| S | Marginal II Zone 1 | Lateral Zone 4 |  |  |  | 0.1282 |
| S | Marginal II Zone 1 | Marginal I Zone 4 |  |  |  | 0.1282 |
| S | Lateral Zone 2 | Central Zone 2 |  |  |  | 0.2002 |
| S | Marginal II Zone 1 | Marginal I Zone 2 |  |  |  | 0.1376 |
| S | Marginal II Zone 1 | Marginal I Zone 3 |  |  |  | 0.1376 |
| S | Marginal II Zone 1 | Lateral Zone 2 |  |  |  | 0.1407 |
| S | Lateral Zone 4 | Central Zone 4 |  |  |  | 0.1209 |
| S | Lateral Zone 1 | Central Zone 1 |  |  |  | 0.1449 |
| S | Lateral Zone 3 | Central Zone 3 |  |  |  | 0.1449 |
| S | Marginal I Zone 3 | Central Zone 4 |  |  |  | 0.1149 |
| S | Marginal II Zone 2 | Central Zone 4 |  |  |  | 0.0814 |
| S | Marginal II Zone 1 | Lateral Zone 3 |  |  |  | 0.0875 |
| S | Marginal I Zone 1 | Central Zone 1 |  |  |  | 0.0750 |
| S | Marginal I Zone 1 | Lateral Zone 2 |  |  |  | 0.0750 |
| S | Marginal I Zone 3 | Central Zone 3 |  |  |  | 0.0750 |
| S | Marginal I Zone 1 | Lateral Zone 4 |  |  |  | 0.0454* |
| S | Lateral Zone 4 | Central Zone 3 |  |  |  | 0.0449* |
| S | Marginal I Zone 2 | Central Zone 2 |  |  |  | 0.0485* |
| S | Lateral Zone 2 | Central Zone 4 |  |  |  | 0.0386* |
| S | Marginal II Zone 2 | Central Zone 3 |  |  |  | 0.0216* |
| S | Marginal I Zone 2 | Central Zone 4 |  |  |  | 0.0181* |
| S | Marginal I Zone 1 | Lateral Zone 3 |  |  |  | 0.0141* |
| S | Lateral Zone 2 | Central Zone 3 |  |  |  | 0.0081* |
| S | Marginal I Zone 2 | Central Zone 3 |  |  |  | 0.0061* |
| S | Marginal II Zone 1 | Central Zone 4 |  |  |  | 0.0024* |
| S | Marginal II Zone 1 | Central Zone 2 |  |  |  | 0.0018* |
| S | Marginal II Zone 1 | Central Zone 3 |  |  |  | 0.0018* |
| S | Marginal I Zone 1 | Central Zone 4 |  |  |  | 0.0009* |
| S | Lateral Zone 1 | Central Zone 4 |  |  |  | 0.0006* |
| S | Marginal I Zone 1 | Central Zone 2 |  |  |  | 0.0006* |
| S | Marginal I Zone 1 | Central Zone 3 |  |  |  | 0.0006* |
| S | Lateral Zone 1 | Central Zone 2 |  |  |  | 0.0004* |
| S | Lateral Zone 1 | Central Zone 3 |  |  |  | 0.0004* |
| Si | Central Zone 3 | Central Zone 1 | 29.8795 | 14 | 0.0079* | 0.0037* |
| Si | Central Zone 4 | Central Zone 1 |  |  |  | 0.0026* |
| Si | Lateral Zone 3 | Central Zone 1 |  |  |  | 0.0098* |
| Si | Lateral Zone 3 | Lateral Zone 1 |  |  |  | 0.0098* |
| Si | Lateral Zone 4 | Central Zone 1 |  |  |  | 0.0340* |
| Si | Lateral Zone 2 | Central Zone 1 |  |  |  | 0.0526 |
| Si | Central Zone 2 | Central Zone 1 |  |  |  | 0.0575 |
| Si | Lateral Zone 4 | Lateral Zone 1 |  |  |  | 0.0519 |
| Si | Central Zone 3 | Central Zone 2 |  |  |  | 0.0843 |
| Si | Central Zone 4 | Central Zone 2 |  |  |  | 0.0968 |
| Si | Lateral Zone 2 | Lateral Zone 1 |  |  |  | 0.1192 |
| Si | Lateral Zone 3 | Central Zone 2 |  |  |  | 0.2122 |
| Si | Marginal I Zone 3 | Central Zone 1 |  |  |  | 0.2963 |
| Si | Marginal I Zone 4 | Central Zone 1 |  |  |  | 0.2963 |
| Si | Lateral Zone 3 | Lateral Zone 2 |  |  |  | 0.2934 |
| Si | Marginal I Zone 3 | Lateral Zone 1 |  |  |  | 0.4862 |
| Si | Marginal I Zone 4 | Lateral Zone 1 |  |  |  | 0.4862 |
| Si | Lateral Zone 4 | Central Zone 2 |  |  |  | 0.4031 |
| Si | Lateral Zone 4 | Lateral Zone 2 |  |  |  | 0.4031 |
| Si | Marginal I Zone 2 | Central Zone 1 |  |  |  | 0.7277 |
| Si | Marginal I Zone 2 | Lateral Zone 1 |  |  |  | 0.7277 |
| Si | Marginal II Zone 2 | Marginal I Zone 1 |  |  |  | 0.5403 |
| Si | Marginal II Zone 3 | Marginal I Zone 1 |  |  |  | 0.5403 |
| Si | Marginal II Zone 3 | Marginal II Zone 1 |  |  |  | 0.5403 |
| Si | Marginal II Zone 3 | Marginal II Zone 2 |  |  |  | 0.6985 |
| Si | Marginal II Zone 3 | Central Zone 1 |  |  |  | 0.9062 |
| Si | Marginal II Zone 3 | Lateral Zone 1 |  |  |  | 0.9062 |
| Si | Lateral Zone 2 | Central Zone 2 |  |  |  | 0.9476 |
| Si | Lateral Zone 1 | Central Zone 1 |  |  |  | 1.0000 |
| Si | Marginal I Zone 2 | Marginal I Zone 1 |  |  |  | 1.0000 |
| Si | Marginal I Zone 3 | Central Zone 2 |  |  |  | 1.0000 |
| Si | Marginal I Zone 3 | Lateral Zone 2 |  |  |  | 1.0000 |
| Si | Marginal I Zone 3 | Marginal I Zone 1 |  |  |  | 1.0000 |
| Si | Marginal I Zone 3 | Marginal I Zone 2 |  |  |  | 1.0000 |
| Si | Marginal I Zone 4 | Central Zone 2 |  |  |  | 1.0000 |
| Si | Marginal I Zone 4 | Lateral Zone 2 |  |  |  | 1.0000 |
| Si | Marginal I Zone 4 | Marginal I Zone 1 |  |  |  | 1.0000 |
| Si | Marginal I Zone 4 | Marginal I Zone 2 |  |  |  | 1.0000 |
| Si | Marginal I Zone 4 | Marginal I Zone 3 |  |  |  | 1.0000 |
| Si | Marginal II Zone 1 | Marginal I Zone 1 |  |  |  | 1.0000 |
| Si | Marginal II Zone 1 | Marginal I Zone 2 |  |  |  | 1.0000 |
| Si | Marginal II Zone 1 | Marginal I Zone 3 |  |  |  | 1.0000 |
| Si | Marginal II Zone 1 | Marginal I Zone 4 |  |  |  | 1.0000 |
| Si | Marginal II Zone 2 | Central Zone 1 |  |  |  | 1.0000 |
| Si | Marginal II Zone 2 | Marginal I Zone 2 |  |  |  | 1.0000 |
| Si | Marginal II Zone 2 | Marginal II Zone 1 |  |  |  | 1.0000 |
| Si | Marginal II Zone 3 | Marginal I Zone 2 |  |  |  | 1.0000 |
| Si | Marginal II Zone 3 | Marginal I Zone 3 |  |  |  | 1.0000 |
| Si | Marginal II Zone 3 | Marginal I Zone 4 |  |  |  | 1.0000 |
| Si | Central Zone 4 | Central Zone 3 |  |  |  | 0.8273 |
| Si | Marginal II Zone 2 | Marginal I Zone 3 |  |  |  | 0.5403 |
| Si | Marginal II Zone 2 | Marginal I Zone 4 |  |  |  | 0.5403 |
| Si | Lateral Zone 3 | Central Zone 4 |  |  |  | 0.7244 |
| Si | Marginal II Zone 2 | Lateral Zone 1 |  |  |  | 0.7225 |
| Si | Lateral Zone 3 | Central Zone 3 |  |  |  | 0.7241 |
| Si | Lateral Zone 4 | Lateral Zone 3 |  |  |  | 0.6208 |
| Si | Marginal II Zone 3 | Lateral Zone 2 |  |  |  | 0.6217 |
| Si | Lateral Zone 4 | Central Zone 4 |  |  |  | 0.4052 |
| Si | Marginal II Zone 3 | Central Zone 2 |  |  |  | 0.4897 |
| Si | Marginal I Zone 2 | Lateral Zone 4 |  |  |  | 0.4849 |
| Si | Marginal I Zone 3 | Lateral Zone 4 |  |  |  | 0.4849 |
| Si | Marginal I Zone 4 | Lateral Zone 4 |  |  |  | 0.4849 |
| Si | Lateral Zone 4 | Central Zone 3 |  |  |  | 0.3672 |
| Si | Marginal II Zone 2 | Lateral Zone 2 |  |  |  | 0.4292 |
| Si | Marginal II Zone 3 | Lateral Zone 4 |  |  |  | 0.3402 |
| Si | Marginal II Zone 2 | Central Zone 2 |  |  |  | 0.3744 |
| Si | Marginal I Zone 1 | Central Zone 4 |  |  |  | 0.2030 |
| Si | Marginal I Zone 2 | Central Zone 4 |  |  |  | 0.2030 |
| Si | Marginal I Zone 3 | Central Zone 4 |  |  |  | 0.2030 |
| Si | Marginal I Zone 4 | Central Zone 4 |  |  |  | 0.2030 |
| Si | Marginal II Zone 1 | Central Zone 4 |  |  |  | 0.2030 |
| Si | Lateral Zone 2 | Central Zone 4 |  |  |  | 0.2472 |
| Si | Marginal I Zone 1 | Central Zone 2 |  |  |  | 0.3848 |
| Si | Marginal I Zone 2 | Central Zone 2 |  |  |  | 0.3848 |
| Si | Marginal I Zone 2 | Lateral Zone 2 |  |  |  | 0.3848 |
| Si | Marginal I Zone 2 | Lateral Zone 3 |  |  |  | 0.3848 |
| Si | Marginal I Zone 3 | Lateral Zone 3 |  |  |  | 0.3848 |
| Si | Marginal I Zone 4 | Lateral Zone 3 |  |  |  | 0.3848 |
| Si | Marginal II Zone 1 | Central Zone 2 |  |  |  | 0.3848 |
| Si | Marginal II Zone 1 | Central Zone 1 |  |  |  | 0.2963 |
| Si | Marginal II Zone 3 | Central Zone 4 |  |  |  | 0.0896 |
| Si | Lateral Zone 2 | Central Zone 3 |  |  |  | 0.1924 |
| Si | Marginal II Zone 2 | Central Zone 4 |  |  |  | 0.0635 |
| Si | Marginal I Zone 2 | Central Zone 3 |  |  |  | 0.2662 |
| Si | Marginal I Zone 3 | Central Zone 3 |  |  |  | 0.2662 |
| Si | Marginal I Zone 4 | Central Zone 3 |  |  |  | 0.2662 |
| Si | Marginal II Zone 1 | Lateral Zone 1 |  |  |  | 0.2216 |
| Si | Marginal II Zone 2 | Lateral Zone 4 |  |  |  | 0.1246 |
| Si | Marginal II Zone 3 | Lateral Zone 3 |  |  |  | 0.1665 |
| Si | Lateral Zone 1 | Central Zone 2 |  |  |  | 0.1106 |
| Si | Marginal I Zone 1 | Lateral Zone 2 |  |  |  | 0.2466 |
| Si | Marginal II Zone 1 | Lateral Zone 2 |  |  |  | 0.2466 |
| Si | Marginal I Zone 1 | Central Zone 1 |  |  |  | 0.1637 |
| Si | Marginal I Zone 1 | Lateral Zone 1 |  |  |  | 0.1637 |
| Si | Marginal I Zone 1 | Lateral Zone 4 |  |  |  | 0.1625 |
| Si | Marginal II Zone 1 | Lateral Zone 4 |  |  |  | 0.1625 |
| Si | Marginal II Zone 3 | Central Zone 3 |  |  |  | 0.1047 |
| Si | Marginal I Zone 1 | Central Zone 3 |  |  |  | 0.1528 |
| Si | Marginal II Zone 1 | Central Zone 3 |  |  |  | 0.1528 |
| Si | Marginal II Zone 2 | Lateral Zone 3 |  |  |  | 0.0934 |
| Si | Marginal II Zone 2 | Central Zone 3 |  |  |  | 0.0669 |
| Si | Marginal I Zone 1 | Lateral Zone 3 |  |  |  | 0.1475 |
| Si | Marginal II Zone 1 | Lateral Zone 3 |  |  |  | 0.1475 |
| Si | Lateral Zone 1 | Central Zone 4 |  |  |  | 0.0055* |
| Si | Lateral Zone 1 | Central Zone 3 |  |  |  | 0.0037* |

**Supplementary Table 3.** Correlation coefficients, estimated by row-wise method, between the following parameters: hardness, Young’s modulus, Ae (all elements), Ca, Cl, Mg, Na, P, S, and Si.

|  | Young’s modulus, GPa | Hardness, GPa | Ae, % | Ca, % | Cl, % | Mg, % | Na, % | P, % | S, % | Si, % |
| --- | --- | --- | --- | --- | --- | --- | --- | --- | --- | --- |
| Young’s modulus, GPa | - | - | - | - | - | - | - | - | - | - |
| Hardness, GPa | 0.8125 | - | - | - | - | - | - | - | - | - |
| Ae, % | 0.5889 | 0.6487 | - | - | - | - | - | - | - | - |
| Ca, % | 0.4466 | 0.4843 | 0.9523 | - | - | - | - | - | - | - |
| Cl, % | 0.4399 | 0.5103 | 0.9761 | 0.9127 | - | - | - | - | - | - |
| Mg, % | 0.5822 | 0.6440 | 0.9513 | 0.9428 | 0.9090 | - | - | - | - | - |
| Na, % | 0.2861 | 0.3483 | 0.3650 | 0.1330 | 0.3287 | 0.2587 | - | - | - | - |
| P, % | 0.4814 | 0.5560 | 0.9663 | 0.9235 | 0.9761 | 0.9342 | 0.3270 | - | - | - |
| S, % | 0.4863 | 0.6607 | 0.2616 | 0.2420 | 0.0832 | 0.2808 | 0.5718 | 0.0935 | - | - |
| Si, % | 0.0901 | 0.0809 | 0.1501 | 0.1403 | 0.1100 | 0.0080 | 0.0187 | 0.0156 | 0.1450 | - |
